# DNA methylation and body mass index from birth to adolescence: meta-analyses of epigenome-wide association studies

**DOI:** 10.1186/s13073-020-00810-w

**Published:** 2020-11-25

**Authors:** Florianne O. L. Vehmeijer, Leanne K. Küpers, Gemma C. Sharp, Lucas A. Salas, Samantha Lent, Dereje D. Jima, Gwen Tindula, Sarah Reese, Cancan Qi, Olena Gruzieva, Christian Page, Faisal I. Rezwan, Philip E. Melton, Ellen Nohr, Geòrgia Escaramís, Peter Rzehak, Anni Heiskala, Tong Gong, Samuli T. Tuominen, Lu Gao, Jason P. Ross, Anne P. Starling, John W. Holloway, Paul Yousefi, Gunn Marit Aasvang, Lawrence J. Beilin, Anna Bergström, Elisabeth Binder, Leda Chatzi, Eva Corpeleijn, Darina Czamara, Brenda Eskenazi, Susan Ewart, Natalia Ferre, Veit Grote, Dariusz Gruszfeld, Siri E. Håberg, Cathrine Hoyo, Karen Huen, Robert Karlsson, Inger Kull, Jean-Paul Langhendries, Johanna Lepeule, Maria C. Magnus, Rachel L. Maguire, Peter L. Molloy, Claire Monnereau, Trevor A. Mori, Emily Oken, Katri Räikkönen, Sheryl Rifas-Shiman, Carlos Ruiz-Arenas, Sylvain Sebert, Vilhelmina Ullemar, Elvira Verduci, Judith M. Vonk, Cheng-jian Xu, Ivana V. Yang, Hongmei Zhang, Weiming Zhang, Wilfried Karmaus, Dana Dabelea, Beverly S. Muhlhausler, Carrie V. Breton, Jari Lahti, Catarina Almqvist, Marjo-Riitta Jarvelin, Berthold Koletzko, Martine Vrijheid, Thorkild I. A. Sørensen, Rae-Chi Huang, Syed Hasan Arshad, Wenche Nystad, Erik Melén, Gerard H. Koppelman, Stephanie J. London, Nina Holland, Mariona Bustamante, Susan K. Murphy, Marie-France Hivert, Andrea Baccarelli, Caroline L. Relton, Harold Snieder, Vincent W. V. Jaddoe, Janine F. Felix

**Affiliations:** 1 The Generation R Study Group, Erasmus MC, University Medical Center Rotterdam, Room Na-2918, Erasmus MC, PO Box 2040, 3000 CA Rotterdam, the Netherlands; 2 Department of Pediatrics, Erasmus MC, University Medical Center Rotterdam, Rotterdam, the Netherlands; 3 Department of Epidemiology, Erasmus MC, University Medical Center Rotterdam, Rotterdam, the Netherlands; 4 MRC Integrative Epidemiology Unit, University of Bristol, Bristol, UK; 5 Population Health Sciences, Bristol Medical School, University of Bristol, Bristol, UK; 6 University of Groningen, University Medical Center Groningen, Department of Epidemiology, Groningen, the Netherlands; 7 Geisel School of Medicine at Dartmouth, Lebanon, NH USA; 8 ISGlobal, Barcelona, Spain; 9 Universitat Pompeu Fabra (UPF), Barcelona, Spain; 10 CIBER of Epidemiology and Public Health (CIBERESP), Madrid, Spain; 11 Department of Biostatistics, Boston University School of Public Health, Boston, MA USA; 12 Bioinformatics Research Center, North Carolina State University, Raleigh, NC USA; 13 Center for Human Health and the Environment, North Carolina State University, Raleigh, NC USA; 14 Children’s Environmental Health Laboratory, Division of Environmental Health Sciences, School of Public Health, University of California, Berkeley, CA USA; 15 Department of Health and Human Services, Epidemiology Branch, National Institute of Environmental Health Sciences, National Institutes of Health, Research Triangle Park, NC USA; 16 University of Groningen, University Medical Center Groningen, Department of Pediatric Pulmonology and Pediatric Allergy, Beatrix Children’s Hospital, Groningen, The Netherlands; 17 University Medical Center Groningen GRIAC Research Institute, University of Groningen, Groningen, the Netherlands; 18 Institute of Environmental Medicine, Karolinska Institutet, Stockholm, Sweden; 19 Centre for Occupational and Environmental Medicine, Region Stockholm, Stockholm, Sweden; 20 Centre for Fertility and Health, Norwegian Institute of Public Health, Oslo, Norway; 21 Oslo Centre for Biostatistics and Epidemiology, Oslo University Hospital, Oslo, Norway; 22 School of Water, Energy and Environment, Cranfield University, Cranfield, Bedfordshire UK; 23 Human Development and Health, Faculty of Medicine, Southampton General Hospital, University of Southampton, Southampton, UK; 24 School of Pharmacy and Biomedical Sciences, Curtin University, Bentley, Western Australia Australia; 25 School of Biomedical Sciences, The University of Western Australia, Crawley, Western Austalia Australia; 26 Centre for Women’s, Family and Child Health, University of South-Eastern Norway, Kongsberg, Norway; 27 Institute of Clinical Research, University of Southern Denmark, Odense, Denmark; 28 Department of Biomedical Sciences, Faculty of Medicine and Health Sciences, University of Barcelona, Barcelona, Spain; 29 Research group on Statistics, Econometrics and Health (GRECS), University of Girona, Girona, Spain; 30 Division of Metabolic and Nutritional Medicine, Dr. von Hauner Children’s Hospital, Ludwig-Maximilians Universität München (LMU), Munich, Germany; 31 Center for Life Course Health Research, University of Oulu, Oulu, Finland; 32 Department of Medical Epidemiology and Biostatistics, Karolinska Institutet, Stockholm, Sweden; 33 Department of Psychology and Logopedics, Faculty of Medicine, University of Helsinki, Helsinki, Finland; 34 Department of Preventive Medicine, University of Southern California, Los Angeles, CA USA; 35 CSIRO Health and Biosecurity, North Ryde, New South Wales Australia; 36 Department of Epidemiology, Colorado School of Public Health, Aurora, CO USA; 37 Lifecourse Epidemiology of Adiposity and Diabetes (LEAD) Center, University of Colorado Anschutz Medical Campus, Aurora, CO USA; 38 Clinical and Experimental Sciences, Faculty of Medicine, University of Southampton, Southampton, UK; 39 Department of Air Pollution and Noise, Norwegian Institute of Public Health, Oslo, Norway; 40 Medical School, University of Western Australia, Perth, Australia; 41 Department of Translational Research in Psychiatry, Max-Planck-Institute of Psychiatry, Munich, Germany; 42 Department of Psychiatry and Behavioral Sciences, Emory University School of Medicine, Atlanta, GA USA; 43 Department of Preventive Medicine, Keck School of Medicine, University of Southern California, Los Angeles, CA USA; 44 Center for Environmental Research and Children’s Health, School of Public Health, University of California, Berkeley, CA USA; 45 College of Veterinary Medicine, Michigan State University, East Lansing, MI USA; 46 Pediatrics, Nutrition and Development Research Unit, Universitat Rovira i Virgili, IISPV, Reus, Spain; 47 Neonatal Department, Children’s Memorial Health Institute, Warsaw, Poland; 48 Department of Biological Sciences, North Carolina State University, Raleigh, NC USA; 49 Department of Clinical Science and Education, Södersjukhuset, Karolinska Institutet, Stockholm, Sweden; 50 Sachs’ Children and Youth Hospital, Södersjukhuset, Stockholm, Sweden; 51 CHC, St Vincent, Liège-Rocourt, Belgium; 52 Université Grenoble Alpes, Inserm, CNRS, Team of Environmental Epidemiology Applied to Reproduction and Respiratory Health, IAB, Grenoble, France; 53 Department of Obstetrics and Gynecology, Duke University Medical Center, Raleigh, NC USA; 54 Department of Population Medicine, Harvard Medical School, Harvard Pilgrim Health Care Institute, Boston, MA USA; 55 Department of Pediatrics, San Paolo Hospital, University of Milan, Milan, Italy; 56 Department of Gastroenterology, Hepatology and Endocrinology, CiiM, Centre for Individualised Infection Medicine, a joint venture between the Hannover Medical School and the Helmholtz Centre for Infection Research, Hannover, Germany; 57 TWINCORE, Centre for Experimental and Clinical Infection Research, a joint venture between the Hannover Medical School and the Helmholtz Centre for Infection Research, Hannover, Germany; 58 Division of Biomedical Informatics and Personalized Medicine, Department of Medicine, University of Colorado School of Medicine, Aurora, CO USA; 59 Center for Genes, Environment and Health, National Jewish Health, Denver, CO USA; 60 Division of Epidemiology, Biostatistics, and Environmental Health, University of Memphis, Memphis, TN USA; 61 Department of Biostatistics and Informatics, Colorado School of Public Health, Aurora, CO USA; 62 Department of Pediatrics, School of Medicine, University of Colorado Anschutz Medical Campus, Aurora, CO USA; 63 CSIRO Health and Biosecurity, Adelaide, SA Australia; 64 Turku Institute for Advanced Studies, University of Turku, Turku, Finland; 65 Pediatric Allergy and Pulmonology Unit at Astrid Lindgren Children’s Hospital, Karolinska University Hospital, Stockholm, Sweden; 66 Department of Epidemiology and Biostatistics, School of Public Health, Imperial College London, London, UK; 67 Unit of Primary Health Care, Oulu University Hospital, OYS, Oulu, Finland; 68 Department of Life Sciences, College of Health and Life Sciences, Brunel University London, London, UK; 69 Department of Public Health, Section of Epidemiology, and The Novo Nordisk Foundation Center for Basic Metabolic Research, Section on Metabolic Genetics, Faculty of Medical and Health Sciences, University of Copenhagen, Copenhagen, Denmark; 70 Telethon Kids Institute, University of Western Australia, Perth, Australia; 71 David Hide Asthma and Allergy Research Centre, Isle of Wight, UK; 72 Department of Chronic Diseases and Ageing, Norwegian Institute of Public Health, Oslo, Norway; 73 Diabetes Unit, Massachusetts General Hospital, Boston, MA USA; 74 Department of Medicine, Universite de Sherbrooke, Sherbrooke, QC Canada; 75 Department of Environmental Health Sciences, Columbia University Mailman School of Public Health, New York, NY USA

**Keywords:** Body mass index, Childhood obesity, DNA methylation, Epigenetics

## Abstract

**Background:**

DNA methylation has been shown to be associated with adiposity in adulthood. However, whether similar DNA methylation patterns are associated with childhood and adolescent body mass index (BMI) is largely unknown. More insight into this relationship at younger ages may have implications for future prevention of obesity and its related traits.

**Methods:**

We examined whether DNA methylation in cord blood and whole blood in childhood and adolescence was associated with BMI in the age range from 2 to 18 years using both cross-sectional and longitudinal models. We performed meta-analyses of epigenome-wide association studies including up to 4133 children from 23 studies. We examined the overlap of findings reported in previous studies in children and adults with those in our analyses and calculated enrichment.

**Results:**

DNA methylation at three CpGs (cg05937453, cg25212453, and cg10040131), each in a different age range, was associated with BMI at Bonferroni significance, *P* < 1.06 × 10^−7^, with a 0.96 standard deviation score (SDS) (standard error (SE) 0.17), 0.32 SDS (SE 0.06), and 0.32 BMI SDS (SE 0.06) higher BMI per 10% increase in methylation, respectively. DNA methylation at nine additional CpGs in the cross-sectional childhood model was associated with BMI at false discovery rate significance. The strength of the associations of DNA methylation at the 187 CpGs previously identified to be associated with adult BMI, increased with advancing age across childhood and adolescence in our analyses. In addition, correlation coefficients between effect estimates for those CpGs in adults and in children and adolescents also increased. Among the top findings for each age range, we observed increasing enrichment for the CpGs that were previously identified in adults (birth *P*_enrichment_ = 1; childhood *P*_enrichment_ = 2.00 × 10^−4^; adolescence *P*_enrichment_ = 2.10 × 10^−7^).

**Conclusions:**

There were only minimal associations of DNA methylation with childhood and adolescent BMI. With the advancing age of the participants across childhood and adolescence, we observed increasing overlap with altered DNA methylation loci reported in association with adult BMI. These findings may be compatible with the hypothesis that DNA methylation differences are mostly a consequence rather than a cause of obesity.

## Background

An accumulating body of evidence suggests that exposures in early life are associated with childhood BMI [1]. It is hypothesized that changes in DNA methylation may underlie the associations of early-life exposures with childhood adiposity [2–4]. Thus far, most of the evidence regarding DNA methylation and adiposity stems from adult studies [5–9]. The largest epigenome-wide association study (EWAS) in adults identified cross-sectional associations between DNA methylation at 187 loci and BMI in over 10,000 participants [5]. Previous studies of the associations between epigenome-wide DNA methylation and childhood and adolescent adiposity were small and inconclusive [10–16]. Candidate gene studies in childhood identified associations of DNA methylation in cord and childhood blood with measures of adiposity [17–24]. Epigenome-wide association studies in children and adolescents, with sample sizes ranging from 40 to 700 individuals, identified a limited number of cytosine-phosphate-guanine sites (CpGs) associated with BMI [11–13, 15, 25]. Although findings of some studies suggest that differences in DNA methylation may precede the development of adiposity, recent studies in adults, using methods such as Mendelian randomization, posit that alterations in DNA methylation are predominantly the consequence of adiposity, rather than the cause [4, 5, 9, 26, 27]. The direction of any causal pathway has not been robustly appraised in children. Obtaining more knowledge on the association between DNA methylation and adiposity already in childhood may have implications for future prevention of obesity and its related traits.

We performed a meta-analysis of epigenome-wide association studies of BMI in up to 4133 participants from 23 studies. We assessed associations of DNA methylation in cord blood, in childhood and adolescence with BMI in children aged 2–18 years. We also compared the effect estimates and examined whether there was enrichment in our data for CpGs previously identified for their association with adolescent and adult adiposity.

## Methods

### Participants

We meta-analyzed epigenome-wide association studies of cord or whole blood methylation with childhood or adolescent body mass index (BMI). We used data from up to 4133 participants from 23 studies collaborating in the Pregnancy And Childhood Epigenetics (PACE) Consortium, LifeCycle Project, and NutriProgram Project (Additional file 1: Table S1A-D and Additional file 2: Supplementary Methods) [28, 29]: ALSPAC, BAMSE, CHAMACOS, CHOP Study, CHS, DOMInO Trial, GECKO Drenthe cohort, Generation R Study, GOYA study, Healthy Start Study, HELIX, INMA, IOW F1, IOW F2, MoBa1, MoBa2, NEST, NFBC 1986, PIAMA study, PREDO study, Project Viva, Raine, and STOPPA (full names in Supplementary Methods). Cohort participants were mainly of European ancestry, but there were also cohorts with (partly) non-European ethnicities (African, Hispanic, and Aboriginals). Most cohorts are prospective birth cohorts. We excluded multiple births, siblings (maximum one child per family), physician-diagnosed syndromic obesity cases, and any type of maternal diabetes (including gestational diabetes). Informed consent was obtained for all participants, and all studies received approval from their local ethics committees (see Additional file 2: Supplementary Methods).

### DNA methylation

DNA methylation was measured in cord blood and whole blood samples, in children and adolescents using the Illumina Infinium® HumanMethylation450 BeadChip assay (Illumina, San Diego, CA, USA) [30]. Each cohort independently conducted their preferred quality control and normalization method, see Additional file 2: Supplementary Methods for details. Untransformed normalized beta values of individual CpG sites were used as exposure variables. If multiple measurements of DNA methylation and BMI were available within an age range, we used the oldest age within that range for which BMI and DNA methylation were available at the same time point. Outlying methylation beta values were excluded using the following method: values < (25th percentile − 3*interquartile range (3IQR)) and values > (75th percentile + 3IQR) were removed [31]. DNA methylation is expressed as the proportion of alleles at which the DNA was methylated at a specific site and hence takes values from zero to one.

### Childhood BMI

Height and weight were measured in each study using established protocols as described in detail in the Additional file 2: Supplementary Methods. The primary outcome was BMI, calculated as weight/height^2^ in kg/m^2^, on a continuous scale measured in three age ranges: 2–5 years (early childhood), 5–10 years (late childhood), and 14–18 years (adolescence). If multiple BMI and DNA methylation measurements were available, we used the measurements at the oldest age within the age range for which BMI and DNA methylation were available at the same time point. BMI values were then transformed into sex- and age-adjusted standard deviation scores (SDS) using LMSGrowth [32–34]. The International Obesity Task Force (IOTF) standard was used to define cutoffs for BMI for underweight, normal weight, overweight, and obesity in children, created with the British 1990 growth reference and information of participants on BMI, sex, and age [35, 36]. In secondary analyses, we used a binary outcome variable with normal-weight children as controls and overweight or obese children as cases. Underweight children were excluded from these secondary analyses. If a study had ≤ 10 participants in one of the (case or control) groups, this study was excluded from the secondary analyses.

### Covariates

Covariates included in all models were maternal covariates: maternal age, maternal educational level (cohort definition), maternal smoking status during pregnancy (any smoking versus no smoking), maternal pre-pregnancy or early pregnancy BMI and parity (multiparous versus nulliparous), and gestational age at birth. For details on cohort-specific collection methods, see Additional file 2: Supplementary Methods. We estimated white blood cell proportions (B cells, CD8+ T cells, CD4+ T cells, granulocytes, NK cells, and monocytes) using the reference-based Houseman method with the Reinius reference in the minfi package in R [37–40]. A sensitivity analysis using the cord blood-specific Bakulski reference was performed in the Generation R and ALSPAC studies [41]. Batch effects were adjusted for using cohort-specific methods, see Additional file 2: Supplementary Methods. Additional covariates added in the cross-sectional childhood analyses were birth weight and breastfeeding. The adolescent analyses were additionally adjusted for adolescent age, sex, own smoking status, and puberty status. Puberty status was categorized into early puberty (if both breast and pubic hair Tanner stages (or comparable classification) were 1, 2, or 3 and if girls were pre-menarcheal or boys did not have voice change yet) and late puberty (if either breast or pubic hair Tanner stages (or comparable classification) were 4 or 5 or if girls were post-menarcheal or boys had had their voice change) [42–44]. Further details are provided in the study-specific Additional file 2: Supplementary Methods.

### Study-specific analyses

Associations of DNA methylation with childhood or adolescent BMI were performed in individual studies on participants with complete data on all covariates. In studies with more than one ethnic group, each group was analyzed separately. We used robust linear regression models for the continuous outcome of BMI-SDS and generalized linear regression models for the case/control analyses of overweight and obesity versus normal weight, according to a pre-specified analysis plan. EWAS analyses were conducted using DNA methylation at three time points: birth, childhood and adolescence, and BMI data collected at three time points: early childhood (2–5 years), late childhood (5–10 years), and adolescence (12-18y) (Table 1). We categorized the childhood period into early and late childhood to overcome any age-specific effects and the potential influence of the adiposity rebound on the results [45]. Depending on data availability, cohorts participated in one or more of four analyses: (analysis A) longitudinal associations of cord blood DNA methylation with early childhood BMI (2–5 years; 3295 children from 13 studies), (analysis B) longitudinal associations of cord blood DNA methylation with late childhood BMI (5–10 years; 4133 children from 12 studies), (analysis C) cross-sectional associations of childhood blood DNA methylation with childhood BMI (2–10 years; 3371 children from 11 studies), and (analysis D) cross-sectional associations of adolescent blood DNA methylation with adolescent BMI (14–18 years; 2842 adolescents from 7 studies) (Table 1). Participating studies per analysis are shown in Additional file 1: Table S1A-D.

**Table 1:** Overview of main analyses, secondary analyses, and sensitivity analyses

Analysis	Main analyses			Secondary analyses: binary model (*N*), cases = overweight and obesity, controls = normal weight	Sensitivity analyses	
	DNA methylation in the blood	BMI SD scores	***N***		Europeans only (*N*)	Without studies > 30% overweight and obesity (*N*)
**Cord blood analyses**						
**A**	**Birth (cord blood)**	**Early childhood (2–5 years)**	**3295**	Cases = 491 Controls = 2540	2902	2989
**B**	**Birth (cord blood)**	**Late childhood (5–10 years)**	**4133**	Cases = 707 Controls = 3217	3657	3489
**Cross-sectional analyses**						
**C**	**Childhood (whole blood)**	**Childhood (2–10 years)**	**3371**	Cases = 644 Controls = 2567	3026	3171
**D**	**Adolescence (whole blood)**	**Adolescence (12–18 years)**	**2842**	Cases = 507 Controls = 2188	NA	NA

Analyses A and B were adjusted for maternal age, educational level, smoking status, pre-pregnancy or early pregnancy BMI, parity, gestational age at birth, batch, and estimated cell type proportions

Analyses C was adjusted for maternal age, educational level, smoking status, pre-pregnancy or early pregnancy BMI, parity, gestational age at birth, batch, estimated cell type proportions, birth weight, and breastfeeding

Analyses D was adjusted for maternal age, educational level, smoking status, pre-pregnancy or early pregnancy BMI, parity, gestational age at birth, batch, estimated cell type proportions, birth weight, breastfeeding, adolescent sex, age smoking and puberty status

Cord blood analyses  were adjusted for maternal age, educational level, smoking status, pre-pregnancy or early pregnancy BMI, parity, gestational age, batch, and estimated cell type proportions. The cross-sectional analysis in childhood was additionally adjusted for child covariates birth weight and breastfeeding; in contrast, the cross-sectional analysis in adolescence was adjusted for the same covariates as analysis C plus adolescent sex, age, and smoking and puberty status.

### Meta-analyses

After performing quality control on all studies, we combined results in a fixed-effects inverse variance-weighted meta-analysis using METAL [46, 47]. All follow-up analyses were conducted in R [39]. The meta-analyses were done independently by two study groups, and the results were compared. After exclusion of probes that were measured in only one study, that mapped to X and Y chromosomes and probes that co-hybridized to alternate sequences (cross-reactive probes), we included 429,959 probes for analysis A, 429,959 probes for analysis B, 429,957 probes for analysis C, and 428,967 probes for analysis D [48, 49]. In the result files of the main meta-analyses, we flagged probes that map to DNA containing a single nucleotide polymorphism (SNP), to repetitive sequence elements, or to DNA harboring an INDEL (Additional file 3: Table S2A-D) [48, 49]. We corrected for multiple testing using both the Bonferroni correction, which gives a significance threshold of *P* < 1.16 × 10^−7^ (0.05/429,959), and the less stringent false discovery rate (FDR) threshold using the method by Benjamini and Hochberg [50]. EWAS results were summarized as mean (and standard error) differences in BMI-SDS per 10% increase in methylation for each CpG. We created volcano plots to visualize magnitude and direction of effect (reduced or increased methylation) along with the level of statistical significance. We calculated the *I*^2^ statistic to explore heterogeneity across studies. The *I*^2^ estimates the proportion of variation in the meta-analysis results for each CpG site that is due to between-study differences rather than random/sampling variation. Heterogeneity was defined as an *I*^2^ value of > 50 and shown graphically in forest plots. We performed leave-one-out analyses, in which we reran the main meta-analysis repeatedly with one of the 23 studies removed each time, to explore if any study influenced individual findings. We enhanced the annotation provided by Illumina using the UCSC Genome Browser. All of the annotations use the human February 2009 (GRCh37/hg19) assembly. We updated the gene names manually in all result files using HUGO gene nomenclature, and in case they were not found there, we used the NCBI gene website on November 5, 2019 [51–53].

To explore the associations for the extreme upper values of the BMI distribution, we performed case/control analyses (overweight and obesity versus normal weight). Underweight children were excluded from these analyses, leading to sample sizes of *N* = 491 cases and 2540 controls (analysis A), *N* = 707 cases and 3217 controls (analysis B), *N* = 644 cases and 2567 controls (analysis C), and *N* = 507 cases and 2188 controls (analysis D) (Additional file 4: Table S3A-D).

To examine whether any of the Bonferroni-significant or FDR-significant CpGs in our analyses were close to BMI SNPs, we assessed if these CpGs were located within a 4-Mb window (± 2 Mb) surrounding the 15 genetic loci associated with childhood body mass index [2, 54]. For the FDR-significant CpGs that were flagged because they were potentially influenced by a SNP, we visually inspected density plots in the Generation R Study to see whether these deviated from unimodality (Additional file 5: Supplementary Information, Fig. S6). To explore DNA methylation patterns in the regions around the significant CpGs, we assessed the associations of all CpGs located within a 10-kb window (± 5 kb) surrounding these CpGs with BMI in the relevant models (Additional file 6: Table S4).

### Sensitivity analyses

To explore whether ethnic heterogeneity may have affected our results, we repeated the meta-analyses including studies with participants of European ancestry only (*N* = 2902 (excluding three studies for analysis A), *N* = 3657 (excluding three studies for analysis B), *N* = 3026 (excluding two studies for analysis C)), the largest ethnic subgroup (Additional file 7: Table S5A-C). Ethnicity was defined using self-reported questionnaires unless specified otherwise in the study-specific Supplementary Methods (Additional file 2). We performed additional analyses excluding studies with a high percentage (> 30% (percentage calculated after exclusion of underweight children)) of children with overweight and obesity to explore whether any associations found may be driven by more extreme values of BMI (included *N* = 2989 (excluding two studies for analysis A), *N* = 3489 (excluding four studies for analysis B), *N* = 3171 (excluding one study for analysis C) (Additional file 8: Table S6A-C). We also performed a third, conservative, sensitivity analysis in all age groups, excluding cohorts of non-Europeans, studies with a high percentage (> 30%) of children with overweight or obesity, and studies in which the sample was selected on or enriched for any particular exposure or outcome (Additional file 9: Table S7A-D).

### Comparison with previous findings

We explored whether CpG sites associated with childhood, adolescent, or adult BMI in previous studies were associated with BMI in our data. For previous candidate gene studies and smaller EWASs (*N* < 1000), we performed a look-up of the hits, using a Bonferroni-adjusted *P* value cutoff per study, so for each study, the cutoff was 0.05/(*N* CpGs from that study) (Additional file 10: Table S8) [7, 15, 17, 18, 20, 21, 23, 24, 55]. If the specific CpGs from a study were not available in our dataset, we looked up all CpGs annotated to the relevant genes [17, 24]. To establish whether the CpG sites associated with BMI in previously reported larger EWASs (*N* ≥ 1000) were over-represented among our CpGs with the smallest *P* values, we examined the absolute overlap of the top CpGs from literature with the top CpGs in our analyses [5, 6, 9, 25, 26, 56–59]. The latter were defined using two cutoffs: a stringent cutoff of *P* value < 1 × 10^−5^ and a more lenient one of *P* value < 0.05. (Additional file 11: Table S9). We used a hypergeometric test to calculate enrichment with the phyper function in the R Stats package in R.

We examined the 187 CpGs identified in the largest adult study (*N* = 10,261) to date in more detail in our results [5]. We tested whether the enriched CpGs significantly overlapped between our analyses using chi-square tests. We used Pearson’s correlation coefficients to examine the correlations between the effect estimates of these 187 CpGs in adults and those in our analyses [5]. Using Fisher’s exact test, we calculated whether the correlation coefficients at the various ages were significantly different from each other.

### Functional analyses

We explored the potential functional interpretation of the most significantly associated CpGs (*P* value < 1 × 10^−4^) in all models using Gene Ontology (GO) and Kyoto Encyclopedia of Genes and Genomes (KEGG) enrichment analyses. We used the missMethyl package, which enabled us to correct for the number of probes per gene on the 450K array, based on the May 5, 2020, version of the GO and the October 23, 2019, version of the KEGG source databases [60]. To filter out the large, general pathways, we set the number of genes for each gene set between 5 and 2000, respectively. We report nominal *P* values < 0.05 and FDR for enrichment (Additional file 12, Table S10).

## Results

### Participants

We included 2842 to 4133 participants from 23 independent cohorts from the Pregnancy And Childhood Epigenetics (PACE) Consortium [28]. We assessed associations of DNA methylation in cord blood with BMI in early childhood (2–5 years) (*N* = 3295, analysis A), DNA methylation in cord blood with BMI in late childhood (5–10 years) (*N* = 4133, analysis B), DNA methylation in childhood with BMI in childhood (2–10 years) (*N* = 3371, analysis C), and DNA methylation in adolescence with BMI in adolescence (12–18 years) (*N* = 2842, analysis D). Details of participants and studies used in the different analyses are presented in Table 1, Additional file 1: Table S1A-D and Additional file 2: Supplementary Methods.

### Meta-analyses

**Fig. 1:**
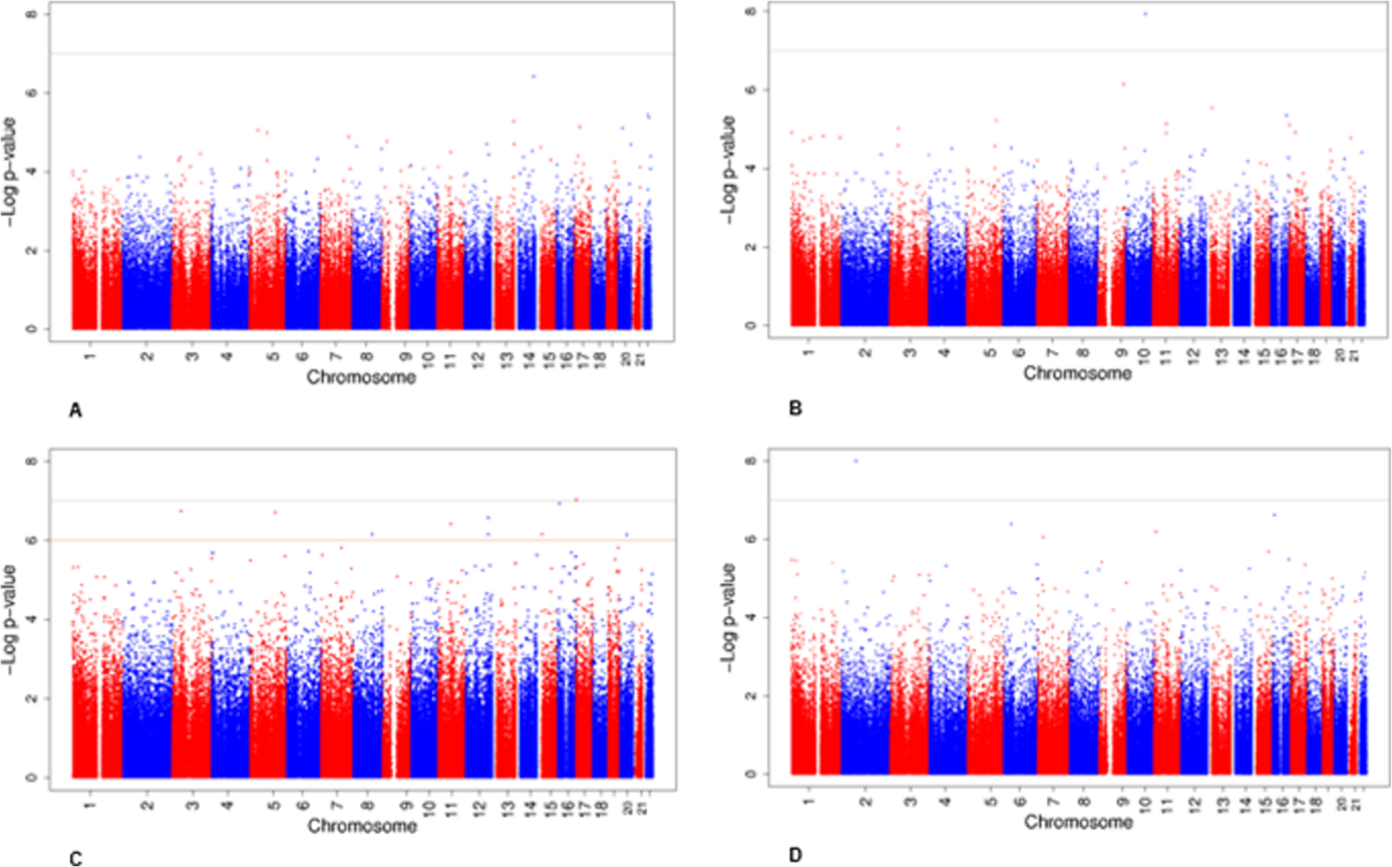
Manhattan plots for the meta-analyses of DNA methylation and childhood or adolescent BMI. Manhattan plots showing the meta-analysis results for associations of DNA methylation in cord blood with early childhood BMI (**a**) and late childhood BMI (**b**), of DNA methylation in whole blood in childhood with childhood BMI (**c**), and of DNA methylation in whole blood in adolescence with adolescent BMI (**d**). The gray line shows the Bonferroni-corrected significance threshold for multiple testing (*P* < 1.06 × 10^−7^). The orange line shows the FDR-corrected significance threshold for multiple testing

The main, secondary, and sensitivity analyses are outlined in Table 1. Genomic inflation factors (lambdas) for the main meta-analyses ranged between 0.97 and 1.27 (Additional file 5: Supplementary information, Fig. 1a-d). Genomic inflation factors (lambdas) of all cohort-specific analyses are shown in Additional file 1: Table S1A-D. The main results are shown in Table 2 and Fig. 1. We did not identify associations at genome-wide significance of DNA methylation in cord blood with BMI in early childhood (analysis A, Fig. 1a, and Additional file 3: Table S2A). DNA methylation at one CpG, cg05937453 (*SFRP5*), in cord blood was significantly associated with late-childhood BMI (analysis B, Fig. 1b, and Additional file 3: Table S2B). For each 10% increase in DNA methylation at cg05937453 in cord blood, late-childhood BMI increased 0.96 SD (standard error (SE) 0.17). Cord blood DNA methylation at this CpG was nominally significantly associated with BMI in early-childhood (*P* value = 0.004), but DNA methylation in childhood and adolescence was not associated with BMI in the cross-sectional analyses (Additional file 13: Table S11).

**Table 2:** CpG sites at which DNA methylation was associated with child or adolescent BMI

CpG	CHR	Location	Coef	SE	***P*** value	FDR ***P*** value	Nearest gene
**Analysis B = association of cord blood DNA methylation with late childhood BMI (5–10 years)**							
cg05937453	10	99531765	0.96288	0.16871	1.15 × 10^−8^	0.0049	*SFRP5*
**Analysis C = cross-sectional association of whole blood DNA methylation with childhood BMI (2–10 years)**							
cg25212453	17	1509953	0.31925	0.05978	9.27 × 10^−8^	0.02075	*SLC43A2*
cg03500056	16	8814507	0.30577	0.05767	1.15 × 10^−7^	0.02075	*ABAT*
cg05281708	3	44690673	0.65856	0.12614	1.78 × 10^−7^	0.02075	*ZNF35*
cg15125798	5	122621645	0.49705	0.09548	1.93 × 10^−7^	0.02075	–
cg04456029	12	113496126	0.27587	0.05358	2.63 × 10^−7^	0.0226	*DTX1*
cg03431111	11	62621406	0.19261	0.03791	3.77 × 10^−7^	0.0270	*SNORD30*; *SNORD22*; *SNORD29*; *SNORD31*; *SNHG1*
cg26889953	15	22915992	0.31743	0.06391	6.81 × 10^−7^	0.0304	*CYFIP1*
cg19743522	12	113495566	0.33854	0.0682	6.92 × 10^−7^	0.0304	*DTX1*
cg25877069	8	95003236	− 0.45126	0.09092	6.94 × 10^−7^	0.0304	–
cg13931559	20	33146515	− 0.84718	0.17082	7.07 × 10^−7^	0.0304	*MAP1LC3A*
**Analysis D = cross-sectional association of whole blood DNA methylation with adolescent BMI (12–18 years)**							
cg10040131	2	73178866	0.32434	0.0566	1.00 × 10^−8^	0.0043	*SFXN5*

Coefficients (Coef) and standard errors (SE) are presented per 10% increase in the methylation level

Analyses B was adjusted for maternal age, educational level, smoking status, pre-pregnancy or early pregnancy BMI, parity, gestational age, batch, and estimated cell type proportions. Analysis C was additionally adjusted for child covariates birth weight and breastfeeding, whereas analysis D was adjusted for the same covariates as analysis C plus adolescent sex, age, smoking, and puberty status

In the cross-sectional analysis (analysis C), childhood DNA methylation at cg25212453 (*SLC43A2*) was associated with childhood BMI after Bonferroni correction. A 10% increase in DNA methylation at cg25212453 was associated with a 0.32 SD (SE 0.06) increase in childhood BMI (Fig. 1c and Additional file 3: Table S2C). DNA methylation at this CpG at birth and in adolescence was not associated with BMI (Additional file 13: Table S11). DNA methylation in childhood at nine additional CpGs in or near other genes was associated with childhood BMI using FDR *P* value < 0.05 (Fig. 1c and Additional file 3: Table S2C). DNA methylation in adolescence at cg10040131 (*SFXN5*) was associated with adolescent BMI after Bonferroni correction (analysis D, Fig. 1d and Additional file 3: Table 2d). A 10% increase in DNA methylation at cg10040131 was associated with a 0.32 SD (SE 0.06) higher BMI in adolescence. DNA methylation at this CpG in childhood was nominally significantly associated with childhood BMI (*P* value = 0.0002). The association of DNA methylation at this CpG in cord blood and BMI in childhood was not significant (Additional file 13: Table S11).

Associations of DNA methylation with BMI did not show a preferential direction of effect in any of the analyses (volcano plots, Additional file 5: Supplementary Information, Fig. S2A-D). We observed very little evidence of heterogeneity between studies among the Bonferroni-significantly associated CpG sites, with all *I*^2^ ≤ 50 (Additional file 3: Table 2a-d and forest plots, Additional file 5: Supplementary Information, Fig. S3A, B and L). We found evidence of between-study heterogeneity (*I*^2^ > 50) for 3 of the 9 FDR-significantly associated CpG sites (Additional file 3: Table 2c and forest plots, Additional file 5: Supplementary Information, Fig. S3C-K). The results for the twelve Bonferroni or FDR-significantly associated CpGs were stable after omitting one study at a time (leave-one-out analyses, Additional file 5: Supplementary Information, Fig. S4A-L).

When BMI was dichotomized into normal and overweight/obesity, only one CpG in the cross-sectional model in childhood, cg06991974 (*PRDM16-DT*), showed evidence of association. In the cross-sectional model during childhood, which included 644 children with overweight/obesity and 2567 normal-weight children, DNA methylation at cg06991974 was associated with an increased risk of overweight/obesity in childhood (odds ratio (OR) 3.10, 95% confidence interval (CI) 2.08, 4.63) (Additional file 4: Table S3A-D).

None of the three individual Bonferroni-significant CpGs in the three different age ranges nor the 9 FDR-significant CpGs was within a 4-Mb window surrounding the 15 known genetic loci associated with childhood body mass index [54].

Four of the 12 FDR significant CpGs contained a single-nucleotide polymorphism (SNP) [48, 49]. We found no indication of non-unimodal distribution for any of these CpGs suggesting that methylation measurements at these sites were not markedly affected by SNPs (Additional file 5: Supplementary Information, Fig. S6).

Two of the three Bonferroni-significant CpGs (cg05937453 and cg25212453) had other nearby CpGs within a 10-kb window (± 5 kb) measured on the 450K array (Additional file 6: Table S4). Cg05937453 (model B) was surrounded by 24 other CpGs, of which one was nominally significantly associated with BMI (*P* value < 0.05). Both were located in the TSS200 region of *SFRP5* with effect estimates in the same direction. Cg25212453 (model C) was surrounded by 13 other CpGs, of which three were nominally significant (*P* values < 0.05). All were located in the gene body of *SLC43A2* with effect estimates in the same direction. Results for Bonferroni- and FDR-significant CpGs are shown in Additional file 6: Table S4.

### Sensitivity analyses

Findings were consistent with the main results when restricted to up to 3657 participants of European ethnicity (Pearson correlation coefficients of the effect estimates across all CpG sites were 0.86–0.97 and were 0.99 across top CpG sites (*P* value < 1 × 10^−4^) for all models (Additional file 7: Table S5A-C)). Similarly, when the studies with a high percentage (> 30%) of children with overweight or obesity were excluded, the results were also consistent with the main analyses (Pearson correlation coefficients of the effect estimates across all CpG sites were 0.89–0.98 and were 0.99 across top CpG sites (P value < 1 × 10^−4^) for all models (Additional file 8: Table S6A-C)). Lastly, when the studies of non-Europeans participants, a high percentage of children with overweight or obesity and studies in which the sample was selected on or enriched for any particular exposure or outcome were all excluded, results remained strongly correlated to those from the main models. Pearson correlation coefficients of the effect estimates across all CpG sites were 0.64–0.97 and 0.95–0.99 across top CpG sites (*P* value < 1 × 10–4) for all models (Additional file 9: Table S7A-D).

### Comparison with previous findings

Most CpGs identified to be associated with BMI in previous candidate gene studies or smaller EWASs (*N* < 1000) did not replicate in our results (Additional file 10: Table S8) [7, 15, 17, 18, 20, 21, 23, 24, 55]. When comparing the genome-wide significant findings from the largest BMI EWASs (*N* > 1000) in adults to our most significant findings across the four age ranges, we found an increasing overlap with age (Table 3 and Additional file 11: Table S9) [5, 6, 9, 25, 26, 56–59]. We used two cutoffs to select the most significant findings in our results: a *P* value < 1 × 10^−5^, to identify “suggestive” findings, and a less stringent, nominal *P* value < 0.05. The number of CpGs that met these criteria are provided in Table 3. First, we examined the absolute number of overlapping CpGs between the studies in adults and our findings with a *P* value < 1 × 10^−5^ and calculated enrichment. With advancing age across childhood and adolescence, we observed increasing enrichment for the 187 CpGs previously reported to be associated with adult BMI in the largest study to date (*N* = 10,261) [5]. For the two cord blood models, there was no overlap with the adult findings (*P*_enrichment_ = 1), for the cross-sectional model in childhood 2/187 adult hits overlapped, (*P*_enrichment_ = 0.0002), and for the cross-sectional model in adolescence 3/187 overlapped (*P*_enrichment_ = 2.10 × 10^−7^) (Table 3 and Additional file 11: Table S9). Using the less stringent cutoff (*P* value < 0.05), this trend was even clearer. The overlap between the 187 CpGs from the adult EWAS and the CpGs in our data with a *P* value < 0.05 was 8/187 CpGs (*P*_enrichment_ = 0.77, analysis A) for the association of cord blood DNA methylation and early childhood BMI and 11/187 CpGs (*P*_enrichment_ = 0.30, analysis B) for the association of cord blood DNA methylation and late childhood BMI. For the cross-sectional model in childhood, the overlap was 61/187 CpGs (*P*_enrichment_ = 1.97 × 10^−20^, analysis C), and in adolescence, the overlap was 77/187 CpGs (*P*_enrichment_ = 1.68 × 10^−44^, analysis D) (Table 3 and Additional file 11: Table S9). Twenty-seven CpGs were among the enriched CpGs in both the childhood and the adolescent model. This overlap was not significant (*P* = 0.88).

**Table 3:** Absolute number of overlapping CpGs and *P* values for the enrichment of significant CpGs from previous EWASs (*N* > 1000) in our data

Previous study (***N*** sites associated with BMI)	Significance level	Analysis A: association of cord blood DNA methylation with early childhood BMI (2–5 years)	Analysis B: association of cord blood DNA methylation with late childhood BMI (5–10 years)	Analysis C: cross-sectional analysis of whole blood DNA methylation with childhood BMI (2–10 years)	Analysis D: cross-sectional analysis of whole blood DNA methylation with adolescent BMI (12–18 years)
	1 × 10^−5^	***N*** **= 7**	***N*** **= 8**	***N*** **= 51**	***N*** **= 26**
	0.05	***N*** **= 22,687**	***N*** **= 20,645**	***N*** **= 37,074**	***N*** **= 25,292**
Ali et al. [56] (3 CpGs)	1 × 10^−5^	0 *P*_enrichment_ = 1	0 *P*_enrichment_ = 1	0 *P*_enrichment_ = 1	0 *P*_enrichment_ = 1
	0.05	0 *P*_enrichment_ = 1	0 *P*_enrichment_ = 1	1/3 *P*_enrichment_ = 0.24	0 *P*_enrichment_ = 1
Aslibekyan et al. [6] (8 CpGs)	1 × 10^−5^	0 *P*_enrichment_ = 1	0 *P*_enrichment_ = 1	0 *P*_enrichment_ = 1	0 *P*_enrichment_ = 1
	0.05	0 *P*_enrichment_ = 1	0 *P*_enrichment_ = 1	1/8 *P*_enrichment_ = 0.51	2/8 *P*_enrichment_ = 0.08
Campanella et al. [57] (26 CpGs)	1 × 10^−5^	0 *P*_enrichment_ = 1	0 *P*_enrichment_ = 1	0 *P*_enrichment_ = 1	**1/26** ***P***_**enrichment**_ **= 0.002**
	0.05	3/26 *P*_enrichment_ = 0.16	1/26 *P*_enrichment_ = 0.72	**6/26** ***P***_**enrichment**_ **= 0.02**	**11/26** ***P***_**enrichment**_ **= 1.006 × 10**^**−7**^
Geurts et al. [58] (310 CpGs)	1 × 10^−5^	0 *P*_enrichment_ = 1	0 *P*_enrichment_ = 1	**2/310** ***P***_**enrichment**_ **= 0.0006**	**2/310** ***P***_**enrichment**_ **= 0.0002**
	0.05	12/310 *P*_enrichment_ = 0.90	13/310 *P*_enrichment_ = 0.73	**103/310** ***P***_**enrichment**_ **= 3.92 × 10**^**−34**^	**125/310** ***P***_**enrichment**_ **= 6.63 × 10**^**−70**^
Mendelson et al. [9] (83 CpGs)	1 × 10^−5^	0 *P*_enrichment_ = 1	0 *P*_enrichment_ = 1	**2/83** ***P***_**enrichment**_ **= 4.67 × 10**^**−5**^	**3/83** ***P***_**enrichment**_ **= 1.81 × 10**^**−8**^
	0.05	4/83 *P*_enrichment_ = 0.64	**8/83** ***P***_**enrichment**_ **= 0.045**	**28/83** ***P***_**enrichment**_ **= 1.36 × 10**^**−10**^	**45/83** ***P***_**enrichment**_ **= 3.02 × 10**^**−33**^
Sayols-Baixeras et al. [59] (96 CpGs)	1 × 10^−5^	0 *P*_enrichment_ = 1	0 *P*_enrichment_ = 1	0 *P*_enrichment_ = 1	0 *P*_enrichment_ = 1
	0.05	8/96 *P*_enrichment_ = 0.13	**9/96** ***P***_**enrichment**_ **= 0.04**	**24/96** ***P***_**enrichment**_ **= 1.53 × 10**^**−6**^	**30/96** ***P***_**enrichment**_ **= 1.85 × 10**^**−14**^
Sun et al. [26] *black participants* (36 CpGs)	1 × 10^−5^	0 *P*_enrichment_ = 1	0 *P*_enrichment_ = 1	0 *P*_enrichment_ = 1	**1/36** ***P***_**enrichment**_ **= 0.002**
	0.05	3/36 *P*_enrichment_ = 0.30	**6/36** ***P***_**enrichment**_ **= 0.007**	**13/36** ***P***_**enrichment**_ **= 4.98 × 10**^**−6**^	**22/36** ***P***_**enrichment**_ **= 1.50 × 10**^**−18**^
Sun et al. [26] *white participants* (349 CpGs)	1 × 10^−5^	0 *P*_enrichment_ = 1	0 *P*_enrichment_ = 1	0 *P*_enrichment_ = 1	0 *P*_enrichment_ = 1
	0.05	12/349 *P*_enrichment_ = 0.959	22/349 *P*_enrichment_ = 0.12	**86/349** ***P***_**enrichment**_ **= 4.13 × 10**^**−19**^	**116/349** ***P***_**enrichment**_ **= 1.75 × 10**^**−54**^
Wahl et al. [5] (187 CpGs)	1 × 10^−5^	0 *P*_enrichment_ = 1	0 *P*_enrichment_ = 1	2/187 *P*_enrichment_ = 0.0002	**3/187** ***P***_**enrichment**_ **= 2.10 × 10**^**−7**^
	0.05	8/187 *P*_enrichment_ = 0.77	11/187 *P*_enrichment_ = 0.29	**61/187** ***P***_**enrichment**_ **= 1.97 × 10**^**−20**^	**77/187** ***P***_**enrichment**_ **= 1.68 × 10**^**−44**^
Wang et al. [25] (54 CpGs)	1 × 10^−5^	0 *P*_enrichment_ = 1	0 *P*_enrichment_ = 1	0 *P*_enrichment_ = 1	**1/54** ***P***_**enrichment**_ **= 0.003**
	0.05	2/54 *P*_enrichment_ = 0.79	4/54 *P*_enrichment_ = 0.26	**23/54** ***P***_**enrichment**_ **= 2.49 × 10**^**−11**^	**33/54** ***P***_**enrichment**_ **= 3.98 × 10**^**−27**^
*N* CpGs in ≥ 2 adult studies	0.05	9/52 (17.3%)	23/75 (30.7%)	98/347 (28.2%)	163/465 (35.1%)

Two cutoffs were used to select the significant findings in our results: a *P* value < 1 × 10^−5^, to identify “suggestive” findings, and a less stringent, nominal *P* value < 0.05, to identify any trends. We used a hypergeometric test to calculate enrichment with the phyper function in the R Stats package in R. Results in bold are nominally significant. Of those findings from adult studies that had a nominal *P* value (< 0.05) in our models, 17–35% were reported by more than one adult study

Correlation coefficients between the effect estimates of the 187 hits and the effect estimates for those CpGs in the four models increased with age (analysis A = − 0.186 (*P* = 0.01), analysis B = − 0.013 (*P* = 0.86), analysis C = 0.604 (*P* = 5.31 × 10^−20^), and analysis D = 0.816 (*P* = 7.89 × 10^−46^). The difference in correlation coefficients was significant for all comparisons (*P*’s for comparison between correlation coefficients < 0.01) except for the comparison between models A and B (*P* = 0.09).

Effect sizes of the associations for these 187 adult BMI CpGs in our analyses increased with advancing age of children in our analyses (Additional file 5: Supplementary Information, Fig. S5). We found similar trends for enrichment of CpGs from other EWASs in adults and adolescents (Table 3) [6, 9, 25, 26, 56–59]. Of those findings from adult studies that had a nominal *P* value (< 0.05) in our models, 17–35% were reported by more than one adult study. Most of these were found in two or three studies, but four, cg06500161, cg19750657, cg12593793, and cg18181703, were reported in six or seven previous analyses.

### Functional analyses

A functional enrichment analysis using genes linked to the CpGs with *P* values < 1 × 10^−4^ in each of the models showed no functional enrichment of Gene Ontology (GO) terms or Kyoto Encyclopedia of Genes and Genomes (KEGG) terms (FDR < 0.05) (Additional file 12: Table S10).

## Discussion

In this large meta-analysis of EWASs of childhood and adolescent BMI, we found little evidence of an association between DNA methylation and childhood or adolescent BMI. DNA methylation at three different CpGs, each one in a different age range, was associated with BMI in early life. With the advancing age of children in our analyses, we observed increasing enrichment of CpGs previously identified for their relation with adolescent or adult adiposity. In addition, for the 187 CpGs identified in the largest previous study of adult BMI, we found increasing effect sizes and increasing correlations between the adult effect sizes and those in our analyses, with age.

### Interpretation of main findings

Childhood obesity is a major public health problem and associated with short- and long-term morbidity and mortality [61]. Although there is some evidence from previous studies that DNA methylation may mediate associations of pregnancy-related exposures with offspring adiposity, only few specific CpG sites have been identified [4, 27]. Thus far, most of the evidence for associations of DNA methylation with adiposity stems from adult studies.

In this study, we found little evidence of an association between DNA methylation and childhood or adolescent BMI. DNA methylation at three CpGs (cg05937453, cg25212453, and cg10040131), each in a different age range, was associated with BMI at Bonferroni significance, *P* < 1.06 × 10^−7^. However, we did observe increasing enrichment and increasing point estimates of CpGs previously reported in relation to adult adiposity, with increasing age of the participants in our study [5, 6, 9, 25, 26, 57–59]. Also, correlation coefficients between effect estimates from the adult study and effect estimates in our models increased with the age of the participants in our study. After exclusion of invariable probes (*N* = 114,204) using an adult reference, the trend of increasing enrichment of CpGs associated with adult adiposity with advancing age remained. This result suggests that probes reported to be invariable in adults did not strongly affect the results of the enrichment analyses [62]. These trends were most clearly seen in the cross-sectional analyses in childhood and adolescence, although there was no significant overlap in the enriched CpGs between the two time points. This trend may partly be explained by a difference in study sample size, age range, and covariates between the models. These findings may indicate that over time, exposure to higher “levels” of BMI may lead to differential DNA methylation. DNA methylation has been shown to be responsive to the environment and could also change in response to metabolic changes and the altered adipokine/cytokine environment associated with a higher BMI [63–65]. Methylation differences may be either induced by the altered environment or result from a cellular selection in this altered environment. If differential DNA methylation is the result of exposure to higher BMI, it may be part of a pathway that links adiposity to metabolic and cardiovascular disease [5, 7]. Several studies have reported that DNA methylation levels at obesity-associated CpG sites were associated with cardio-metabolic factors such as lipids, insulin resistance, and blood pressure [26, 64].

Recent studies, using methods such as Mendelian randomization, suggested that alterations in DNA methylation are predominantly a consequence of adiposity, rather than a cause [5, 7, 9, 26]. In these studies, Mendelian randomization was used to investigate the potential causal relationships, independent of unmeasured confounders, between DNA methylation and BMI using genetic variants as instrumental variables [66, 67]. Although in our study, we cannot determine whether any of the associations are causal, our results may be compatible with this hypothesis. One alternative explanation for the increasing enrichment of CpGs previously reported in relation to adult and adolescent adiposity with age in our data could be that BMI at different ages does not represent the same biological phenotype. The DNA methylation profile may simply reflect the transition of childhood BMI into a different, more adult-like BMI phenotype over time. BMI (weight(kg)/height(m^2^)) is likely to have a different biological interpretation at different ages, and with the increase of age, the biological phenotype becomes more similar to adult BMI [68]. DNA methylation at specific CpG sites is known to change with age. We did not see any increased enrichment of age-related CpGs identified in previous childhood and adolescent studies with advancing age in our models (all *P* values > 0.19), making it unlikely that our results represent a strong effect of age [69, 70].

We observed only three CpGs at which DNA methylation in three different age ranges was Bonferroni-significantly associated with BMI in childhood or adolescence. Cg05937453, at which DNA methylation in cord blood was associated with late childhood BMI, is annotated to secreted frizzled-relate protein 5 (*SFRP5*). This gene is part of the *SFRP* family that acts by modulating Wnt signal transduction [71]. The Wnt family and SFRPs have roles in multiple biological processes, including embryonic development, inflammation, and immunity [72]. *SFRP5* is an anti-inflammatory adipokine that may be induced during preadipocyte proliferation, differentiation, and maturation [65, 72]. Less is known about the other two CpGs, cg25212453 and cg10040131, and their potential relation to adiposity. In the cross-sectional analyses in childhood, DNA methylation at cg25212453, in the gene body of solute carrier family 43 member 2 (*SLC43A2*), was associated with BMI. *SLC43A2* transcripts have been described to be associated with fasting insulin in a whole blood transcriptome-wide association analysis of three cohort studies [73]. DNA methylation at cg10040131, located in the gene body of Sideroflexin 5 (*SFXN5*), was associated with BMI in adolescence. *SFXN5* has not been described in relation to adiposity or related phenotypes.

Based on histone marks mapped by Roadmap Epigenomics Data Complete Collection extracted from the UCSC Genome Browser, all 3 CpG sites coincide with a region of weak transcription in blood, and 2 CpG-sites coincide with a region of weak transcription in adipose tissue, except for cg25212453 (at *SLC43A2*) which coincides with an enhancer in adipose tissue [74]. This overlap with key regulatory elements may indicate that DNA methylation at these CpGs could have regulatory consequences [75, 76].

Many previous studies that examined the associations between DNA methylation and childhood BMI were not genome-wide, were of modest sample size, or used only FDR or less stringent cutoffs for significance [10–13, 18, 77]. Previous candidate gene studies reported that methylation of CpGs annotated to proopiomelanocortin (*POMC*), retinoid X receptor alpha (*RXRA*), and nitric oxide synthase 3 (*NOS3* or *eNOS*) was associated with BMI in childhood [17, 24]. The exact CpGs from those studies were either not given or were not present on the 450K Illumina array and could thus not be examined in our data. However, none of the CpGs in our dataset that annotated to these genes was associated with BMI in our analyses [17, 24]. Also, methylation at CpGs in hypoxia-inducible factor 3A (*HIF3A*), previously reported to be differentially methylated in relation to BMI in adults and children, did not show any association with BMI in childhood or adolescence in our data [7, 20, 21, 23]. This finding is in concordance with two recently published studies, both in approximately 1000 participants, which did not find an association between childhood BMI and methylation at *HIF3A* [21, 22].

### Strengths and limitations

This EWAS is much larger than the previous genome-wide studies of the association between DNA methylation and BMI in childhood and adolescence. Other strengths of this study are the extensive analyses from 2 to 18 years, both longitudinal and cross-sectional. We also used a harmonized analysis plan and robust methods in the PACE Consortium. However, compared to studies in adults, the sample size of this meta-analysis is still modest. All participating studies used the Infinium Human Methylation 450K array, which covers only 1.7% of all CpG sites in the genome [78]. Thus, we cannot exclude that methylation at other, non-measured CpGs could be associated with childhood BMI. The 450K BeadChip has now been replaced by the EPIC BeadChip which includes > 850,000 CpG sites (Illumina, San Diego, CA, USA) [78, 79]. Some previous literature included one of the participating studies in this manuscript. We analyzed the associations between DNA methylation and BMI at different times in childhood and adolescence but did not study longitudinal changes in DNA methylation in the same individuals from early life until adulthood in relation to BMI. A recent study among 1485 adults performed cross-lagged analyses of DNA methylation and BMI, both measured at two time points [26]. These analyses showed significant unidirectional paths from BMI to DNA methylation, in line with other, cross-sectional adult studies [5, 7]. We used blood to measure DNA methylation patterns in relation to BMI, which may not be the most relevant tissue. As overweight and obesity are associated with an inflammatory phenotype in the blood and may affect the white blood cell composition, blood may be a relevant target tissue [80]. However, there are many potentially relevant target tissues related to BMI, including the brain, adipocytes, pancreas, liver, and many others, and associations of DNA methylation with BMI may differ between these tissues. In large population-based studies, it is virtually impossible to collect samples from these tissues. A study among adults examined whether the associations of DNA methylation at a specific CpG in blood and adipose tissue in relation to BMI were comparable and showed similar findings between the tissues [7]. We adjusted our childhood and adolescent analyses for estimated cell type proportions using an adult reference dataset, which is likely not an optimal way to adjust for white blood cell proportions at these ages. However, to the best of our knowledge, no childhood- or adolescent-specific reference panels exist [37, 40]. Thus, we may have been unable to fully account for potential differences in the biology of blood at the different ages, which may have had some influence on our results. Specific cord blood reference datasets only became available after completion of the cohort-specific analyses [41, 81]. However, we observed no substantial differences in results in two of our largest studies, Generation R (maximum *N* = 789) and ALSPAC (maximum *N* = 669), when comparing our main analyses using the adult reference with the same analyses using cell counts estimated with a cord blood-specific reference panel [37, 41]. Correlation coefficients of the effect estimates of the analyses using the adult and cord blood-specific reference panel across all 450K CpG sites were *r* = 0.98 and *r* = 0.89, respectively. Childhood BMI is influenced by genetic, prenatal, and postnatal environmental factors. We adjusted for a large number of potential confounding factors. However, residual confounding due to other, non-measured factors might still be present. Individual studies contributing to this meta-analysis performed their own preferred quality control and methylation normalization process. We have previously shown that this does not have a large effect on the associations of interest compared to the use of non-normalized methylation data [82]. Meta-analyzing the results of 23 studies may introduce between-study heterogeneity. We ran multiple sensitivity analyses, which showed results that were comparable with the main findings. Based on *I*^2^ values, most top CpGs did not show large between-study heterogeneity, although three FDR-significant findings did. These three CpG sites had *I*^2^ values of 50.2, 52.7, and 61.8. Forest plots and leave-one-out plots did not show large heterogeneity or an extreme effect of one study (forest plots (Additional file 5: Supplementary Information, Fig.S3H, I and K and Fig. S3H, I and K). The current analyses cannot determine whether any of the associations are causal. Future research using methods such as Mendelian randomization could shed further light on causality, already used by some studies in adults [5, 9, 21, 83]. Analyzing associations of BMI with DNA methylation assessed with the EPIC BeadChip could provide new insights, as it interrogates almost twice the number of CpG sites compared to the 450K BeadChip, and particularly focuses on CpG sites in potential regulatory regions [78, 79]. Also, bisulfite sequencing methods to measure DNA methylation could provide more detailed information. In the current study, we analyzed differential methylation at single CpGs. Future studies could analyze regional patterns of differential methylation (differentially methylated regions (DMRs)) and their associations with BMI to provide further biological insights. We studied BMI mostly in general population samples. If exposure to overweight already changes the DNA methylation profile in childhood or adolescence, it would be interesting to analyze the associations in a population with a more extreme phenotype of obesity in childhood or adolescence. To examine the effects of potential interventions, studies of DNA methylation before and after weight loss in children or adolescents could be useful. In adults, weight loss has been shown to be associated with significantly different DNA methylation patterns [84–86]. Analyzing longitudinal trajectories of DNA methylation and BMI at various time points in the same population from birth to adolescence would help to understand further the biological relevance of DNA methylation level changes and patterns of change [26, 87].

## Conclusions

In this large epigenome-wide association study meta-analysis among children and adolescents, we observed little evidence for associations between DNA methylation at individual CpGs and childhood or adolescent BMI. With advancing age across childhood and adolescence, we observed increasing effect estimates, increasing correlations between adult effect sizes and those in our analyses, and increasing enrichment of CpGs previously identified for their associations with adult adiposity. These findings may be compatible with the hypothesis that DNA methylation differences are mostly a consequence rather than a cause of obesity, but this remains to be confirmed.

## Supplementary Information

**Additional file 1:** **Table S1A-D**. Characteristics of the participating studies in the main meta-analyses.

**Additional file 2:** **Supplementary Methods**. Study-specific funding, acknowledgements and methods in alphabetical order, including references.

**Additional file 3:** **Table S2A-D**. Associations between DNA methylation and BMI in the main meta-analyses. Results for CpGs with *p*-values < 1x10^-4^ are shown.

**Additional file 4:** **Table S3A-D**. Associations between DNA methylation levels and overweight/obesity versus normal weight. Results for CpGs with p-values < 1x10^-4^ are shown.

**Additional file 5:** **Supplementary Information. Figures S1 – 6**.

**Additional file 6:** **Table S4.** CpGs within a window of +/- 5kb from the Bonferroni- and FDR-significant CpGs in each model.

**Additional file 7:** **Table S5A-C.** Associations between DNA methylation and BMI in children of European ancestry only. Results for CpGs with p-values < 1x10^-4^ are shown.

**Additional file 8:** **Table S6A-C.** Associations between DNA methylation and BMI in children, excluding studies with > 30% overweight or obesity. Results for CpGs with p-values < 1x10^-4^ are shown.

**Additional file 9:** **Table S7A-D.** Associations between DNA methylation and BMI in childhood and adolescence, excluding studies with participants of non-European ancestry, those with a high percentage (>30%) of children with overweight/obesity, and, finally, studies with a population for this analysis that was selected based on a particular exposure or outcome.

**Additional file 10:** **Table S8.** Look-up of the CpGs associated with BMI in previously reported candidate gene studies and smaller EWAS (*N* < 1000).

**Additional file 11:** **Table S9.** Results of enrichment analyses for CpGs associated with BMI in previously reported large EWAS (*N*≥1000).

**Additional file 12:** **Table S10.** Results of functional enrichment analyses using genes linked to the CpGs with p-values < 1-x10^-4^ in each of the models using Gene Ontology (GO) terms or Kyoto Encyclopedia of Genes and Genomes (KEGG) terms (FDR <0.05).

**Additional file 13:** **Table S11.** Associations between DNA methylation at the three Bonferroni-significant CpG-sites with BMI in all age ranges.

## Data Availability

Genome-wide DNA methylation meta-analysis summary statistics corresponding to the main analyses presented in this manuscript are available at figshare (10.6084/m9.figshare.13172873) [88]. Individual cohort level data may be available by application to the relevant institutions after obtaining the required approvals. Information on the study cohorts that contributed is available in Additional file 2: Supplementary Methods.

## Abbreviations

BMI: Body mass index
CpG: Cytosine-phosphate-guanine
DNA: Deoxyribonucleic acid
