# Supplementary material for: DNA methylation and body mass index from birth to adolescence: meta-analyses of epigenome-wide association studies
Source: Genome Med. 2020 Nov 25;12:105. doi: 10.1186/s13073-020-00810-w (PMC7687793; doi:10.1186/s13073-020-00810-w)
Supplement: Supplementary file 2 — Additional file 2: Supplementary Methods. Study-specific funding, acknowledgements and methods in alphabetical order, including references. [file 13073_2020_810_MOESM2_ESM.docx]

**Supplementary Methods**

**DNA methylation and body mass index from birth to adolescence:**

**meta-analyses of epigenome-wide association studies**

Vehmeijer et al.

***Supplementary Note:***

**Supplementary Funding.** Study specific and in alphabetical order

**Supplementary Acknowledgements.** Study specific and in alphabetical order

**Supplementary Methods.** Study specific and in alphabetical order

**Supplementary References**

# Funding: study specific and in alphabetical order

## ALSPAC

The UK Medical Research Council and the Wellcome Trust (Grant ref: 102215/2/13/2) and the University of Bristol provide core support for ALSPAC. The Accessible Resource for Integrated Epigenomics Studies (ARIES) which generated large scale methylation data was funded by the UK Biotechnology and Biological Sciences Research Council (BB/I025751/1 and BB/I025263/1). Additional epigenetic profiling on the ALSPAC cohort and the contributions of GCS and CLR were supported by the UK Medical Research Council Integrative Epidemiology Unit and the University of Bristol (MC_UU_12013_1, MC_UU_12013_2, MC_UU_12013_5 and MC_UU_12013_8). GCS is supported by two grants from the Medical Research Council: MR/S009310/1 and MR/S036520/1. The funders had no role in study design, data collection and analysis, decision to publish, or preparation of the manuscript.

## BAMSE

BAMSE was supported by The Swedish Research Council, The Swedish Heart-Lung Foundation, MeDALL (Mechanisms of the Development of ALLergy) a collaborative project conducted within the European Union (grant agreement No. 261357), Centre for Allergy Research, Stockholm County Council (ALF), Swedish foundation for strategic research (SSF) (RBc08-0027), the Strategic Research Programme (SFO) in Epidemiology at Karolinska Institutet and the Swedish Research Council Formas. EM is supported by grants from the Swedish Research Council, the Strategic Research Area Epidemiology at Karolinska Institutet, the European Research Council under the European Union (EU) Horizon 2020 (H2020) research and innovation programme (grant agreement number 757919, TRIBAL).

## CHAMACOS

CHAMACOS cohort research was supported by grants from the National Institute of Environmental Health Science (NIEHS) [P01 ESO09605, 5UG30D023356, R01ES012503, R01ES021369, R01ES023067, R24ES0285529]; Environmental Protection Agency (RD83273401, RD83171001), and the JPB Foundation. Its contents are solely the responsibility of the authors and do not necessarily represent the official views of NIEHS, EPA, or JPB Foundation.

**CHOP**

The research of the CHOP study reported herein was partially supported by the Commission of the European Community, specific RTD Programme “Quality of Life and Management of Living Resources,” within the 5th Framework Programme (research grant nos. QLRT-2001-00389 and QLK1-CT-2002-30582); the 6th Framework Programme contract no. 007036 (FP6-007036); the European Union’s Seventh Framework Programme Project EarlyNutrition under grant agreement no. 289346 (FP7-289346), the Horizon 2020 research and innovation programme DYNAHEALTH (no. 633595) and the European Research Council Advanced Grant META-GROWTH (ERC-2012-AdG – no. 322605). Additional support from the German Ministry of Education and Research, Berlin (Grant Nr. 01 GI 0825) and the University of Munich Innovative Research Priority Project MC-Health is gratefully acknowledged. This manuscript does not necessarily reflect the views of the Commission and in no way anticipates the future policy in this area. The funders of this study had no role in study design, data collection, data analysis, data interpretation, decision to publish, or preparation of the manuscript.

## CHS

This work was supported by NIEHS grants K01ES017801, R01ES022216, and P30ES007048.

**DOMInO**

The DOMInO Study and 3 and 5 year follow-up of the DOMInO children were supported by grants (349301, 570109) and Fellowships (APP1004211, APP1046207, APP1061074 and APP1052388) from the National Health and Medical Research Council of Australia (NHMRC). The epigenetic analyses of DOMInO samples were supported by the Science and Industry Endowment Fund (RP03-064), Diabetes Australia Research Trust and National Institutes of Health grant R35 CA 209859.

## GECKO

The GECKO Drenthe birth cohort was funded by an unrestricted grant of Hutchison Whampoa Ld, Hong Kong and supported by the University of Groningen, Well Baby Clinic Foundation Icare, Noordlease and Youth Health Care Drenthe. This methylation project in the GECKO Drenthe cohort was supported by the Biobanking and Biomolecular Research Infrastructure Netherlands (CP2011-19). This project received funding from the European Union’s Horizon 2020 research and innovation programme (733206, LIFECYCLE).

## The Generation R Study

The general design of the Generation R Study is made possible by financial support from the Erasmus Medical Center, Rotterdam, the Erasmus University Rotterdam, the Netherlands Organization for Health Research and Development and the Ministry of Health, Welfare and Sport.  The EWAS data was funded by a grant to VWJ from the Netherlands Genomics Initiative (NGI)/Netherlands Organisation for Scientific Research (NWO) Netherlands Consortium for Healthy Aging (NCHA; project nr. 050-060-810), by funds from the Genetic Laboratory of the Department of Internal Medicine, Erasmus MC, and by a grant from the National Institute of Child and Human Development (R01HD068437). VWJ received an additional grant from the Netherlands Organization for Health Research and Development (VIDI 016.136.361) and a Consolidator Grant from the European Research Council (ERC-2014-CoG-648916). This project received funding from the European Union’s Horizon 2020 research and innovation programme (633595, DynaHEALTH: 733206, LIFECYCLE) and from the European Joint Programming Initiative “A Healthy Diet for a Healthy Life” (JPI HDHL, NutriPROGRAM project, ZonMw the Netherlands no.529051022; and PREcisE project, ZonMw the Netherlands no.529051023).”

## GOYA

The Danish National Birth Cohort was established with a significant grant from the Danish National Research Foundation. Additional support was obtained from the Danish Regional Committees, the Pharmacy Foundation, the Egmont Foundation, the March of Dimes Birth Defects Foundation, the Health Foundation and other minor grants. The DNBC Biobank has been supported by the Novo Nordisk Foundation and the Lundbeck Foundation. Generation of DNA methylation data was funded by the MRC Integrative Epidemiology Unit which is supported by the Medical Research Council (MC_UU_12013/1-9) and the University of Bristol.

## Healthy Start

The Healthy Start study is funded by grants from the National Institute of Diabetes and Digestive and Kidney diseases (R01DK076648) and the National Institutes of Health, Office of the Director, Environmental Influences on Child Health Outcomes (ECHO) Program (UH3OD023248). The DNA methylation analysis in Healthy Start was funded by a grant from the National Institute of Environmental Health Sciences (R01ES022934). APS was funded by a grant from the National Institute of Environmental Health Sciences (R00ES025817).

**HELIX**

The study has received funding from the European Community’s Seventh Framework Programme (FP7/2007-206) under grant agreement no 308333—the HELIX project. INMA data collections were supported by grants from the Instituto de Salud Carlos III, CIBERESP, and the Generalitat de Catalunya-CIRIT. KANC was funded by the grant of the Lithuanian Agency for Science Innovation and Technology (6-04-2014_31V-66). The Norwegian Mother, Father and Child Cohort Study (MoBa) is supported by the Norwegian Ministry of Health and the Ministry of Education and Research, NIH/NIEHS (contract no. N01-ES-75558), and NIH/NINDS (grant no. 1 UO1 NS 047537-01 and grant no. 2 UO1 NS 047537-06A1). The Rhea project was financially supported by European projects, and the Greek Ministry of Health (Program of Prevention of Obesity and Neurodevelopmental Disorders in Preschool Children, in Heraklion district, Crete, Greece: 2011–2014; 'Rhea Plus': Primary Prevention Program of Environmental Risk Factors for Reproductive Health, and Child Health: 2012–2015). The work was also supported by MICINN (MTM2015-68140-R) and Centro Nacional de Genotipado-CEGEN-PRB2-ISCIII.

## INMA

This study was funded by grants from Instituto de Salud Carlos III (Red INMA G03/176), Generalitat de Catalunya-CIRIT 1999SGR 00241, and EU Commission (261357). This project received funding from the European Union’s Horizon 2020 research and innovation programme (733206, LIFECYCLE). LAS was supported through a Colciencias PhD Scholarship, Colombia (Grant: 529/2011). CRA received funding from the Catalan Government (#016FI_B 00272) and the Ministerio de Ciencia, Innovación y Universidades (RTI2018-100789-B-I00 (MCIU/AEI/FEDER, UE)).

## IOW F1

The IoW 1989 (IOW F1) cohort was supported by the National Institute of Allergy and Infectious Diseases under award numbers R01 AI091905 (PI: Wilfried Karmaus), R01 AI061471 (PI: Susan Ewart), and R01 AI121226 (PIs: Zhang, Holloway). The 18-year follow-up by a grant from the National Heart and Blood Institute (R01 HL082925, PI: S. Hasan Arshad).

## IOW F2

The IoW 1989 (IOW F2) cohort was supported by the National Heart, Lung, and Blood Institute under the award number R01 HL132321 (PI: Wilfried Karmaus).

## MoBa1, MoBa2

The Norwegian Mother, Father and Child Cohort Study is supported by the Norwegian Ministry of Health and Care Services and the Ministry of Education and Research, NIH/NIEHS (contract no N01-ES-75558), NIH/NINDS (grant no.1 UO1 NS 047537-01 and grant no.2 UO1 NS 047537-06A1). For this work, MoBa 1 and 2 were supported by the Intramural Research Program of the NIH, National Institute of Environmental Health Sciences (Z01-ES-49019) and the Norwegian Research Council/BIOBANK (grant no 221097). This work was partly supported by the Research Council of Norway through its Centres of Excellence funding scheme, project number 262700. WeNy is funded by the Norwegian Institute of Public Health.

## NEST

The NEST study was funded by NIEHS grants R21ES014947 and R01ES016772 and NIDDK grant R01DK085173. CH and DDJ received support from the Center for Human Health and the Environment grant received from the National Institute of Health Science (P30 ES025128). CH, SKM, and RLM also received support from a National Institute of Health Science grant (R24 ES028531).

## NFBC1986

NFBC1986 has received financial support from the Academy of Finland (104781, 120315, 121620, 129269, 1114194, 24300796), Center of Excellence in Complex Disease Genetics and SALVE, Oulu University Hospital, Oulu, Finland, Biocenter Oulu, Finland, University of Oulu, Finland (75617, 24002054, 2400692), Ministry of Social Affairs and Health (50459, 50691, 50842, 2749, 2465), NHLBI grant 5R01HL087679-02 through the STAMPEED program (1RL1MH083268-01), NIH/NIMH (5R01MH63706:02), ENGAGE project and grant agreement HEALTH-F4-2007-(201413), EU FP7 EurHEALTHAgeing (277849), EU FP7 EurHealth Epi-Migrant (279143), European Regional Development Fund 537/2010 (24300936) and the Medical Research Council, UK (G0500539, G0600705, G1002319, PrevMetSyn/SALVE).

Epigenetics research in the NFBC1986 received support by H2020–633595 DynaHEALTH, H2020 733206 LifeCycle, H2020-824989 EUCANCONNECT, the academy of Finland EGEA-project (285547), the Biocenter Oulu and the JPI HDHL, PREcisE project, ZonMw the Netherlands no. P75416).

## PIAMA

The PIAMA study is supported by The Netherlands Organization for Health Research and Development; The Netherlands Organization for Scientific Research; The Lung Foundation of the Netherlands (grant number AF 45.1.14.001 supported the methylation assays); The Netherlands Ministry of Spatial Planning, Housing, and the Environment; and The Netherlands Ministry of Health, Welfare, and Sport.

## PREDO

The Academy of Finland, EraNet, EVO (a special state subsidy for health science research), University of Helsinki Research Funds, the Signe and Ane Gyllenberg foundation, the Emil Aaltonen Foundation, the Finnish Medical Foundation, the Jane and Aatos Erkko Foundation, the Novo Nordisk Foundation, the Päivikki and Sakari Sohlberg Foundation, the Sigrid Juselius Foundation. KR acknowledges funding by the Academy of Finland (284859, 2848591, 312670); European Commission (Horizon 2020 Award SC1-2016-RTD-733280 RECAP); Signe and Ane Gyllenberg Foundation. This project received funding from the European Union’s Horizon 2020 research and innovation programme (733206, LIFECYCLE).

## Project Viva

This study was supported by grants from the National Institutes of Health (R01 NR013945, R01 HL111108, R01 HD 034568, UH3 OD023286). SL was supported by the National Institute of General Medical Sciences (grant T32GM074905). MFH has received an American Diabetes Association Pathways Accelerator Early Investigator Award (No 1-15-ACE-26)

## Raine

For Raine, the DNA methylation work was supported by NHMRC grant 1059711. Collaborative analyses are supported by NHMRC 1142858. Data collection and biological specimens at the 17-year follow-up were funded by the NHMRC Program Grant ID 353514 and Project Grant 403981. RCH and TAM are supported by NHMRC Fellowship grant number 1053384 and 1136046 respectively. This work was supported by resources provided by The Pawsey Supercomputing Centre with funding from the Australian Government and the Government of Western Australia**.**This project received funding from the European Union’s Horizon 2020 research and innovation programme (733206, LIFECYCLE).

## STOPPA

Financial support was provided by the Swedish Research Council (grant no 2018-02640) and through the Swedish Initiative for research on Microdata in the Social And Medical Sciences (SIMSAM) framework grant number 340-2013-5867, grants provided by the Stockholm County Council (ALF projects), the Swedish Heart Lung Foundation, the Swedish Asthma and Allergy Association's Research Foundation, FORTE and Stiftelsen Frimurare Barnahuset Stockholm. CA acknowledges support by the Swedish Research Council (grant number 2018-02640 and 340-2013-5867), grants provided by the Stockholm County Council (ALF projects), the Swedish Heart Lung Foundation, the Swedish Asthma and Allergy Association's Research Foundation, FORTE (grant number 2015-00289) and Stiftelsen Frimurare Barnahuset Stockholm. We acknowledge the Swedish Twin Registry for access to data. The Swedish Twin Registry is managed by Karolinska Institutet and receives funding through the Swedish Research Council under the grant no 2017-00641. We also acknowledge the Biobank at Karolinska Institutet for professional biobank service.

#

# Acknowledgements: study specific and in alphabetical order

## ALSPAC

We are extremely grateful to all the families who took part in this study, the midwives for their help in recruiting them, and the whole ALSPAC team, which includes interviewers, computer and laboratory technicians, clerical workers, research scientists, volunteers, managers, receptionists, and nurses. We would like to acknowledge Tom Gaunt, Oliver Lyttleton, Sue Ring, Nabila Kazmi, and Geoff Woodward for their earlier contribution to the generation of ARIES data (ALSPAC methylation data).

## BAMSE

We would like to thank all participating BAMSE children and parents for the engagement in our study. In addition, we would like to acknowledge Eva Hallner, Sara Nilsson and André Lauber at the BAMSE secretary for invaluable support, as well as Mutation Analysis Facility (MAF) at Karolinska Institutet for genome-wide methylation analysis, and Ingrid Delin for excellent technical assistance. The computations were performed on resources provided by SNIC through Uppsala Multidisciplinary Center for Advanced Computational Science (UPPMAX) under Project b2014110.

## CHAMACOS

We are grateful to the laboratory and field staff and participants of the CHAMACOS study for their contributions. We are thankful to Hong Quach who helped with 450K methylation analyses.

**CHOP**

We like to thank the participating families and all project partners for their enthusiastic support of the project work. We also like to thank Dr Eva Reischl and team at the Genome Analysis Center of Helmholtz Zentrum Muenchen, Germany for DNA extraction, bisulfite conversion and methylation analysis. We also like to acknowledge The European Childhood Obesity Trial Study Group for their continuous and salient support of the CHOP project: Philippe Goyens, Clotilde Carlier, Joana Hoyos, Pascale Poncelet, and Elena Dain (Universite Libre de Bruxelles – (ULB) –Brussels , Belgium); Jean-Noel Van Hees (CHC St Vincent– Françoise Martin, Annick Xhonneux, Jean-Paul Langhendries, and Jean-Noel Van Hees - Liège-Rocourt, Belgium); Ricardo Closa-Monasterolo, Joaquin Escribano, Veronica Luque, Georgina Mendez, Natalia Ferre, and Marta Zaragoza-Jordana (Universitat Rovira i Virgili, Institut d’Investigacio´ Sanitaria Pere Virgili, Taragona, Spain); Marcello Giovannini, Enrica Riva, Carlo Agostoni, Silvia Scaglioni, Elvira Verduci, Fiammetta Vecchi, and Alice Re Dionigi (University of Milano, Milano, Italy); Jerzy Socha, Piotr Socha and Anna Stolarczyk (Children’s Memorial Health Institute, Department of Gastroenterology, Hepatology and Immunology, Warsaw, Poland); Anna Dobrzanska and Dariusz Gruszfeld (Children’s Memorial Health Institute, Neonatal Intensive Care Unit, Warsaw, Poland); Roman Janas (Children’s Memorial Health Institute, Diagnostic Laboratory, Warsaw, Poland); Emmanuel Perrin (Danone Research Centre for Specialized Nutrition, Schiphol, the Netherlands); Rudiger von Kries (Division of Pediatric Epidemiology, Institute of Social Pediatrics and Adolescent Medicine, Ludwig Maximilians University of Munich, Munich, Germany); Helfried Groebe, Anna Reith, and Renate Hofmann (Klinikum Nurnberg Sued, Nurnberg, Germany); and Berthold Koletzko, Veit Grote, Martina Weber, Peter Rzehak, Sonia Schiess, Jeannette Beyer, Michaela Fritsch, Uschi Handel, Ingrid Pawellek, Sabine Verwied-Jorky, Iris Hannibal, Hans Demmelmair, Gudrun Haile, and Melissa Theurich (Division of Nutritional Medicine and Metabolism, Dr von Hauner Childrens Hospital, Ludwig-Maximilians Universität München (LMU), Munich, Germany).

## CHS

We are indebted to the school principals, teachers, students and parents in each of the study communities for their cooperation and especially to the members of the health testing field team for their efforts. We would like to express our sincere gratitude to Steve Graham and Robin Cooley at the California Biobank Program and Genetic Disease Screening Program within the California Department of Public Health for their assistance and advice regarding newborn bloodspots. The biospecimens and/or data used in this study were obtained from the California Biobank Program, (SIS request number(s) 479)” Section 6555(b), 17 CCR.  The California Department of Public Health is not responsible for the results or conclusions drawn by the authors of this publication.

## DOMInO

The authors acknowledge the DOMInO Steering committee for their support of this study (M Makrides, RA Gibson, AJ McPhee, LN Yelland, J Quinlivan, K Best, P Ryan). We would like to thank the families who participated; the medical, nursing, and research staff in each participating center and the whole DOMInO team, including the staff of the Child Nutrition Research Centre; and the staff of the Data Management and Analysis Centre, University of Adelaide, Adelaide, Australia. We gratefully acknowledge Susan van Dijk for her role in co-ordinating the DNA methylation analyses on the participants.

## GECKO

We are grateful to the families who took part in the GECKO Drenthe birth cohort, the midwives, gyneacologists, nurses and GPs for their help for recruitment and measurement of participants, and the whole team from the GECKO Drenthe study.

## The Generation R Study

The Generation R Study is conducted by the Erasmus Medical Center in close collaboration with the School of Law and Faculty of Social Sciences of the Erasmus University Rotterdam, the Municipal Health Service Rotterdam area, Rotterdam, the Rotterdam Homecare Foundation, Rotterdam and the Stichting Trombosedienst & Artsenlaboratorium Rijnmond (STAR-MDC), Rotterdam. We gratefully acknowledge the contribution of children and parents, general practitioners, hospitals, midwives and pharmacies in Rotterdam. The study protocol was approved by the Medical Ethical Committee of the Erasmus Medical Centre, Rotterdam. Written informed consent was obtained for all participants. The generation and management of the Illumina 450K methylation array data (EWAS data) for the Generation R Study was executed by the Human Genotyping Facility of the Genetic Laboratory of the Department of Internal Medicine, Erasmus MC, the Netherlands. We thank Mr. Michael Verbiest, Ms. Mila Jhamai, Ms. Sarah Higgins, Mr. Marijn Verkerk and Dr. Lisette Stolk for their help in creating the EWAS database. We thank Dr. A.Teumer for his work on the quality control and normalization scripts.

## GOYA

The authors want to thank the many pregnant women who have taken part in the study. Without them, there would be no cohort.

## Healthy Start

We would like to acknowledge the contributions of the study coordinator, Ms. Mercedes Martinez, MPH, the study staff, and the participating families for their continued involvement in the Healthy Start study.

**HELIX**

We would like to thank all the children and their families for their generous contribution.

## INMA

The authors would particularly like to thank all the participants for their generous collaboration. A full roster of the INMA Project Investigators can be found at http://www.proyectoinma.org/presentacion-inma/listado-investigadores/en_listado-investigadores.html.

## IOW F1 and IOW F2

We would like to thank all the participants of the Isle of Wight birth cohorts, the research team at David Hide Asthma & Allergy Research Centre (Isle of Wight) for collecting the data. In particular, the nurses for their help in recruiting them, Stephen Porter, Sharon Matthews, Frances Mitchell, Nikki Graham for technical support and other members of the IoW research group for valuable discussion. DNA methylation data was generated by the Oxford Genomics Centre at the Wellcome Trust Centre for Human Genetics.

## MoBa1, MoBa2

## The Norwegian Mother, Father and Child Cohort Study is supported by the Norwegian Ministry of Health and Care Services and the Ministry of Education and Research. We are grateful to all the participating families in Norway who take part in this on-going cohort study.

## NEST

We thank the parents and other caregivers of the Newborn Epigenetics STudy. We also thank the field and laboratory staff for their effort.

## NFBC1986

We gratefully acknowledge the contributions of the participants in the Northern Finland Birth Cohort 1986. We also thank all the field workers and laboratory personnel for their efforts.

## PIAMA

We thank all the children and their parents for their cooperation. We also thank all the field workers and laboratory personnel involved for their efforts, and Marjan Tewis for data management.

## PREDO

The PREDO study would not have been possible without the dedicated contribution of the PREDO Study group members: A Aitokallio-Tallberg, A-M Henry, VK Hiilesmaa, T Karipohja, R Meri, S Sainio, T Saisto, S Suomalainen-König, V-M Ulander, T Vaitilo (Department of Obstetrics and Gynaecology, University of Helsinki and Helsinki University Central Hospital, Helsinki, Finland), L Keski-Nisula (Kuopio University Hospital, Kuopio Finland), E Koistinen, T Walle, R Solja, P Taipale (Northern Karelia Central Hospital, Joensuu, Finland), M Kurkinen (Päijät-Häme Central Hospital, Lahti, Finland), P Staven (Iisalmi Hospital, Iisalmi, Finland), J Uotila (Tampere University Hospital, Tampere, Finland). We also thank the PREDO cohort mothers, fathers and children for their enthusiastic participation.

## Project Viva

We are grateful to families who participate in Project Viva.

## Raine

## The authors are grateful to The Raine Study participants and their families, and The Raine Study management team for cohort co-ordination and data collection, the National Health & Medical Research Council (NHMRC) for their long-term contribution to funding the study over the last 29 years and The Telethon Kids Institute for long term support of the Study. We also acknowledge The University of Western Australia (UWA), Raine Medical Research Foundation, The Telethon Kids Institute, Women and Infants Research Foundation, Edith Cowan University, Murdoch University, the University of Notre Dame Australia, Raine Medical Research Foundation, and Curtin University for providing funding for Core Management of The Raine Study.

## STOPPA

First, we direct our greatest appreciation to the twins and parents of the STOPPA cohort, without whose participation this study could not have been performed. We are also indebted to the STOPPA research nurses and database managers for their excellent data collection and data managing. We also want to direct our thanks to the eight paediatric allergy clinics around Sweden for their great collaboration during our visits for clinical examinations. We acknowledge the Swedish Twin Registry for access to data. The Swedish Twin Registry is managed by Karolinska Institutet and receives funding through the Swedish Research Council under the grant no 2017-00641. We also acknowledge the Biobank at Karolinska Institutet for professional biobank service.

**Supplementary Methods:** study specific and in alphabetical order

**Avon Longitudinal Study of Parents and Children (ALSPAC)**

***Design and study population***

The Avon Longitudinal Study of Parents and Children (ALSPAC) is a general population pregnancy cohort study that initially recruited 14 541 pregnancies with a due date between April 1991 and December 1992 in Avon, UK.^1, 2^ Written informed consent has been obtained for all ALSPAC participants. Ethical approval was granted from the ALSPAC Law and Ethics Committee and the local Research Ethics Committees. Please note that the study website contains details of all the data that is available through a fully searchable data dictionary ([https://www.bristol.ac.uk/alspac/researchers/our-data/](https://eur01.safelinks.protection.outlook.com/?url=https%3A%2F%2Fwww.bristol.ac.uk%2Falspac%2Fresearchers%2Four-data%2F&data=02%7C01%7Cf.vehmeijer%40erasmusmc.nl%7Cb50e3ef79dde441cda5508d77d8db807%7C526638ba6af34b0fa532a1a511f4ac80%7C0%7C0%7C637115919943943049&sdata=RuQe50GXemhgAGj6IV%2B%2FxIC89k5AtYLjnn0%2FSiDgH%2Bo%3D&reserved=0)).

Blood samples were collected from all consenting ALSPAC mothers and their offspring at several clinics at different time points. DNA methylation data for a sub-set of 1018 mother-offspring pairs was generated as part of the Accessible Resource for Integrated Epigenomics Studies (ARIES) project (http://www.ariesepigenomics.org.uk/). ARIES participants were selected based on availability of DNA samples at two time-points for the mother (antenatal and at follow-up when the offspring were mean age 17.1 years) and three time-points for the offspring (neonatal, childhood, mean age 7.5 and adolescence, mean age 17.1 years).

***Childhood and adolescent BMI***

Offspring height and weight were measured at regular clinics throughout childhood. BMI was calculated as weight (kg)/height (m^2^) and then age and sex adjusted using standard deviation scores as specified in the analysis plan. Measurements from the most recent clinic for each age range were used. If an individual did not attend that clinic their next most recent measurement was used.

***DNA methylation measurements***

Cord or peripheral blood (whole blood or buffy coats) were collected according to standard procedures, spun and frozen at -80˚C. DNA methylation analysis and data pre-processing were performed at the University of Bristol as part of the ARIES project (ariesepigenomics.org.uk). Following extraction, DNA was bisulfite converted using the Zymo EZ DNA MethylationTM kit (Zymo, Irvine, CA). Following conversion, the genome-wide methylation status of over 485,000 CpG sites was measured using the Illumina Infinium® HumanMethylation450k BeadChip assay according to the standard protocol. The arrays were scanned using an Illumina iScan and initial quality review was assessed using GenomeStudio (version 2011.1). The level of methylation is expressed as a “Beta” value (β-value), ranging from 0 (no cytosine methylation) to 1 (complete cytosine methylation). Samples from all time-points in ARIES were distributed across slides using a semi-random approach (sampling criteria were in place to ensure that all time-points were represented on each array) to minimize the possibility of confounding by batch effects. ISamples failing quality control (average probe detection p-value ≥ 0.01) were repeated. As an additional quality control step genotype probes on the HumanMethylation450k were compared between samples from the same individual and against SNP-chip data to identify and remove any sample mismatches. Data were pre-processed in R (version 3.0.1) with the WateRmelon package according to the subset quantile normalization approach described by Touleimat & Tost in an attempt to reduce the non-biological differences between probes.^3, 4^ We removed probes that had a detection P-value >0.05 for >5% of samples, probes on the X or Y chromosomes and SNPs (rs probes). For each probe, beta values were trimmed using the agreed IQR3 approach. The final dataset contained information on 471193 CpGs for 661 samples in cord blood, 472984 CpGs for 620 samples in childhood whole blood and 472416 CpGs for 667 samples in adolescent whole blood.

***Covariates***

Analyses were conducted on participants of European ancestry only. Birth weight (grams) was extracted from medical records. Maternal pre-pregnancy weight and height were self-reported then BMI was calculated as weight (kg)/height (m^2^). Gestational age was calculated (in days) based on the date of the mother’s last menstrual period (LMP) where the mother was certain of this, but for uncertain LMPs and conflicts with clinical assessment the ultrasound assessment was used. Where maternal report and ultrasound conflicted, an experienced obstetrician reviewed the clinical records and made a best estimate. Maternal age at delivery was derived from the mother’s report of her own and her baby’s dates of birth. Maternal social class was classified for this study as “attended university” or “did not attend university”. Parity was extracted from medical records and categorized for this study as nulliparous or parous; maternal smoking behaviour was assessed during pregnancy via questionnaire and categorised for this study as 1) never smoking during pregnancy, 2) any smoking during pregnancy. Breastfeeding was classified as “any breastfeeding” versus “no breastfeeding” based on maternal self-report. Smoking status during adolescence was based on self-report and classified as “never smoker” versus “ever smoker”. Participants were defined as being in late puberty if they were over 17 years old, and/or if they had information on Tanner stage that classified them as being in late puberty. We did not adjust for puberty stage in the adolescent analyses because the vast majority of participants were classified as late puberty. There were three participants classified (by Tanner) as early puberty, but these were excluded from our analyses.

***Cell type correction***

Cell type proportions were estimated using the estimateCellCounts function in the minfi R package, which is based on the method developed by Houseman.^5, 6^ This estimated the proportion of B-cells, CD8 T-cells, CD4 T-cells, granulocytes, NK-cells and monocytes in each sample.

***Batch correction***

During the data generation process a wide range of batch variables were recorded in a purpose-built laboratory information management system (LIMS). The LIMS also reported QC metrics from the standard control probes on the HumanMethylation450k BeadChip for each sample back to the laboratory. Of all measured batch variables, bisulfite conversion batch (96-well plate) was identified as by far the most influential on the ARIES HumanMethylation450k data. Slide level batch adjustment is less useful as each slide will only contain a small number of samples for each time point, additionally allocation to bisulfite conversion batch is more likely to contain systematic bias because samples were added to the batch according to lab priorities and convenience. However, running models with bisulfite conversion batch included as a factor variable (as is appropriate) caused non-singular fit errors (due to small batches), so we instead adjusted for batch by including in all models several surrogate variables generated using the sva() function in the SVA R package. These variables were generated separately for each exposure. 10 SVs were generated and then those that were associated with OM were discarded. The remainder (9 or 10 SVs depending on the model) were included in models as covariates.

The goal of SVA is to remove all unwanted sources of variation while protecting the contrasts due to traits and covariates of interest, so cell counts were included when generating SVs regardless of whether the EWAS was adjusted for cell counts. This way, the SVs will not be based on cell counts, so adjusting for cell counts should still have an effect.

## Children (Barn), Allergy, Milieu, Stockholm, Epidemiology (BAMSE)

***Design and study population***

BAMSE (Children, Allergy, Milieu, Stockholm, Epidemiology in Swedish) is a prospective population-based cohort study of children recruited at birth and followed during childhood and adolescence. Details of the study design, inclusion criteria, enrolment and data collection are described elsewhere.^7^ In short, 4,089 children born between 1994 and 1996 in four municipalities of Stockholm County were enrolled. At baseline, when the infant was approximately 2 months of age, parents completed a questionnaire that assessed residential characteristics, as well as socioeconomic and lifestyle factors, including parental smoking. When children were 1, 2, 4, 8, 12 and 16 years, the parents completed questionnaires focusing on children’s symptoms related to wheezing and allergic diseases, as well as various exposures. The survey response rates were 96%, 94%, 91%, 84%, 82% and 78%, respectively. Furthermore, blood was obtained from 2,614 (64%), 2,480 (61%) and 2549(62%) of the children at the age of 4, 8 and 16 years, respectively. The baseline and follow-up studies were approved by the Regional Ethical Review Board, Karolinska Institutet, Stockholm, Sweden, and the parents of all participating children provided written informed consent.

***Childhood and adolescent BMI***

Offspring height and weight were measured during clinical follow-up at 8 and 16 years of age by trained nurses using standard protocols. BMI was calculated as weight (kg)/height (m^2^), and subsequently transformed into sex- and age-adjusted standard deviation scores (SDS) using excel-macro LMSgrowth according to the analysis plan. Additionally, normal weight, overweight and obesity were defined using the Cole IOTF-standard.^8^

***DNA methylation measurements***

Epigenome-wide DNA methylation was measured in 472 Caucasian children at 8 years and 269 Caucasian children at 16 years of age. An aliquot (500 ng) of DNA per sample underwent bisulfite conversion using the EZ-96 DNA Methylation kit (Zymo Research Corporation, Irvine, USA). Samples were plated onto 96-well plates in randomized order. Samples were processed with the Illumina Infinium HumanMethylation450 BeadChip (Illumina Inc., San Diego, USA). A series of steps were completed for quality control and data analysis. First, we implemented sample filtering to remove bad quality and mixed up samples. Samples were excluded in case of sample call rate <99% in 0.3% of the probes, colour balance >3, low staining efficiency, poor extension efficiency, poor hybridization performance, low stripping efficiency after extension and poor bisulfite conversion. We also applied multidimensional scaling (MDS) plot to evaluate gender outliers based on chromosome X data, that produced two separated clusters for male and female. Furthermore, we applied median intensity plot for methylated and unmethylated intensity by using the minfi R package. All above leads to exclusion of 8 and 2 samples from 8- and 16-year data, respectively. Probes with a single nucleotide polymorphism in the single base extension site with a frequency of >5% were excluded^9^, as were probes with non-optimal binding (non-mapping or mapping multiple times to either the normal or the bisulphite-converted genome), and the probed belonging to chr X and chr Y, resulting in the exclusion of 46,799 and 47,654 probes (8- and 16-year DNA), leaving a total of 438,713 and 437,858 probes, respectively, in the analyses. Furthermore, we implemented “DASEN” recommended from wateRmelon package to do signal correction and normalization.^4^

***Covariates***

We used Medical Birth Registry to retrieve information on gestational age (in weeks), birthweight (in grams), parity (categorized for this study as nulliparous or parous), as well as maternal early pregnancy weight and height (BMI was calculated as weight (kg)/height (m^2^)). Maternal age at delivery, maternal educational level (classified for this study as 1) university or college degree or 2) 2-4 year secondary school or 3) 9-year compulsory school), maternal smoking during pregnancy (categorised for this study as 1) never smoking during pregnancy or 2) any smoking during pregnancy), breastfeeding (classified as 1) no breastfeeding; 2) any breastfeeding), and child´s sex were assessed via baseline questionnaire.

Additional covariates for the analyses of child BMI (measured at 8 years) included child´s age (in years), asthma ever up to the age of 8 years (selection variable), as well as concurrent environmental tobacco smoke exposure (classified as 1) no one smoking at child´s home; 2) anyone smoking in child´s home) were defined based on questionnaire reports at 8 year follow-up.

Additional covariates for the analyses of adolescent BMI (measured at 16 years) included adolescent´s age, current active smoking (categorised for this study as 1) no smoking or 2) any smoking), as well as puberty stage based on Petersen criteria (classified as 1 early puberty; 2) late puberty) was obtained from questionnaire reports at 16 year follow-up.^10^

***Cell type correction***

Cell type proportions were estimated using the estimateCellCounts function in the minfi R package based on the algorithm developed by Houseman ^5, 6^ Estimated blood cell subpopulations (CD8+ T-lymphocytes, CD4+ T-lymphocytes, natural killer cells, B-lymphocytes, monocytes, and granulocytes) were subsequently included as linear predictors in regression models.

***Batch correction***

The bisulfite treatment date was adjusted for as the batch correction for the 8-year methylation data, while for the 16-year data we used ComBat for batch correction.

## The Center for the Health Assessment of Mothers and Children of Salinas study (CHAMACOS)

***Design and study population***

The Center for the Health Assessment of Mothers and Children of Salinas (CHAMACOS) study is a longitudinal birth cohort study of the effects of exposure to pesticides and environmental chemicals on the health and development of Mexican-American children living in the agricultural region of Salinas Valley, CA. Detailed description of the CHAMACOS cohort has previously been published.^11^ Briefly, 601 pregnant women were enrolled in 1999-2000 at community clinics and 527 liveborn singletons were born. Follow up visits occurred at regular intervals throughout childhood. Study protocols were approved by the University of California, Berkeley Committee for Protection of Human Subjects and written informed consent was obtained from all mothers; oral assent was obtained from children beginning at age 7, and written assent at age 12.

***Childhood BMI***

Child height and weight were measured at each child visit to CHAMACOS field office in Salinas. Children were weight without shoes or jackets using an electronic scale (Tanita 1582, Arlington Heights, Illinois) at ages 5 and 7 and a foot-to-foot bioimpedence scale (Tanita TBF 300A, Arlington Heights, Illinois) at older ages. BMI was calculated as weight/height^2^ (kg/m^2^).

***DNA methylation measurements***

DNA methylation was measured in DNA isolated from the cord blood collected from CHAMACOS newborns and from blood samples collected from CHAMACOS 9 year olds by Illumina Infinium HumanMethylation450 (450K) BeadChips. DNA samples were bisulfite converted using Zymo Bisulfite Conversion Kits (Zymo Research, Irvine, CA), whole genome amplified, enzymatically fragmented, purified, and applied to the 450K BeadChips (Illumina, San Diego, CA) according to manufacturer protocol. 450K BeadChips were handled by robotics and analyzed using the Illumina Hi-Scan system. DNA methylation was measured at 485,512 CpG sites. Probe signal intensities were extracted by Illumina GenomeStudio software (version XXV2011.1, Methylation Module 1.9) methylation module and background subtracted. QA/QC was performed systematically by assessment of assay repeatability batch effects using 38 technical replicates, and data quality established as previously described.^12^ Quality was also ensured by only retaining samples where 95 % of sites assayed had detection P> 0.01. The same threshold (95% detection at p>0.01) was imposed to CpGs as well (n= 460 removed). Sites with annotated probe SNPs and with common SNPs (minor allele frequency >5%) within 50bp of the target identified in the MXL (Mexican ancestry in Los Angeles, California) HapMap population were excluded from analysis (n=49,748). This left a total of 435,369 CpGs in the analysis. Color channel bias, batch effects and difference in Infinium chemistry were minimized by application of ASMN algorithm, followed by BMIQ normalization.^12, 13^ The final dataset contained information on 435,369 CpGs for 378 cord blood and 200 9-year old blood samples.

***Covariates***

Data on birthweight (grams) and gestational age at birth (weeks) were extracted from medical records by a registered nurse. Maternal age, parity and education were assessed by participant interview at baseline visit (~13 weeks gestation). Maternal education was used as a proxy for maternal social class. Maternal education was treated as categorical, with two levels: less than a completed high school education, or having completed high school education or beyond. Maternal pre-pregnancy BMI was calculated using self-reported pre-pregnancy weight when interviewed at enrolment and measured height. Information on maternal smoking status was obtained through participant interview at baseline (~13 weeks gestation), follow up interview (~26 weeks gestation), and delivery.

***Cell type correction***

We used the Reinius-based Houseman method^6, 14^ with the estimateCellCounts function in the Minfi package^5^ in R^15^ to estimate relative proportions of six white blood cell subtypes (CD4+ T-lymphocytes, CD8+ T-lymphocytes, NK (natural killer) cells, B-lymphocytes, monocytes and granulocytes).

***Batch correction***

Analysis was also adjusted for batch effects by including 450K plate (n=10) as additional covariates.

**Childhood Obesity Project (CHOP)-Study**

***Design and study population***

The European Childhood Obesity Project (CHOP) study is an ongoing European multicenter randomized prospective nutritional intervention study in 1678 healthy term newborns recruited between October 1, 2002 and July 31, 2004. Currently, infants are followed up until the age of 11 years. Main objective of this study is to assess the effect of early and later nutrition on children’s weight development, growth, body composition and risk of obesity and the role epigenetic and metabolic programming plays in this context. A detailed description of the study design and the comprehensive prospective measurements can be found in recent publications.^16-20^ The local ethics committees of each study center approved all study procedures: Belgium (Comitè d’Ethique de L’Hopital Universitaire des Enfants Reine Fabiola; no. CEH 14/02), Germany (Bayerische Landesärztekammer Ethik-Kommission; no. 02070), Italy (Azienda Ospedaliera San Paolo Comitato Etico; no. 14/2002), Poland (Instytut Pomnik–Centrum Zdrowia Dziecka Komitet Etyczny; no 243/KE/2001), and Spain (Comité ético de investigación clinica del Hospital Universitario de Tarragona Joan XXIII). Written informed parental consent was obtained for each participating infant and from children of age 8 years onwards. For the EWAS of this paper a subset of 355 children of the CHOP study of age 5.5 years who had valid measurements on DNA-methylation data at that age and all required covariates was analysed.^16, 17, 19^

***Childhood BMI***

Child’s BMI is based on measured weight and height to the nearest millimeter and gram respectively by trained study personal during physical exams at 5.5 years using calibrated electronic balances and stadiometers. The calculation of the BMI_SDS and BMI_IOTF_Grade was done with the recommended excel-macro LMSgrowth and BMI_cat was computed with SAS (. combination of overweight and obese according to IOTF grades (coded 1) vs. normal weight (coded 0) and underweight (-1 where set to missing).

***DNA methylation measurements***

In the CHOP study Epigenome-wide DNA methylation was measured with the Illumina Infinium HumanMethylation450 BeadChip (Illumina Inc., San Diego, USA) as previously described.^19^ Briefly, genomic DNA was extracted from peripheral blood cells from buffy coats, bisulfite converted (800 ng) with the EZ-96 DNA Methylation Kit (Zymo Research, Irvine, Ca; USA) and finally hybridised on the Illumina Infinium HumanMethylation450 BeadChip arrays at the Genome Analysis Center of Helmholtz Zentrum Muenchen, Munich, Germany. Raw methylation data were pre-processed and normalized according to the approach of Touleimat and Tost with the modification of a beta-mixture quantile normalization (BMIQ) step.^3, 13^ Quality control was conducted according to standard criteria: Retaining only probes with signals from ≥3 beads, detection P-values≤0.01 and samples with ≥80% significant probe methylation signals per sample. In addition color bias correction and background adjustment were conducted with R-package lumi. However, except for identified cross-binding probes, no probe filtering according to proximity of CpG site with SNPs of minor allele frequency ≥5% within 50bp or probes on the X and Y chromosomes were conducted.^9^ In total, 431 313 CpG methylation values in for the 374 children of age 5.5 years were available for EWAS analysis before potential trimming of calculated beta-values. After trimming 429799 CpG methylation values were available for 355 samples.

***Covariates***

All covariates were selected from the CHOP server and recorded according to analysis plan. Data on maternal age, gestational age in weeks, birth weight of the child and parity were obtained from medical records during pregnancy or at birth. Information on mother’s highest educational level, maternal pre-pregnancy BMI and smoking status during pregnancy was assessed by questionnaire at the first study visit in the first weeks after birth, often in the week immediately after having given birth to the child. Status and length of pre-dominantly breastfeeding or formula feeding was regularly monitored during follow-up study visits during the first 12 months of the child’s life.^16, 17, 19^

***Cell type correction***

Cell type correction was applied using the reference-based Houseman method in the minfi Package in R.^5, 6, 21^ This method estimates the relative proportions of six white blood cell subtypes (CD4+ T-lymphocytes, CD8+ T-lymphocytes, NK (natural killer) cells, B-lymphocytes, monocytes and granulocytes), based on a standard reference population with the idat-files of the measured methylation data as the basic input.^14^

***Batch correction***

We corrected the analyses for batch effect by adjusting for plate number.

## Children’s Health Study (CHS)

***Design and study population***

The Children’s Health Study (CHS) is a population-based prospective cohort study from age 5 onwards in Southern California, which has been described in detail elsewhere.^22^ The study protocol was approved by the University of Southern California Institutional Review Board and informed, written consent and assent were provided by the parents and children respectively. A total of 5341 children were recruited, all of whom were born between 1995 and 1997 and are currently being followed until age 18.

***Childhood BMI***

Children enrolled in the CHS had height and weight measured by a trained technician at every study visit following a standardized protocol. Childhood BMI was measured as kg/m^2^ and converted to SDS using the method described in the analysis plan. We also used the method described in the analysis plan to categorize into underweight/normal/overweight/obese.

***DNA methylation measurements***

Based on the availability of newborn bloodspots archived by the state of California, a subset of 273 children was selected for a sub-study in which epigenome-wide DNA methylation was assessed in newborn bloodspots using the Infinium HumanMethylation450 BeadChip (HM450). Laboratory personnel performing DNA methylation analysis were blinded to study subject information. DNA was extracted from bloodspots using the QiaAmp DNA blood kit (Qiagen Inc, Valencia, CA) and stored at -80 degrees Celcius. 700-1000ng of genomic DNA from each sample was treated with bisulfite using the EZ-96 DNA Methylation Kit™ (Zymo Research, Irvine, CA, USA), according to the manufacturer’s recommended protocol and eluted in 18 ul. Chips were analyzed in three batches over a period of a couple of months. The results of the Infinium HumanMethylation450 BeadChip (HM450) were compiled for each locus as previously described and were reported as beta (β) values.^23^ A normal exponential background correction with dye bias correction was applied to the raw intensities at the array level to reduce background noise.^24^ We then normalized each sample’s methylation values to have the same quantiles to address sample to sample variability.^25^ CpG loci on the HM450 array were removed from analyses if they were on the X and Y chromosomes, or if they contained SNPs, deletions, repeats, or if they have more than 10% missing values, leaving 383,857 probes for analysis. Beta values were considered as outliers and were removed if they fall below Quartile 1-3×IQR or above Quartile 3+3×IQR. The final dataset contained information for 197 samples in cord blood after removing subjects with missing data for any of the covariates.

***Covariates***

Gestational age at birth, newborn gender and birth weight, maternal age at birth and maternal parity were obtained from California birth certificates. Data on maternal social class, maternal smoking status during pregnancy and child’s ethnic and racial background were obtained from parent-completed questionnaires at study entry when the subjects were around 6 years old. Ancestry was assessed from CHS genome-wide genotypic data using the program STRUCTURE from a set of ancestral informative markers that were scaled to represent the proportion of African American, Asian, Native American and white admixture.^26^

***Cell type correction***

Six white blood cell subtypes were estimated using the Reinius-based Houseman method ^6, 14^ Estimated cell subpopulations (CD8+ T-lymphocytes, CD4+ T-lymphocytes, natural killer cells, B-lymphocytes, monocytes and granulocytes) were subsequently included as linear predictors in regression models.

***Batch correction***

We additionally corrected the analyses for batch effect by including the Illumina Infinium HumanMethylation450 BeadChip plate number (n=3).

**DHA to Optimize Mother Infant Outcome (DOMInO) trial**

***Design and study population***

The DOMInO trial (ACTRN12605000569606) was a registered, multi-center, double-blind randomized controlled trial to investigate the impacts of DHA supplementation across the second half of pregnancy. DOMInO participants who had been enrolled at one of the Adelaide centres (Women’s and Children’s Hospital or Flinders Medical Centre; n=1660) and had not withdrawn or died were contacted when their child was 2.5 years of age and invited to participate in the growth and insulin resistance follow-up of the DOMInO children at 3 and 5 years of age (ACTRN12611001127998). Blood samples were collected from those children whose primary carer consented to this procedure. The blood samples were collected into EDTA blood tubes and placed on ice, most samples were processed within 4 hours and all samples within 24 hours of collection. DNA was extracted from peripheral blood leukocyte sample, using the Qiagen QIAquick DNA extraction kit.

***Childhood BMI***

At the 5 year appointments, between 25 March 2009 and 4 October 2013, the children’s height, weight, waist- and hip circumference were measured. BMI z-scores were calculated, using standardized reference charts for the child’s age and sex as described in the analysis plan.

***DNA methylation measurements***

DNA methylation was assessed using Illumina Infinium HumanMethylation450 BeadChips. Samples were submitted to the Australian Genome Research Facility (Parkville, VIC Australia) and processed following standard procedures. Data were pre-processed in R with normalization of the raw intensity data performed using a combination of NOOB (normal-exponential out-of-band), a background correction method with dye-bias normalisation^27^, followed by Subset-quantile Within Array Normalisation (SWAN) normalisation.^28^ Both methods are implemented in the Bioconductor package minfi.^5^ The BeadChip data was high quality. All arrays had a call rate > 99.3% per sample (mean of 99.9%); 1,256 probes had a detection P-value >0.05 for >5% of samples. Probes were not removed prior to meta-analysis. Instead, beta values were trimmed and set as missing using the agreed IQR3 approach. The final dataset (prior to IQR3 trimming) contained information on all 485,512 CpGs sites for 145 samples in peripheral blood.

***Covariates***

Data on maternal age in years, educational level (0 = did not complete high school; 1 = completed high school or other training; 2 = completed high school and further training), parity (nulliparous or parous) and smoking (1 = never smoking during pregnancy; 2 = any smoking during pregnancy) were collected by questionnaire on enrolment into the DOMInO trial. Gestational age was established using the last menstrual period or first trimester ultrasound measurement. Child sex and birthweight were obtained from medical records. Information on breastfeeding was obtained from postnatal questionnaires. DHA supplementation vs control was included and specified as binary variable. Ancestry was not included as a covariate as subjects were almost exclusively of European descent.

***Cell type correction***

The cell type correction was performed using the ‘estimateCellCounts’ function in minfi which is based on the Houseman method.

***Batch correction***

Samples were randomized across arrays and balanced according to sex, intervention group in the original trial, and date of birth. Array sentrix slide identifier was used (so batches are maximally 12 arrays in size). No row (or column) covariate was used. The combination of NOOB and SWAN performs well in removing the row batch effect, which is mostly caused by increased photobleaching of green (Cy3) dye in arrays on the slide scanned later in the scanning process (arrays in row 1 have the most photobleaching and row 6 the least).

## GECKO Drenthe

***Design and study population***

The Groningen Expert Center for Kids with Obesity (GECKO) Drenthe cohort is a population-based prospective birth cohort study in Drenthe, a northern province in the Netherlands. All mothers of infants born between April 2006 and April 2007 were invited to participate during the third trimester of pregnancy. Of all 4,778 infants born in this period, a total of 2,874 newborns (60%) participated in the study. This study has been approved by the Medical Ethical Committee of the University Medical Center Groningen and parents of all participants gave written informed consent. Details about this cohort have been described elsewhere.^29^

***Childhood BMI***

Height, weight and body mass index were measured repeatedly during childhood by staff at the community health centers according to a standard schedule and procedures. Height and weight were measured without shoes and heavy clothing.

***DNA methylation measurements***

The final dataset contained information on 465,891 CpGs for 137 samples in early childhood (~4y), and on 465,891 CpGs for 203 samples in later childhood (6y). Within the GECKO Drenthe birth cohort, we selected 258 infants for the methylation study: 129 exposed to maternal smoking during pregnancy and 129 unexposed to both maternal and paternal smoking during pregnancy ^30^ From these 258 infants, we used DNA which was extracted from cord blood for the epigenome-wide DNA methylation analyses. To limit batch effects, we randomized all samples over the 96-well plates, based on gender and smoking status. Samples (500 ng per sample) were placed on three 96-well plates. Bisulfite conversion was performed using the EZ-96 DNA methylation kit (Zymo research Corporation, Irvine, USA). Then we used the Infinium HumanMethylation450 BeadChip (Illumina Inc., San Diego, USA) to measure the methylation level as a beta value ranging from zero (no methylation) to one (complete methylation). During the quality control, we excluded two males that clustered in the female group, based on X chromosome betas, which was probably due to maternal blood contamination. We performed Illumina-suggested background normalization, colour correction and Subset-quantile Within Array Normalization (SWAN). We excluded one sample because it did not meet the criteria of ≥99% of the CpGs with detection p value <0.05. This resulted in 129 exposed and 126 unexposed children. We excluded control probes, probes on X or Y chromosomes and probes that did not meet our criteria of a detection p value of <0.05 in ≥99% of the samples, resulting in 465,891 remaining CpGs.

***Covariates***

Data on maternal age in years, height, weight, smoking, educational level and parity were collected by questionnaires in the third trimester of pregnancy. Maternal body mass index was calculated from height and weight. Gestational age, child sex and birthweight were documented by midwives, gynaecologists, nurses and GPs after birth. Information on breastfeeding was obtained from postnatal questionnaires.

***Cell type correction***

Cell type correction was applied using the reference-based Houseman method in the minfi Package in R.^5, 6, 21^ This method estimates the relative proportions of six white blood cell subtypes (CD4+ T-lymphocytes, CD8+ T-lymphocytes, NK (natural killer) cells, B-lymphocytes, monocytes and granulocytes), based on a standard reference population.^14^

***Batch correction***

We corrected the analyses for batch effect by adjusting for plate number.

## The Generation R Study

***Design and study population***

The Generation R Study is a prospective population-based cohort in Rotterdam, the Netherlands.^31^ All pregnant women residing in Rotterdam with a delivery date between April 2002 and January 2006 were invited to participate. The Medical Ethical Committee of Erasmus MC, University Medical Center Rotterdam, approved the study and an informed consent was obtained for all participating children. In total, 9,778 mothers were enrolled in the study.^31^

***Childhood BMI***

Height, weight and body mass index were measured repeatedly during childhood. Before the age of 4 years measurements were done by staff at the community health centers according to a standard schedule and procedures. Children around the age of 10 years were invited to visit our research center at the Erasmus MC-Sophia Children’s Hospital to participate in hands-on measurements.^31^ Height and weight were measured without shoes and heavy clothing.

***DNA methylation measurements***

DNA extracted (using the salting-out method) from cord blood and blood samples from children aged around 10 years of European-ancestry was used for this analysis. 500 ng DNA per sample underwent bisulfite conversion using the EZ-96 DNA Methylation kit (Shallow) (Zymo Research Corporation, Irvine, USA). Samples were plated onto 96-well plates in no specific order. Samples were processed with the Illumina Infinium HumanMethylation450 BeadChip (Illumina Inc., San Diego, USA), which analyses methylation at 485,577 CpGs. Quality control of analyzed samples was performed using standardized criteria, separately for the cord blood samples and the child samples, as the latter were added at a later stage.

***Quality control and normalisation of cord blood samples***

For the cord blood samples, reasons for sample exclusions were sample call rate <99%, colour balance >3, low staining efficiency, poor extension efficiency, poor hybridization performance, low stripping efficiency after extension, poor bisulfite conversion and gender mismatch. After quality control, 969 samples remained in the analysis. Probes with a single nucleotide polymorphism in the single base extension site with a frequency of >1% in the GoNLv4 reference panel were excluded, as were probes with non-optimal binding (non-mapping or mapping multiple times to either the normal or the bisulphite-converted Genome, resulting in the exclusion of 49,564 probes, leaving a total of 436,013 probes in the analysis.^32, 33^ We ran DASES normalization using a pipeline adapted from that developed by Touleimat and Tost.^3^ DASES normalization includes background adjustment, between-array normalization applied to type I and type II probes separately, and dye bias correction applied to type I and type II probes separately and is based on the DASEN method described by Pidsley *et al*. but adds the dye bias correction, which is not included in DASEN.^4^ The final dataset contained information on 436.013 CpGs for 789 (BMI in early childhood) and 687 (BMI in late childhood) samples in cord blood.

***Quality control and normalisation of child blood samples***

Preparation and normalization of the HumanMethylation450 BeadChip array data was performed according to the CPACOR workflow^1^ using the software package R^2^. In detail, the idat files were read using the minfi package. Probes that had a detection p-value above background (based on sum of methylated and unmethylated intensity values) ≥ 1E-16 were set to missing per array. Next, the intensity values were stratified by autosomal and non-autosomal probes and quantile normalized for each of the six probe type categories separately: type II red/green, type I methylated red/green and type I unmethylated red/green. Beta values were calculated as proportion of methylated intensity value on the sum of methylated+unmethylated+100 intensities. Arrays with observed technical problems such as failed bisulfite conversion, hybridization or extension, as well as arrays with a mismatch between sex of the proband and sex determined by the chr X and Y probe intensities were removed from subsequent analyses. Additionally, only arrays with a call rate > 95% per sample were processed further. The final dataset contained information on 457.574 CpGs for 358 samples in childhood.

***Covariates***

Data on maternal age in years, educational level and parity were collected by questionnaires in early pregnancy. Maternal body mass index was assessed at intake. Maternal smoking during pregnancy was assessed by questionnaires in each trimester of pregnancy. Gestational age was established using the last menstrual period or first trimester ultrasound measurement.^34^ Child sex and birthweight were obtained from midwife or hospital registries. Information on breastfeeding was obtained from postnatal questionnaires.

***Cell type correction***

Cell type correction was applied using the reference-based Houseman method in the minfi Package in R.^5, 6, 21^ This method estimates the relative proportions of six white blood cell subtypes (CD4+ T-lymphocytes, CD8+ T-lymphocytes, NK (natural killer) cells, B-lymphocytes, monocytes and granulocytes), based on a standard reference population.^14^

***Batch correction***

We corrected the analyses for batch effect by adjusting for plate number.

## Genome-Wide Population-based Association Study of Extremely Overweight Young Adults (GOYA)

***Design and study population***

The Genetics of Overweight Young Adults (GOYA) study is described in Paternoster et al.^35^ It includes a subset of 91,387 pregnant women recruited to the Danish National Birth Cohort during 1996–2002. Of 67,853 women who had given birth to a live born infant, had provided a blood sample during pregnancy and had BMI information available, 3.6% of these women with the largest residuals from the regression of BMI on age and parity (all entered as continuous variables) were selected for GOYA. The BMI for these 2451 women ranged from 32.6 to 64.4. From the remaining cohort a random sample of similar size (2450) was also selected. DNA methylation data were generated for the offspring of 1000 mothers in the GOYA study. I.e. “cases” had a BMI>32 and “controls” were sampled from the normal BMI distribution (can include women with a BMI>32). We restricted models to a randomly selected sub-group with a normal BMI distribution to avoid confounding by substructure.

***Childhood BMI***

Childhood BMI was measured as kg/m^2 and converted to SDS using the method described in the analysis plan. We also used the method described in the analysis plan to categorize into underweight/normal/overweight/obese.

***DNA methylation measurements***

Cord blood was collected according to standard procedures, spun and frozen at -80˚C. DNA methylation analysis and data pre-processing were performed at the University of Bristol. Following extraction, DNA was bisulfite converted using the Zymo EZ DNA MethylationTM kit (Zymo, Irvine, CA). Following conversion, the genome-wide methylation status of over 485,000 CpG sites was measured using the Illumina Infinium® HumanMethylation450k BeadChip assay according to the standard protocol. The arrays were scanned using an Illumina iScan and initial quality review was assessed using GenomeStudio (version 2011.1). The level of methylation is expressed as a “Beta” value (β-value), ranging from 0 (no cytosine methylation) to 1 (complete cytosine methylation). Samples from all time-points in ARIES were distributed across slides using a semi-random approach (sampling criteria were in place to ensure that all time-points were represented on each array) to minimize the possibility of confounding by batch effects. Samples failing quality control (average probe detection p-value ≥ 0.01) were repeated. As an additional quality control step genotype probes on the HumanMethylation450k were compared between samples from the same individual and against SNP-chip data to identify and remove any sample mismatches. Data were normalized using the functional normalization approach in the Minfi R package. We removed probes that had a detection P-value >0.05 for >5% of samples, probes on the X or Y chromosomes and SNPs (rs probes). We used the IQR3 trim. The final dataset contained information on 473864 CpGs for 539 samples in cord blood.

***Covariates***

Data on covariates were collected via a telephone interview at around 16 weeks gestation. Maternal age was derived from the mother’s report of her own date of birth. Socioeconomic status was defined using maternal education or occupation: 1) manager/long or medium education, 2) work requiring a short training period, or skilled manual labour, 3) unskilled or public service. Parity was categorized for this study as nulliparous or parous. Maternal smoking in pregnancy was defined as any smoking in pregnancy or no smoking in pregnancy.

***Cell type correction***

Cell type correction was applied using the reference-based Houseman method in the minfi Package in R.^5, 6, 21^ This method estimates the relative proportions of six white blood cell subtypes (CD4+ T-lymphocytes, CD8+ T-lymphocytes, NK (natural killer) cells, B-lymphocytes, monocytes and granulocytes), based on a standard reference population.^14^

***Batch correction***

Ten surrogate variables were generated and included in models to adjust for technical batch.

## Healthy Start

***Design and study population***

The Healthy Start study is an ongoing, prospective pre-birth cohort in Colorado, USA.^36, 37^ Eligible pregnant women were recruited from the University of Colorado Hospital Outpatient Pavilion, 2009-2014. Eligibility criteria were: age 16 years or older, singleton pregnancy, no prior stillbirths, and gestational age < 24 weeks. We recruited 1,410 pregnant women, approximately 50% of those eligible. Study procedures were approved by the Colorado Multiple Institutional Review Board and written informed consent was obtained from all participating mothers.

***Childhood BMI***

Child height and weight were measured by clinical personnel at routine medical visits and abstracted from records by study staff. Most children visit paediatricians at least annually for preventive care and immunizations. For children with multiple height and weight measurements within the desired age interval, the weight and height at the oldest age was used for analysis.

***DNA methylation measurements***

Umbilical cord blood was collected at delivery. Eligibility for DNA methylation analysis was determined by the availability of cord blood and maternal blood and urine samples during pregnancy. Methylation analysis of cord blood samples (N= 600) was conducted using the Illumina Infinium HumanMethylation450 BeadChip, and processed in the University of Colorado Genomics Core lab. Samples with predicted child sex inconsistent with reported sex were excluded. Probes with high detection p-value (>0.05) or low beadcount (<3 in at least 5% of the samples) were excluded. All analyses were conducted in R version 3.3.0*(8)*. The preprocessQuantile function in the R package Minfi was used for normalization.^5^ The total number of CpGs analysed was 484,261 in 87 samples.

***Covariates***

At study enrolment, pregnant women reported their age, race/ethnicity, education completed, and parity. Standing height was measured at the first research visit by trained study personnel. Weight prior to pregnancy was obtained from the medical record, if available, or by self-report. Women were asked twice during pregnancy and once at delivery to report current or recent smoking. Child sex, gestational age at birth, and birth weight were obtained from the delivery medical record.

***Cell type correction***

The Houseman method was used for estimation of 6 cell types in cord blood, using the estimateCellCounts function in minfi.

***Batch correction***

ComBat was used for batch correction.

**HELIX**

***Design and study population***

Human Early Life Exposome (HELIX) study represents a collaborative project across six established and ongoing longitudinal population-based birth cohort studies in Europe: the Born in Bradford (BiB) study in the UK, the Étude des Déterminants pré et postnatals du développement et de la santé de l’Enfant (EDEN) study in France, the INfancia y Medio Ambiente (INMA) cohort in Spain, the Kaunus cohort (KANC) in Lithuania, the Norwegian Mother, Father and Child Cohort Study (MoBa) and the RHEA Mother Child Cohort study in Crete, Greece. The HELIX project aims to measure and describe multiple environmental exposures from the different exposome domains during early life (pregnancy and childhood) and associate these with omics markers and child health outcomes.^38, 39^

The six HELIX cohorts have the required permissions by national ethics committees for their cohort recruitment and follow-up visits and for secondary use of pre-existing samples and data. The work in HELIX was covered by new ethics approvals in each country. At enrolment in the HELIX project, families were asked to sign an informed consent form for the specific HELIX work including clinical examination and biospecimen collection and analysis. An Ethics Task Force was established to support the HELIX project on ethical issues, for advice on the project’s ethical compliance, identification and alerting to changes in legislation where applicable. Specific procedures are in place within HELIX to safeguard the privacy of study subjects and confidentiality of data.^38^

***Childhood BMI***

Height and weight were measured following the same harmonized protocol in all the cohorts. BMI was calculated as weight (kg)/height (m^2^) and then age and sex adjusted using standard deviation scores as specified in the analysis plan.

***DNA methylation measurements***

DNA methylation data were pre-processed using the minfi package.^5^ Following guidelines of Lehne work^40^, we increased the stringency of the detection p-value threshold to 10E-16 and probes not reaching a 98% call rate were excluded. Two samples were filtered due to overall quality: one had a call rate <98% and the other did not pass QC parameters of the MethylAid package.^41^ Then, data was normalized with the functional normalization method, which also includes Noob background subtraction and dye-bias correction.^27^ After that, several quality control checks were performed. First, we checked sex consistency using the shinyMethyl package^42^ and two samples were excluded. Genetic consistency of duplicates and samples from the same participant was checked with the 450k genotypes. In addition, genetic consistency was evaluated in those samples that had GWAS data and two of them were excluded. Centered-correlation was around 0 for unrelated samples and around 0.8 for duplicates and panel samples. Principal component analysis showed no differential clusters, however a degree of grouping within the cluster was observed for some biological variables (sex, cohort) and for some technical variables. Duplicated samples and HapMap samples were removed as well as control probes, probes designed to detect SNPs and probes to measures methylation levels at non-CpG sites. The final dataset consisted of 1,347 HELIX samples representing 1,192 subjects and 480071 probes.

***Cohort adjustment***

Five different HELIX cohorts where used: BIB (United Kingdom), EDEN (France), KANC (Lithuania), MoBa (Norway) and RHEA (Greece). Models were adjusted for cohort.

***Covariates***

BiB: Maternal age was derived from date of birth obtained from hospital records and date of recruitment/date of child’s birth. Parity, mother’s booking weight, gestational age, child sex and birth weight were obtained from the hospital maternity IT system. Maternal smoking and educational level were obtained from the baseline questionnaire administered at recruitment. Maternal height was measured at baseline, and BMI calculated using the booking weight. Information on breast feeding was obtained from the 6 and 12 month BiB1000 questionnaires.

KANC: Maternal age was derived from date of birth obtained from hospital records and KANC cohort face-to-face questionnaire filled up during of recruitment. Child sex and birth weight were obtained through birth certificate using the hospital maternity IT system. Individual-level covariates were obtained through standardized interviews: parity, mother’s height/weight, smoking, and educational level; gestational age was established using ultrasound. Information on breast feeding was obtained from questionnaires.

EDEN: Information on mother’s pre-pregnancy weight, height, educa­tional level and maternal smoking were collected by interview between 24 and 28 weeks of gestation. Maternal age at delivery and parity were collected by ques­tionnaires during pregnancy and after delivery. Gestational age, child sex, birth weight and information on breast feeding were obtained from the hospital maternity records and questionnaires after birth.

RHEA: Maternal pre-pregnancy BMI was calculated from measured height and self-reported pre-pregnancy weight collected at enrolment (week 12 of pregnancy), maternal age was calculated from date of birth and date of delivery, parity was self-reported at enrolment, gestational age was based on US and/or date LMP, child sex and birth weight from birth records, maternal smoking reported by the mother at 12th and 30th week of pregnancy, education self-reported at enrolment, breastfeeding self-reported at 9 months postpartum and updated on later follow-ups.

MoBa: Maternal age at delivery, child’s sex and birthweight were obtained from the Medical Birth Registry of Norway (MBRN). Gestational age was calculated from expected date of delivery on the basis of first trimester ultrasound. If ultrasound measure was missing, gestational age was calculated from last menstrual period. Maternal height and pre-pregnancy weight were self-reported at week 17 in pregnancy and pre-pregnant BMI was calculated as kg/m^2^. Information on maternal smoking was reported by the mother at the start and end of pregnancy and obtained from MoBa questionnaire. Mother’s educational level was also obtained by MoBa questionnaire.

***Cell type correction***

Cell type proportions were estimated using the estimateCellCounts function in the minfi R package^5^, based on the method developed by Houseman, and the Reinius reference panel.^14^ This estimated the proportion of B-cells, CD8 T-cells, CD4 T-cells, granulocytes, NK-cells and monocytes in each sample.

***Batch correction***

We used COMBAT algorithm to adjust for potential batch effects, using slide as the major known technical bias.^43^

## Infancia y Medio Ambiente (INMA)

***Design and study population***

The INMA—INfancia y Medio Ambiente—(Environment and Childhood) Project is a network of birth cohorts in Spain that aim to study the role of environmental pollutants in air, water and diet during pregnancy and early childhood in relation to child growth and development.^44^ The study has been approved by Ethical Committee of each participating centre and written consent was obtained from participating parents. Data for this study came from INMA Sabadell cohort (children born between 2004 and 2007).

***Childhood BMI***

Child weight (nearest gram) and height (nearest 0.1cm) at the 4years and 7 years of age visits and they were measured by trained staff using standard protocols (without shoes and in light clothing).^45^

***DNA methylation measurements***

INMA 450k data was produced within the MEDALL and BREATHE projects, for this project we only used MEDALL generated data. DNA from peripheral and umbilical cord blood samples was extracted using the QIAamp blood kit (Qiagen), followed by a precipitation-based concentration using GlycoBlue (Ambion). DNA concentration was determined by Nanodrop measurement and picogreen quantification. 500 ng of DNA was bisulfite-converted using the EZ 96-DNA methylation kit following the manufacturer’s standard protocol, and DNA methylation measured using the Illumina Infinium HumanMethylation450 beadchip. DNA methylation data were preprocessed using the minfi package, for the original umbilical cord blood samples using DASEN and the functional normalization method from the minfi package was used for normalization for peripheral blood at 4 yrs of age.^4, 5^

A series of steps were completed for quality control and data analysis. First, we implemented sample filtering to remove bad quality and mixed up samples. Second, we filtered out the probes to remove the CpG sites which are not mapped to unique location on the genome and CpGs containing single nucleotide polymorphisms (SNPs) at the target site. Third, we implemented “DASEN” for the umbilical cord blood and functional normalization for the 4yrs bloods, to perform signal correction and normalization. The final dataset contained information from umbilical cord blood on 439,306 CpGs for 176 samples (with BMI information in early childhood around 4 yrs of age) and 168 samples (with BMI information in late childhood around 7 yrs of age); and information from peripheral blood at four years of age on 476,946 CpGs for 200 samples with information of BMI measured in early childhood (around four years of age).

***Covariates***

Infant sex was abstracted from clinical records. We calculated gestational age from the date of the last menstrual period (LMP) reported at recruitment and confirmed using estimates based on the first ultrasound examination (about 12th week of gestation). When the difference between the LMP reported at recruitment and estimated from the ultrasound was ≥ 7 days, we estimated LMP using the crown-rump length.^45^ Data on age at birth, and maternal height and pre-pregnancy weight (for BMI calculations) were collected from a self-reported using a questionnaire at enrolment (week 12 of pregnancy). Maternal education was categorized into three levels: low (primary or less), medium (secondary) or high (university or higher). Pregnant women were asked whether they were current smokers (at week 32 of pregnancy) and if so, how much. They were also asked if they had stopped smoking due to pregnancy and when (before pregnancy or at what month of pregnancy). Any smoking was defined as smoking any number of cigarettes at any time during pregnancy. For models at 4 yr of age, the child age at sample collection was included as a covariate. Birth weight was recorded by specially trained midwives at delivery. Breastfeeding information was obtained by interviewer‐administered questionnaires with mothers when the children were 6 months and 14 months of age.

***Cell type correction***

We used the *estimateCellCounts* function from minfi which estimates a constrained projection using quadratic programming^6^ we estimated six white blood cell subtypes (CD4+ T-lymphocytes, CD8+ T-lymphocytes, NK (natural killer) cells, B-lymphocytes, monocytes and granulocytes) using the Reinius reference.^14^

***Batch correction***

For umbilical cord blood, batch correction was attained including the significant (permutation p-value< 10-4) principal components derived from the 613 negative control probes presented in 450K arrays. After 10.000 permutations 5 PCs were retained. The beta-values were batch corrected incorporating these 5 PCs and calculating the residuals of the linear model. For the larger data (MEDALL and BREATHE) including both umbilical cord blood and peripheral blood at 4 yrs of age, we used ComBat for batch correction. ComBat is an algorithm that can correct batch effect artifacts using empirical Bayes frameworks.^43^ It was applied to the beta values transformed to M-values and without the NAs. To avoid infinities, beta values equal to 0 were transformed to 0.001 and those equal to 1 transformed to 0.999. The resulting matrix was transformed again to beta values and used in the subsequent results. ComBat was run inserting the variable age in the null model to protect age from batch correction. Only those samples of peripheral blood at 4 yrs were used for this analysis.

## Isle of Wight Birth Cohort (IOW F1)

***Design and study population***

A whole population birth cohort was established on the Isle of Wight, UK, in 1989 to prospectively study the natural history of allergic diseases from birth onwards.^46^ Both the Isle of Wight and the study population are 99% Caucasian. Ethics approvals were obtained from the Isle of Wight Local Research Ethics Committee (now named the National Research Ethics Service, NRES Committee South Central –Southampton B) at recruitment and for the 1, 2, 4, 10 and 18 years follow-up. Of the 1536 children born between January 1, 1989, and February 28, 1990, written informed consent was obtained from parents to enrol 1456 new-borns. Children were followed up at the ages of 1 (n = 1167), 2 (n = 1174), 4 (n = 1218), 10 (n = 1373), and 18 years (n = 1313). Demographic information of parents and offspring, status of allergic diseases, phenotypic measures on allergic sensitization, IgE, and lung function, and environmental exposures, along with other phenotypic measures, were collected at birth and updated at each follow-up.

***Childhood and adolescent BMI***

Child BMI was calculated from child’s height and weight at 10 and 18 years which is collected from the medical records and data collection via paper questionnaire was entered into two independent copies of an SPSS data sheet, the two sheets were compared and discrepancies resolved.

***DNA methylation measurements***

For 299 subjects at 10 years of age, we measured DNA methylation from whole blood processed with the Illumina Infinium MethylationEPIC Beadchip. For the adolescence model, DNA methylation of 367 aged 18 years subjects was measured from whole blood processed with the Illumina Infinium HumanMethylation450 Beadchip. CPACOR pipeline was used for QC and normalisation for both of the data.^40^ Methylation markers on 65 single nucleotide polymorphism (SNP) and sex chromosomes were removed. We applied Illumina background Correction to all intensity values. Any intensity values having detection p-values >= 10^-16^ were set as missing data. Samples with call rate < 98% were excluded. After the QC, 754,150 and 473,864 sites remain for the subsequent analysis for 10 and 18 years samples receptively. A quantile normalisation was applied using limma on intensity values separately based on six different probe-type categories (Type-I M red, Type-I U red, Type-I M green, Type-I U green, Type-II red, and Type-II green). Beta values were then calculated from these normalised intensity values. Outlier CpGs were removed using 3IQR method after which 562,834 sites for 286 samples remained for 10 years samples and 373,936 sites for 367 samples remained for 18 years samples. For complete phenotype datasets, 192 samples and 172 samples were used respectively for 10 and 18 years samples for further analysis.

***Covariates***

Covariates were collected via questionnaires collected at recruitment, before and during pregnancy. Maternal age was derived from mothers’ date of birth. In case of maternal BMI, we used BMI at 1^st^ trimester. Gestational age was checked and collected from the maternity notes. Breastfeeding data was collected by questionnaire at 1 and 2 year follow-ups. Maternal smoking status and parity were collected from the responses from the questionnaires. Maternal smoking status in pregnancy (Yes/No) was defined as any smoking in pregnancy or no smoking in pregnancy. Socioeconomic status was defined using maternal socioeconomic cluster information (high, low, low-low, low-mid, and mid). Birth weight was collected from the medical records and data collection via paper questionnaire. Samples smoking status was collected from the follow-up questionnaire. No puberty status was available.

***Cell type correction***

We used the Reinius-based Houseman method^6, 14^ with the estimateCellCounts function in the Minfi package^47^ to estimate relative proportions of six white blood cell subtypes (CD4+ T-lymphocytes, CD8+ T-lymphocytes, NK (natural killer) cells, B-lymphocytes, monocytes and granulocytes).

***Batch correction***

Indicator of different batches that DNA methylation data were generated were included as a covariate to adjust for batch effect and ComBat was used to remove any batch effect.^43^

## Isle of Wight Third Generation (IOW F2)

***Design and study population***

The recruitment of new-borns started from April 2010. Data used in the analyses were from infants born between April 2010 to May 2014.^46^ In total, 200 new-borns were recruited such that at least one of their parents is in the IOW birth cohort (IOW F1) and recruitment is ongoing. For each infant, along with other phenotypic information such as gender and birthweight, status of wheezing and eczema was recorded, measures of wheal size from skin prick test as well as IgE were recorded.

***Childhood BMI***

Child BMI was calculated from child’s height and weight at 2 – 3 years which is collected from the medical records and data collection via paper questionnaire was entered into two independent copies of an SPSS data sheet, the two sheets were compared and discrepancies resolved.

***DNA methylation measurements***

For 127 subjects, we measured DNA methylation from whole blood processed with the Illumina Infinium HumanMethylation450 Beadchip (Illumina Inc., San Diego, USA). CPACOR pipeline was used for QC and normalisation of the data. Methylation markers on 65 single nucleotide polymorphism (SNP) and sex chromosomes were removed. We applied Illumina background correction to all intensity values. Any intensity values having detection p-values >= 10^-16^ were set as missing data. Samples with call rate < 98% were excluded. After the QC, 473,864 sites remain for the subsequent analysis. A quantile normalisation was applied using limma on intensity values  separately based on six different probe-type categories (Type-I M red, Type-I U red, Type-I M green,  Type-I U green, Type-II red, and Type-II green). Beta values were then calculated from these normalised intensity values.  The final dataset contained information on 403,121 CpGs for 58 samples.

***Covariates***

Covariates were collected via questionnaires collected at recruitment, before and during pregnancy. Maternal age was derived from mothers’ date of birth. Child’s gender and gestational age at birth were collected from stored clinical information. In case of maternal BMI, we used BMI at 1st trimester. Maternal smoking status and parity were collected from the responses from the questionnaires. Maternal smoking status in pregnancy (Yes/No) was defined as any smoking in pregnancy or no smoking in pregnancy. Socioeconomic status was defined using maternal education Birth weight was collected from stored clinical records. The outliers for observe birth weight values +/- 5 SD from the mean was checked.

***Cell type correction***

We used the Reinius-based Houseman method^6, 14^ with the estimateCellCounts function in the Minfi package^47^ to estimate relative proportions of six white blood cell subtypes (CD4+ T-lymphocytes, CD8+ T-lymphocytes, NK (natural killer) cells, B-lymphocytes, monocytes and granulocytes).

***Batch correction***

Batch was used as a separate covariate in analyses.

## Norwegian Mother, Father and Child Cohort Study 1 & 2 (MoBa1 and MoBa2)

***Design and study population***

Participants represent three subsets of mother-offspring pairs from the national Norwegian Mother, Father and Child Cohort Study (MoBa).^48^ MoBa is a prospective population-based pregnancy cohort study conducted by the Norwegian Institute of Public Health. The years of birth for MoBa participants ranged from 1999-2009. MoBa mothers provided written informed consent. Each subset is referred to here as MoBa1 and MoBa2. MoBa1 is a subset of a larger study within MoBa that included a cohort random sample and cases of asthma at age three years.^49^ We previously reported an association between maternal smoking during pregnancy and differential DNA methylation in MoBa1 newborns.^50^ We subsequently measured DNA methylation in additional newborns (MoBa2) in the same laboratory (Illumina, San Diego, CA)^51^ MoBa2 included cohort random samples plus cases of asthma at age seven years and non-asthmatic controls. Years of birth were 2002-2004 for children in MoBa1 and between 2000-2005 for MoBa2. The establishment and data collection in MoBa has obtained a license from the Norwegian Data Inspectorate and approval from The Regional Committee for Medical Research Ethics. All three studies were approved by the Regional Committee for Ethics in Medical Research, Norway. In addition, MoBa1 and MoBa2 were approved by the Institutional Review Board of the National Institute of Environmental Health Sciences, USA.

The consent given by the participants does not allow for storage of data on an individual level in repositories or journals. Researchers who want access to data sets for replication should submit an application to [datatilgang@fhi.no](mailto:datatilgang@fhi.no). Access to data sets requires approval from The Regional Committee for Medical Research Ethics in Norway and a formal contract with MoBa.

***Childhood BMI***

Childhood BMI was estimated from maternal reports at 3 years and at 7 years.

***DNA methylation measurements***

Details of the DNA methylation measurements and quality control for the MoBa1 participants were previously described and the same protocol was implemented for the MoBa2 participants.^50^ Briefly, umbilical cord blood samples were collected and frozen at birth at -80°C. All biological material was obtained from the Biobank of the MoBa study.^52^ Bisulfite conversion was performed using the EZ-96 DNA Methylation kit (Zymo Research Corporation, Irvine, CA) and DNA methylation was measured at 485,577 CpGs in cord blood using Illumina’s Infinium HumanMethylation450 BeadChip. Raw intensity (.idat) files were handled in R using the minfi package to calculate the methylation level at each CpG as the beta-value (β=intensity of the methylated allele (M)/(intensity of the unmethylated allele (U) + intensity of the methylated allele (M) + 100)) and the data was exported for quality control and processing. Probe and sample-specific quality control was performed in the MoBa1 and MoBa2 datasets separately. Similar protocols were applied to MoBa1 and Moba2, as follows: Control probes (N=65) and probes on X (N=11 230) and Y (N=416) chromosomes were excluded in both datasets. Remaining CpGs missing > 10% of methylation data were also removed (N=20 in MoBa1, none in MoBa2). Samples indicated by Illumina to have failed or have an average detection p value across all probes < 0.05 (N=49 MoBa1, N=35 MoBa2) and samples with gender mismatch (N=13 MoBa1, N=8 MoBa2) were also removed. For MoBa1 and MoBa2, we accounted for the two different probe designs by applying the intra-array normalization strategy Beta Mixture Quantile dilation (BMIQ). After quality control exclusions, the sample sizes were 1,068 for MoBa1 and 685 for MoBa2.

***Covariates***

For both datasets, information on gestational age, child’s sex, maternal age, smoking during pregnancy, education, pre-pregnancy BMI, and parity was collected via questionnaires completed by the mother or from birth registry records as previously described (4). Gestational age, maternal age, and pre-pregnancy BMI were included as continuous variables. Child’s sex and parity were included as dichotomous variables. Maternal smoking status during pregnancy was classified into three groups: non-smoker, stopped smoking in early pregnancy, and smoked throughout pregnancy. Maternal educational level was categorized into four groups based on years of education: less than high school/secondary school, high school/secondary school completion, some college or university, or 4 years of college/university or more. Each dataset was analysed independently.

***Cell type correction***

We used the Houseman method with the estimateCellCounts function in the Minfi package^5^ in R^15^ to estimate relative proportions of six white blood cell subtypes (CD4+ T-lymphocytes, CD8+ T-lymphocytes, NK (natural killer) cells, B-lymphocytes, monocytes and granulocytes).

***Batch correction***

The Empirical Bayes method via ComBat was applied separately in MoBa1 and MoBa2 for batch correction using the sva package in R.

## The Newborn Epigenetics Study (NEST)

***Design and study population***

The Newborn Epigenetics STudy (NEST) is a multi-ethnic birth cohort designed to identify the effects of early exposures on epigenetic profiles and phenotypic outcomes. Pregnant women were recruited from prenatal clinics serving Duke University Hospital and Durham Regional Hospital Obstetrics facilities in Durham, North Carolina from April 2005 to July 2009. Gestational age at enrollment ranged from 6 to 42 weeks (median 30 weeks). Eligibility criteria were women aged 18 years or older, English speaking, pregnant, and an intention to use one of the two obstetrics facilities. Among these, women infected with HIV or intending to give up custody of the offspring of index pregnancy were excluded. Current smokers were targeted for the first ~200 participants. Of the 1101 women who met eligibility criteria and were approached, 895 (81%) were enrolled and umbilical cord blood was collected from 741 infants. The current analysis was limited to the 413 infants with 450k and covariate data. This study was approved by the Duke Institutional Review Board. Additional details about NEST may be found in previous publications.^53, 54^

***Childhood BMI***

Height and weight were collected at multiple points throughout childhood from medical records, direct measurement by study staff, and parental report. The current analysis used the measurement at the oldest age within each age range. BMI was calculated as kg/m^2^.

***DNA methylation measurements***

Genomic DNA from buffy coat specimens was extracted from umbilical cord blood using Puregene Reagents (Qiagen, Valencia, CA). Bisulfite conversion was performed using the EZ-96 DNA Methylation Kit (Zymo Research Corporation) and DNA methylation was measured at 485,577 CpGs using Illumina Infinium HumanMethylation450 BeadChip (Illumina Inc., San Diego, USA). Illumina’s GenomeStudio Methylation module version 1.0 (Illumina Inc.) was used to calculate the methylation level at each CpG as the beta value. Probe and sample-specific quality control was performed in the NEST cohort using a similar approach to MoBa1 and MoBa2 cohorts. Specifically, control probes (N=65) and probes on X (N=11 230) and Y (N=416) chromosomes were excluded as well as CpGs missing > 10% of methylation data. Samples indicated by Illumina to have failed or have an average detection P-value across all probes < 0.05 and samples with gender mismatch were also removed. The two different probe designs by applying the intra-array normalization strategy Beta Mixture Quantile dilation (BMIQ).^13^ The final dataset contained information on 473,586 CpGs for 215 samples in ages 2-5 years and 473,605 for 169 samples in ages 5-10 years.

***Covariates***

The gestational age and birth weight of the child were collected from medical records following delivery. Maternal smoking status, socioeconomic status (education), age, prepregnancy body mass index (BMI), parity, and race were reported by the mother on a questionnaire completed during pregnancy. Models were stratified by race/ethnicity group (Caucasian or African American).

***Cell type correction***

The Reinius-based Houseman method^6, 14^ was used with the estimateCellCounts function in the Minfi package^5^ in R^15^ to estimate relative proportions of six white blood cell subtypes (CD4+ T-lymphocytes, CD8+ T-lymphocytes, NK (natural killer) cells, B-lymphocytes, monocytes and granulocytes).

***Batch correction***

The Empirical Bayes method via ComBat was applied for batch correction using the sva package in R.^43^

## The Northern Finland Birth Cohorts (NFBC1986)

***Design and study population***

The Northern Finland Birth Cohort 1986 consists of 99% of all children, who were born in the provinces of Oulu and Lapland in Northern Finland between 1 July 1985 and 30 June 1986. 9203 live-born individuals entered the study.^55^ At the age of 16, the subjects living in the original target area or in the major cities of Finland (n=9215) were invited to participate a follow-up study including a clinical examination. 7344 participants attend the study in year 2001/2002, of which 5654 completed the postal questionnaire, the clinical examination and provided a blood sample.^56^

Approval for the studies was granted by the ethics committee of the Northern Ostrobothnia Hospital District in Oulu, Finland in accordance with the declaration of Helsinki.

***Adolescent BMI***

Height and weight were measured by the study nurse during clinical examination in the 16 year follow-up. Measurements were taken without shoes and in light clothing. BMI was calculated as weight (kg)/height (m^2^).

***DNA methylation measurements***

DNA was extracted from blood samples taken at the clinical examination during the 16 year follow-up in 2001-2002. Methylation of genomic DNA was quantified using the Illumina HumanMethylation450 array according to manufacturer’s instructions. Bisulfite conversion of genomic DNA was performed using the EZ DNA methylation kit according to manufacturer's instructions (Zymo Research, Orange, CA). DNA methylation was recoded on Illumina HumanMethlation450K array for 566 randomly selected subjects. Pre-processing of the methylation data was carried out using the functional normalization method.^57^ 24 technical replicates were excluded. 18 samples did not reach a call rate of >95% applying a detection P-value filter of 10-16 .We excluded 7 samples with gender inconsistency. After the additional 3IQR trimming, the final dataset contained information on 466290 CpGs for 344 samples.

***Covariates***

Maternal age, educational level, parity and smoking habits as well as height and pre-pregnancy weight were defined from questionnaires collected from the mothers during pregnancy. Smoking habits during pregnancy were transformed into a binary smoking status variable. Height and pre-pregnancy weight were used to calculate maternal BMI. Gestational age was estimated based on last menstrual period for all subjects and for some confirmed with ultrasound. Adolescent smoking and puberty status were self-reported at the 16 year follow-up study. Both girls and boys answered to two questions with descriptions and illustrations regarding their pubertal developmental stage, comparable to Tanner stages.

***Cell type correction***

Houseman estimates for blood cell composition were obtained by the minfi package for R.

***Batch correction***

We did not perform additional batch correction after functional normalization.

## Prevention and Incidence of Asthma and Mite Allergy (PIAMA)

The PIAMA study is a birth cohort study of children born between 1996-1997. Details of the study design have been published previously.^58^ In brief, pregnant women were recruited during their first trimester from the general population in 1996-1997 through antenatal clinics in the north, west and center of the Netherlands. Non-allergic pregnant women were invited to participate in a ‘‘natural history’’ study arm. Pregnant women identified as allergic through the screening questionnaire were allocated primarily to an intervention arm with a random subset allocated to the natural history arm. The intervention involved the use of mite-impermeable mattress and pillow covers. The study started with 3,963 newborns. Parents completed questionnaires on demographic factors, risk factors for asthma and respiratory symptoms at the child’s age of 3 months, annually from 1 to 8 years of age, and at 11, 14, and 16 years of age. Clinical examinations were performed in subgroups at ages 4, 8, 12 and 16 years.

***Childhood and adolescent BMI***

Childhood weight and height were measured at the clinical examination of 8 years old. Adolescent weight and height was measured at the clinical examination of 16 years old. BMI of both childhood and adolescent then were calculated as kg/m^2^ and transferred to sex- and age-adjusted standard deviation scores.

***DNA methylation measurements***

DNA from peripheral blood samples was extracted using the QIAamp blood kit (Qiagen), followed by a precipitation-based concentration using GlycoBlue (Ambion). DNA concentration was determined by Nanodrop measurement and picogreen quantification. 500 ng of DNA was bisulfite-converted using the EZ 96-DNA methylation kit (Zymo Research, Irvine, CA, USA), following the manufacturer’s standard protocol. DNA concentration was determined by Nanodrop measurement and Picogreen quantification. 500 ng of DNA was bisulphite-converted using the EZ 96-DNA methylation kit (Zymo Research), following the manufacturer’s standard protocol. After verification of the bisulphite conversion step using Sanger Sequencing, DNA concentration was normalized and the samples were randomized to avoid batch effects. One standard DNA sample per chip was included in this step for quality control. Genome-wide DNA methylation was measured using the Illumina Infinium HumanMethylation450 beadchip (450K array; Illumina, Inc., San Diego, CA, USA).

DNA methylation data were pre-processed in R with the Bioconductor package Minfi using the original IDAT files extracted from the HiScanSQ scanner.^5^ We had a total of 640 whole blood samples with DNA methylation data. We implemented sample filtering to remove bad quality samples (call rate <99%). Moreover, we used 65 SNP probes to check for concordances between paired DNA samples. Paired samples are the same individuals which also have match whole blood samples. Paired samples which show Pearson correlation coefficient <0.9 were regarded as sample mixed ups and were excluded from the study. During processing, the probes on sex chromosomes, the probes that mapped to multiple loci, 65 SNP-probes and the probes containing SNPs at the target CpG sites with a MAF>5% were excluded.^9^ We implemented “DASEN” to perform signal correction and normalization.^4^

After quality control, 207 samples of 8 years old and 439,306 autosomal probes remained. In analyses, 14 underweight participants are further excluded. In adolescent analyses, 613 samples of 16 years old and 436,824 probes remained, and 57 underweight participants and 37 with missing covariates are further excluded in analyses.

***Covariates***

Maternal age, gestational age and adolescent age were defined as continuous variables. Maternal social class was defined as the highest attained educational level and coded in three categories: 1=primary school, lower vocational or lower secondary education (low) 2=intermediate vocational education or intermediate/higher secondary education (intermediate) 3= higher vocational education and university (high). Parity was defined as older siblings living in the PIAMA home. Pre-pregnancy maternal BMI was calculated using height and weight of the mother before pregnancy, self-reported in the questionnaire when the child was 1 year of age. Maternal smoking during pregnancy was coded as 0= no smoking during pregnancy, 1= any smoking during pregnancy. Breastfeeding was coded as 1= any breastfeeding, 2=no breastfeeding. Adolescent smoking status was codes as 1= any adolescent smoking, 0 = no adolescent smoking.

***Cell type correction***

Cell type correction was applied using the reference-based Houseman method in the minfi Package in R.^5^ This method estimates the relative proportions of six white blood cell subtypes (CD4+ T-lymphocytes, CD8+ T-lymphocytes, NK (natural killer) cells, B-lymphocytes, monocytes and granulocytes).

***Batch correction***

For 16 years data, the samples were run into two batches, and therefore batch was included in the model. In the analysis of adolescent, we also corrected the study centers where the samples from.

## Prediction and Prevention of Preeclampsia and Intrauterine Growth Restriction study (PREDO)

***Design and study population***

Data were from the Prediction and Prevention of Preeclampsia and Intrauterine Growth Restriction (PREDO) Study, which is a longitudinal multicenter pregnancy cohort study of Finnish women and their singleton children born alive between 2006-2010.^59^ We recruited 1079 pregnant women, of whom 969 had one or more and 110 had none of the known risk factors for preeclampsia and intrauterine growth restriction. The recruitment took place in arrival order when these women attended the first ultrasound screening at 12+0-13+6 weeks+days of gestation in one of the ten hospital maternity clinics participating in the study. The cohort profile contains details of the study design and inclusion criteria.^59^ The study protocol was approved by the Ethical Committees of the Helsinki and Uusimaa Hospital District and by the participating hospitals. A written informed consent was obtained from all women. The study has been registered as ClinicalTrials.gov identifier ISRCTN14030412.

***Childhood BMI***

Weight and height of the child were extracted from the child welfare clinic growth charts..

***DNA methylation measurements***

The Predo Samples were ran on Illumina 450K Methylation arrays. The quality control pipeline was set up using the R-package minfi. Three participants were excluded as they were outliers in the median intensities. Furthermore, 20 participants showed disconcordance between phenotypic sex and estimated sex and were excluded. Methylation beta-values were normalized using the funnorm function. We excluded any probes on chromosome X or Y, probes containing SNPs and cross-hybridizing probes.^9^ Furthermore, any Cgs with a detection p-value > 0.01 in at least 50% of the samples were excluded. The final dataset contains 428,619 CpGs and 834 participants. Extreme outliers in the methylation set were trimmed according to the 3IQR method described in the project plan. Of these participants, 290 had valid data on childhood BMI between 24 months of age and 60 months of age. Sixty participants were excluded due to gestational diabetes mellitus and four due to type 1 diabetes. The final dataset contained information on 428619 CpGs in cord blood samples.

***Covariates***

Data on maternal age in years, educational level and parity were collected by questionnaires in early pregnancy. Maternal body mass index was assessed at intake and was extracted from the hospital records or Medical Birth Records (MBR). Gestational age was measured with ultrasound measurement at the first trimester and was extracted from the hospital records or MBR. Child sex, birthweight, and maternal smoking during pregnancy were derived from hospital records or MBR.

To control for the potential effects of population structure, genotyping was performed on Illumina Human Omni Express Exome Arrays (Illumina Inc., San Diego, CA). Only markers with a call rate of at least 98%, minor allele frequency of 1% and a p value for deviation from Hardy–Weinberg equilibrium > × 10–06 were kept in the analysis. We performed multidimensional scaling (MDS) analysis on the identity by state matrix of quality controlled genotypes. In the statistical models to control for the population structure, we adjusted for the first three MDS components.

***Cell type correction***

Cord blood cell counts were estimated for six cell types (granulocytes, monocytes, natural killer cells, B cells, CD4(+)T cells, and CD8(+)T cells) using the method of Houseman et al. which is incorporated in the R-package *minfi.*

***Batch correction***

We used ComBat to check and adjust for the batch effects (slide and well).

## Project Viva

***Design and study population***

Project Viva is a population-based prospective birth cohort in Eastern Massachusetts. Pregnant women at <22 weeks gestation with the ability to answer questions in English and singleton pregnancies were recruited at their first prenatal visit at a participating obstetric office from 1999 to 2002. Of 2218 live births, 1018 cord blood samples were collected. All women gave written informed consent for the study, and 507 mothers additionally gave written informed genetic consent. Institutional review boards at all participating institutions gave approval for this study.^60^

***Childhood BMI***

At early childhood (~3 years) and mid-childhood (~7 years) in-person visits, Project Viva research assistants measured weight using a calibrated scale (Seca model 881; Seca, Hanover, MD) and height using a calibrated stadiometer (Shorr Productions, Olney, MD).

***DNA methylation measurements***

At delivery, obstetricians and midwives collected blood from the umbilical cord vein from approximately half of births. At the early childhood and mid-childhood visits, trained research assistants (RAs) collected a blood sample from the antecubital vein. All samples were refrigerated immediately, processed within 24 hours (including extraction of DNA using Qiagen Puregene Kit, Valencia, CA), and stored at -80^o^C until time of analysis. We bisulfite-converted buffy coat DNA (EZ DNA Methylation-Gold Kit, Zymo Research, Irvine, CA) in cord blood, and in peripheral leukocytes from early and mid-childhood. We shipped samples to Illumina Inc., where they were analyzed using the Infinium HumanMethylation450 BeadChip (Illumina, San Diego, CA). Upon receiving the data, we removed technical replicates, samples with low quality, genotype mismatches, and sex mismatches. We also excluded low-quality probes with detection *P*-values >0.05, those on sex chromosomes, non-CpG probes, and non-specific and previously-identified cross-reactive probes. We further removed probes within 10 base pairs of a known SNP (UCSC Human Feb. 2009 [GRCh37/hg19] Assembly), and those with a minor allele frequency ≥1%. After using the pfilter function in the wateRmelon R package (pnthresh = 0.05 divided by the number of probes), we removed 6838 probes. Futhermore, we removed 11648 allosomal probes using the Illumina450ProbeVariants.db bioconductor annotation package from Tiffany Morris and 3091 CpH probes. The final dataset contained information on 470,870 CpGs for 293 (early childhood, crude analysis) and 271 (mid-childhood, crude analysis) samples in cord blood.

***Covariates***

Using a combination of self-administered questionnaires and interviews, we collected information about maternal education (categorized as ≥college graduate yes/no), pregnancy smoking status (categorized as ever smoked during pregnancy yes/no), and parity (categorized as nulliparous yes/no).

We calculated gestational age by using the date of the last menstrual period, but if the early second-trimester ultrasound assessment differed from the calculated gestational age by more than 10 days, we used the ultrasound dating instead. We obtained infant sex, birthweight, and date of birth from medical records.

We included maternal age, maternal education, maternal smoking, gestational age, parity, birth weight, and child BMI as covariates in the ComBat adjustment.

***Cell type correction***

Cell type composition at each research visit was estimated from genome-wide DNA methylation arrays (HumanMethylation450 BeadChip; Illumina) using the *minfi* package in R (version 3.3.0, R Core Team). We used an adult reference DNA methylation panel to estimate leukocyte composition (monocytes, granulocytes, B cells, CD4 T cells, CD8 T cells, and natural killer cells) using the estimateCellCounts function in the minfi R package.^6^

## The West Australian Pregnancy Cohort (Raine)

***Design and study population***

## The Raine Study enrolled pregnant women ≤18 weeks gestation (1989-1991) through the antenatal clinic at King Edward Memorial Hospital and nearby private clinics in Perth, Western Australia.^61, 62^ Detailed clinical assessments were performed at birth (n=2,868) and the children (Raine Generation 2) followed up at multiple time points including at 17 years of age, when a blood sample, anthropometric measures, and questionnaires were collected. The Human Ethics Committees of King Edward Memorial Hospital and Princess Margaret Hospital approved all protocols.

***Adolescent BMI***

Height and weight were measured with light clothing and without shoes. Height was measured with Holtain Infantometer and Stadiometer (to the nearest 0.1 cm), and weight was measured on Wedderburn Scales (to the nearest 100 ).^63^

***DNA methylation measurements***

For this methylation study we used data from the Raine Study Generation 2 at 17 years follow-up using DNA from whole blood. Epigenome-wide DNA methylation profiles of 1192 (58 technical replicates) individuals were examined using the Illumina Infinium HumanMethylation450 BeadChip array (Illumina San Diego, CA) and was done at the Centre for Molecular Medicine and Therapeutics (http://www.cmmt.ubc.ca). Quality control of the samples was done using the R statistical packages; shinyMethyl and MethylAid.^41, 42^ Three samples were outliers based on these two packages. Gender was inferred using the RnBeads R package and identified a discrepancy for a single sample.^64^ Fifty-eight of the samples were run in duplicate or triplicate and 65 SNPs present on the array were used to assess genetic similarity between individuals and one contaminated sample was excluded. Intentional SNP CpGs (n=65), sex chromosome CpGs (n=11,648) and CpGs with a detection p-value > 0.05 in any sample (n=10,777) were removed. A further 160 probes with low bead counts (bead counts less than 3 in more than 5% of samples) were removed. Principal component analysis was performed on the top 20,000 most variable probes and permutation tests were used to test for association between the top 10 principal components and experimental variables. Beta-mixture quantile normalisation (BMIQ) was applied^13^ to each CpG and batch effects were still present, therefore were accounted for in all models across a total of 462,297 CpGs.

***Covariates***

Data on maternal age, maternal education level, parity and maternal smoking during pregnancy were assessed by questionnaires at 18 and 34 weeks pregnancy. Smoking at adolescence was obtained by questionnaire at age 17. Maternal education is based on the following categories: 0=None, 1=Trade certificate or apprenticeship, 2=Professional registration (non-degree), 3=College diploma or degree, 4= University degree, 5=Other. Breastfeeding is based on the variable age when breastfeeding was stopped: 0=Never breastfed (stopped at 0 months), 1=Ever breastfed (stopped at any timepoint > 0 months of breast-feeding). To account for ancestry we used the first two principal components from our GWAS as covariates in all models.

***Cell type correction***

Potential confounding effects of blood cell subtypes were estimated by the reference-based Houseman method.^6^

***Batch correction***

We used plate and plate position as technical batch variables in all models.

## The Swedish Twin study On Prediction and Prevention of Asthma (STOPPA)

***Design and study population***

The Swedish Twin study On Prediction and Prevention of Asthma (STOPPA) is a twin cohort study including 752 individuals.^65^ Twins 9-14 years of age were selected from an on-going data collection within the Child and Adolescent Twin study in Sweden (CATSS) based on the pair’s asthma status.^66^ Asthma concordant (ACC), asthma discordant (ADC) and healthy concordant (HCC) pairs were included and invited to take part in test centre visits including clinical examination, questionnaires, lung function testing and collection of biosamples. The study was approved by the regional ethical review board in Stockholm, Sweden. Written informed consent was collected from the study participants and their parents. Further details regarding STOPPA have been provided in a separate publication.^65^ To allow for both twins from full pairs to be retained within the sample, generalized estimating equation (GEE) models were used in place of robust linear regression in STOPPA. By specifying twin pairs as clusters, the GEE method produces robust standard errors and corrects for within-cluster (i.e. within-pair) correlations. The parameter estimates themselves are not affected. For these analyses the *drgee* R package was used.^67^

***Adolescent BMI***

Information on height and weight was collected by a research nurse at the clinical examination and BMI calculated as weight (kg)/height (m^2^) and then age and sex adjusted using standard deviation scores as specified in the analysis plan.

***DNA methylation measurements***

DNA was extracted from whole blood (collected in a 4ml EDTA tube) using the Chemagic Star 400 kit (PerkinElmer chemagen, Baesweiler, Aachen, Germany) according to a standardized protocol. Samples were allocated between analysis plates and chips by complete randomization, with the exception that samples from twin pairs were kept within the same chip to allow for within-pair comparisons free of batch effects. Analyses were performed at the Mutation Analysis Facility (MAF) at Karolinska Institutet using the Infinium HumanMethylation450 Beadchip Kit (Illumina, Inc., San Diego, California, USA). Probe filtering and normalization was carried out using RnBeads package in R^15^ and the dasen method.^4^ Probes overlapping with single nucleotide polymorphisms or specific nucleotide contexts (15,392), due to unreliable measurements (defined as detection p-values > 5*10-8, resulting in the filtering of n=5,659 probes and 0 samples), or which were located on sex chromosomes (n=10,919) were filtered out, leaving 453,706 CpG probes. After additional filtering using the 3IQR method, a final number of n=452,752 probes for 272 samples remained for analysis.

***Covariates***

This is a subset of the STOPPA cohort (n=272), age range 12-15 years, who had information on all the requested covariates. Because STOPPA does not have information on adolescent smoking, this covariate was not included in the adolescent models.

The case/control variable ( asthma yes/no ) upon which recruitment to STOPPA was based has been included as a covariate in all models. Because STOPPA is a twin cohort, multiple births and siblings were retained in the sample, but handled using a GEE model specifying twin pair number as the cluster variable. This adjusts the standard errors for the correlation between individuals within the sample. This was performed using the drgee R package.^67^ Puberty was classified as early or late depending on whether the individual had had menarche (females) or voice change (males). If the aforementioned event had occurred the individual was considered to be in late puberty, but if it had not they were considered to be in early puberty.

***Cell type correction***

We used the minfi package in R to correct cell type heterogeneity followed the Houseman algorithm.

***Batch correction***

We added the batch variables in all statistical models to adjust for the batch effects.

# References

1. Fraser A, Macdonald-Wallis C, Tilling K, Boyd A, Golding J, Davey Smith G, et al. Cohort Profile: the Avon Longitudinal Study of Parents and Children: ALSPAC mothers cohort. Int J Epidemiol. 2013;42(1):97-110.

2. Boyd A, Golding J, Macleod J, Lawlor DA, Fraser A, Henderson J, et al. Cohort Profile: the 'children of the 90s'--the index offspring of the Avon Longitudinal Study of Parents and Children. Int J Epidemiol. 2013;42(1):111-27.

3. Touleimat N, Tost J. Complete pipeline for Infinium((R)) Human Methylation 450K BeadChip data processing using subset quantile normalization for accurate DNA methylation estimation. Epigenomics. 2012;4(3):325-41.

4. Pidsley R, CC YW, Volta M, Lunnon K, Mill J, Schalkwyk LC. A data-driven approach to preprocessing Illumina 450K methylation array data. BMC Genomics. 2013;14:293.

5. Aryee MJ, Jaffe AE, Corrada-Bravo H, Ladd-Acosta C, Feinberg AP, Hansen KD, et al. Minfi: a flexible and comprehensive Bioconductor package for the analysis of Infinium DNA methylation microarrays. Bioinformatics. 2014;30(10):1363-9.

6. Houseman EA, Accomando WP, Koestler DC, Christensen BC, Marsit CJ, Nelson HH, et al. DNA methylation arrays as surrogate measures of cell mixture distribution. BMC Bioinformatics. 2012;13:86.

7. Wickman M, Kull I, Pershagen G, Nordvall SL. The BAMSE project: presentation of a prospective longitudinal birth cohort study. Pediatr Allergy Immunol. 2002;13(s15):11-3.

8. Cole TJ, Green PJ. Smoothing reference centile curves: the LMS method and penalized likelihood. Stat Med. 1992;11(10):1305-19.

9. Chen YA, Lemire M, Choufani S, Butcher DT, Grafodatskaya D, Zanke BW, et al. Discovery of cross-reactive probes and polymorphic CpGs in the Illumina Infinium HumanMethylation450 microarray. Epigenetics. 2013;8(2):203-9.

10. Petersen AC, Crockett L, Richards M, Boxer A. A self-report measure of pubertal status: Reliability, validity, and initial norms. J Youth Adolesc. 1988;17(2):117-33.

11. Eskenazi B, Harley K, Bradman A, Weltzien E, Jewell NP, Barr DB, et al. Association of in utero organophosphate pesticide exposure and fetal growth and length of gestation in an agricultural population. Environ Health Perspect. 2004;112(10):1116-24.

12. Yousefi P, Huen K, Schall RA, Decker A, Elboudwarej E, Quach H, et al. Considerations for normalization of DNA methylation data by Illumina 450K BeadChip assay in population studies. Epigenetics. 2013;8(11):1141-52.

13. Teschendorff AE, Marabita F, Lechner M, Bartlett T, Tegner J, Gomez-Cabrero D, et al. A beta-mixture quantile normalization method for correcting probe design bias in Illumina Infinium 450 k DNA methylation data. Bioinformatics. 2013;29(2):189-96.

14. Reinius LE, Acevedo N, Joerink M, Pershagen G, Dahlen SE, Greco D, et al. Differential DNA methylation in purified human blood cells: implications for cell lineage and studies on disease susceptibility. PLoS One. 2012;7(7):e41361.

15. R Core Team. R: A language and environment for statistical computing. Vienna, Austria: *R Foundation for Statistical Computing*; 2013.

16. Koletzko B, von Kries R, Closa R, Escribano J, Scaglioni S, Giovannini M, et al. Lower protein in infant formula is associated with lower weight up to age 2 y: a randomized clinical trial. Am J Clin Nutr. 2009;89(6):1836-45.

17. Weber M, Grote V, Closa-Monasterolo R, Escribano J, Langhendries JP, Dain E, et al. Lower protein content in infant formula reduces BMI and obesity risk at school age: follow-up of a randomized trial. Am J Clin Nutr. 2014;99(5):1041-51.

18. Rzehak P, Covic M, Saffery R, Reischl E, Wahl S, Grote V, et al. DNA-Methylation and Body Composition in Preschool Children: Epigenome-Wide-Analysis in the European Childhood Obesity Project (CHOP)-Study. Sci Rep. 2017;7(1):14349.

19. Rzehak P, Saffery R, Reischl E, Covic M, Wahl S, Grote V, et al. Maternal Smoking during Pregnancy and DNA-Methylation in Children at Age 5.5 Years: Epigenome-Wide-Analysis in the European Childhood Obesity Project (CHOP)-Study. PLoS One. 2016;11(5):e0155554.

20. Kirchberg FF, Harder U, Weber M, Grote V, Demmelmair H, Peissner W, et al. Dietary protein intake affects amino acid and acylcarnitine metabolism in infants aged 6 months. J Clin Endocrinol Metab. 2015;100(1):149-58.

21. R Core Team. R: A language and environment for statistical computing. R Foundation for Statistical Computing, Vienna, Austria 2013 [Available from: <http://www.R-project.org/>].

22. McConnell R, Berhane K, Yao L, Jerrett M, Lurmann F, Gilliland F, et al. Traffic, susceptibility, and childhood asthma. Environ Health Perspect. 2006;114(5):766-72.

23. Noushmehr H, Weisenberger DJ, Diefes K, Phillips HS, Pujara K, Berman BP, et al. Identification of a CpG island methylator phenotype that defines a distinct subgroup of glioma. Cancer Cell. 2010;17(5):510-22.

24. Triche TJ, Weisenberger DJ, Van Den Berg D, Laird PW, Siegmund KD. Low-level Processing of Illumina Infinium DNA Methylation BeadArrays. Nucleic Acids Res. 2013;(in press).

25. Bolstad BM, Irizarry RA, Astrand M, Speed TP. A comparison of normalization methods for high density oligonucleotide array data based on variance and bias. Bioinformatics. 2003;19(2):185-93.

26. Pritchard JK, Stephens M, Donnelly P. Inference of population structure using multilocus genotype data. Genetics. 2000;155(2):945-59.

27. Triche TJ, Jr., Weisenberger DJ, Van Den Berg D, Laird PW, Siegmund KD. Low-level processing of Illumina Infinium DNA Methylation BeadArrays. Nucleic Acids Res. 2013;41(7):e90.

28. Maksimovic J, Gordon L, Oshlack A. SWAN: Subset-quantile within array normalization for illumina infinium HumanMethylation450 BeadChips. Genome Biol. 2012;13(6):R44.

29. L'Abee C, Sauer PJ, Damen M, Rake JP, Cats H, Stolk RP. Cohort Profile: the GECKO Drenthe study, overweight programming during early childhood. Int J Epidemiol. 2008;37(3):486-9.

30. Kupers LK, Xu X, Jankipersadsing SA, Vaez A, la Bastide-van Gemert S, Scholtens S, et al. DNA methylation mediates the effect of maternal smoking during pregnancy on birthweight of the offspring. Int J Epidemiol. 2015;44(4):1224-37.

31. Kooijman MN, Kruithof CJ, van Duijn CM, Duijts L, Franco OH, van IMH, et al. The Generation R Study: design and cohort update 2017. Eur J Epidemiol. 2016;31(12):1243-64.

32. Genome of the Netherlands C. Whole-genome sequence variation, population structure and demographic history of the Dutch population. Nat Genet. 2014;46(8):818-25.

33. Bonder MJ, Kasela S, Kals M, Tamm R, Lokk K, Barragan I, et al. Genetic and epigenetic regulation of gene expression in fetal and adult human livers. BMC Genomics. 2014;15:860.

34. Gaillard R, Steegers EA, de Jongste JC, Hofman A, Jaddoe VW. Tracking of fetal growth characteristics during different trimesters and the risks of adverse birth outcomes. Int J Epidemiol. 2014;43(4):1140-53.

35. Paternoster L, Evans DM, Nohr EA, Holst C, Gaborieau V, Brennan P, et al. Genome-wide population-based association study of extremely overweight young adults--the GOYA study. PLoS One. 2011;6(9):e24303.

36. Starling AP, Brinton JT, Glueck DH, Shapiro AL, Harrod CS, Lynch AM, et al. Associations of maternal BMI and gestational weight gain with neonatal adiposity in the Healthy Start study. Am J Clin Nutr. 2015;101(2):302-9.

37. Shapiro AL, Schmiege SJ, Brinton JT, Glueck D, Crume TL, Friedman JE, et al. Testing the fuel-mediated hypothesis: maternal insulin resistance and glucose mediate the association between maternal and neonatal adiposity, the Healthy Start study. Diabetologia. 2015;58(5):937-41.

38. Maitre L, de Bont J, Casas M, Robinson O, Aasvang GM, Agier L, et al. Human Early Life Exposome (HELIX) study: a European population-based exposome cohort. BMJ Open. 2018;8(9):e021311.

39. Vrijheid M, Slama R, Robinson O, Chatzi L, Coen M, van den Hazel P, et al. The human early-life exposome (HELIX): project rationale and design. Environ Health Perspect. 2014;122(6):535-44.

40. Lehne B, Drong AW, Loh M, Zhang W, Scott WR, Tan ST, et al. A coherent approach for analysis of the Illumina HumanMethylation450 BeadChip improves data quality and performance in epigenome-wide association studies. Genome Biol. 2015;16:37.

41. van Iterson M, Tobi EW, Slieker RC, den Hollander W, Luijk R, Slagboom PE, et al. MethylAid: visual and interactive quality control of large Illumina 450k datasets. Bioinformatics. 2014;30(23):3435-7.

42. Fortin JP, Fertig E, Hansen K. shinyMethyl: interactive quality control of Illumina 450k DNA methylation arrays in R. F1000Res. 2014;3:175.

43. Johnson WE, Li C, Rabinovic A. Adjusting batch effects in microarray expression data using empirical Bayes methods. Biostatistics. 2007;8(1):118-27.

44. Guxens M, Ballester F, Espada M, Fernandez MF, Grimalt JO, Ibarluzea J, et al. Cohort Profile: the INMA--INfancia y Medio Ambiente--(Environment and Childhood) Project. Int J Epidemiol. 2012;41(4):930-40.

45. Fernandez-Barres S, Romaguera D, Valvi D, Martinez D, Vioque J, Navarrete-Munoz EM, et al. Mediterranean dietary pattern in pregnant women and offspring risk of overweight and abdominal obesity in early childhood: the INMA birth cohort study. Pediatr Obes. 2016;11(6):491-9.

46. Arshad SH, Karmaus W, Zhang H, Holloway JW. Multigenerational cohorts in patients with asthma and allergy. J Allergy Clin Immunol. 2017;139(2):415-21.

47. Jaffe AE, Irizarry RA. Accounting for cellular heterogeneity is critical in epigenome-wide association studies. Genome Biol. 2014;15(2):R31.

48. Magnus P, Birke C, Vejrup K, Haugan A, Alsaker E, Daltveit AK, et al. Cohort Profile Update: The Norwegian Mother and Child Cohort Study (MoBa). Int J Epidemiol. 2016;45(2):382-8.

49. Haberg SE, London SJ, Nafstad P, Nilsen RM, Ueland PM, Vollset SE, et al. Maternal folate levels in pregnancy and asthma in children at age 3 years. J Allergy Clin Immunol. 2011;127(1):262-4, 4 e1.

50. Joubert BR, Haberg SE, Nilsen RM, Wang X, Vollset SE, Murphy SK, et al. 450K epigenome-wide scan identifies differential DNA methylation in newborns related to maternal smoking during pregnancy. Environ Health Perspect. 2012;120(10):1425-31.

51. Joubert BR, Felix JF, Yousefi P, Bakulski KM, Just AC, Breton C, et al. DNA Methylation in Newborns and Maternal Smoking in Pregnancy: Genome-wide Consortium Meta-analysis. Am J Hum Genet. 2016;98(4):680-96.

52. Paltiel L, Haugan A, Skjerden T, Harbak K, Baekken S, Stensrud NK, et al. The biobank of the Norwegian Mother and Child Cohort STudy - present status. Norsk Epidemiologi. 2014;24(29):29-35.

53. Hoyo C, Murtha AP, Schildkraut JM, Forman MR, Calingaert B, Demark-Wahnefried W, et al. Folic acid supplementation before and during pregnancy in the Newborn Epigenetics STudy (NEST). BMC Public Health. 2011;11(1):46.

54. Hoyo C, Murtha AP, Schildkraut JM, Jirtle RL, Demark-Wahnefried W, Forman MR, et al. Methylation variation at IGF2 differentially methylated regions and maternal folic acid use before and during pregnancy. Epigenetics. 2011;6(7):928-36.

55. Jarvelin MR, Hartikainen-Sorri AL, Rantakallio P. Labour induction policy in hospitals of different levels of specialisation. Br J Obstet Gynaecol. 1993;100(4):310-5.

56. Jaaskelainen A, Schwab U, Kolehmainen M, Kaakinen M, Savolainen MJ, Froguel P, et al. Meal frequencies modify the effect of common genetic variants on body mass index in adolescents of the northern Finland birth cohort 1986. PLoS One. 2013;8(9):e73802.

57. Fortin JP, Labbe A, Lemire M, Zanke BW, Hudson TJ, Fertig EJ, et al. Functional normalization of 450k methylation array data improves replication in large cancer studies. Genome Biol. 2014;15(12):503.

58. Wijga AH, Kerkhof M, Gehring U, de Jongste JC, Postma DS, Aalberse RC, et al. Cohort profile: the prevention and incidence of asthma and mite allergy (PIAMA) birth cohort. Int J Epidemiol. 2014;43(2):527-35.

59. Girchenko P, Lahti M, Tuovinen S, Savolainen K, Lahti J, Binder EB, et al. Cohort Profile: Prediction and prevention of preeclampsia and intrauterine growth restriction (PREDO) study. Int J Epidemiol. 2017;46(5):1380-1g.

60. Oken E, Baccarelli AA, Gold DR, Kleinman KP, Litonjua AA, De Meo D, et al. Cohort profile: project viva. Int J Epidemiol. 2015;44(1):37-48.

61. Newnham JP, Evans SF, Michael CA, Stanley FJ, Landau LI. Effects of frequent ultrasound during pregnancy: a randomised controlled trial. Lancet. 1993;342(8876):887-91.

62. Straker L, Mountain J, Jacques A, White S, Smith A, Landau L, et al. Cohort Profile: The Western Australian Pregnancy Cohort (Raine) Study-Generation 2. Int J Epidemiol. 2017;46(5):1384-5j.

63. Huang RC, Burrows S, Mori TA, Oddy WH, Beilin LJ. Lifecourse Adiposity and Blood Pressure Between Birth and 17 Years Old. Am J Hypertens. 2015;28(8):1056-63.

64. Assenov Y, Muller F, Lutsik P, Walter J, Lengauer T, Bock C. Comprehensive analysis of DNA methylation data with RnBeads. Nat Methods. 2014;11(11):1138-40.

65. Almqvist C, Ortqvist AK, Ullemar V, Lundholm C, Lichtenstein P, Magnusson PK. Cohort Profile: Swedish Twin Study on Prediction and Prevention of Asthma (STOPPA). Twin Res Hum Genet. 2015;18(3):273-80.

66. Anckarsater H, Lundstrom S, Kollberg L, Kerekes N, Palm C, Carlstrom E, et al. The Child and Adolescent Twin Study in Sweden (CATSS). Twin Res Hum Genet. 2011;14(6):495-508.

67. Zetterqvist J, Sjolander A. Doubly robust estimation with the R package drgee. Epidemiol Method. 2015;4:69-86.
