# Supplementary material for: DNA methylation and body mass index from birth to adolescence: meta-analyses of epigenome-wide association studies
Source: Genome Med. 2020 Nov 25;12:105. doi: 10.1186/s13073-020-00810-w (PMC7687793; doi:10.1186/s13073-020-00810-w)
Supplement: Supplementary file 5 — Additional file 5: Supplementary Information. Figures S1 – 6. [file 13073_2020_810_MOESM5_ESM.docx]

**Supplementary Information**

**DNA methylation and body mass index from birth to adolescence:**

**meta-analyses of epigenome-wide association studies**

Vehmeijer et al.

**Fig. S1A-D.** QQ plots and genomic inflation factors (lambdas) of all four analyses: the associations of DNA methylation in cord blood with early childhood BMI (Fig. S1A) and late childhood BMI (Fig. S1B), of DNA methylation in whole blood in childhood with childhood BMI (Fig. S1C) and of DNA methylation in whole blood in adolescence with adolescent BMI (Fig. S1D).

**Fig. S2A-D.** Volcano plots showing methylation levels in association with childhood or adolescent BMI of all four analyses: the associations of DNA methylation in cord blood with early childhood BMI (Fig. S2A) and late childhood BMI (Fig. S2B), of DNA methylation in whole blood in childhood with childhood BMI (Fig. S2C) and of DNA methylation in whole blood in adolescence with adolescent BMI (Fig. S2D).

**Fig. S3A-L.** Forest plots for the genome-wide Bonferroni- and FDR- significantly associated CpGs in the analyses of cord blood DNA methylation and later childhood BMI (Fig. S3A), DNA methylation in whole blood in childhood and childhood BMI (Fig. S3B-K) and DNA methylation in whole blood in adolescence and adolescent BMI (Fig. S3L).

**Fig. S4A-L.** Leave-one-out plots for the genome-wide Bonferroni- and FDR- significantly associated CpGs in the analyses of cord blood DNA methylation and later childhood BMI (Fig. S4A), DNA methylation in whole blood in childhood and childhood BMI (Fig. S4B-K) and DNA methylation in whole blood in adolescence and adolescent BMI (Fig. S4L), showing the results after omitting one study at a time.

**Fig. S5.** Boxplots showing the distribution of effect sizes of the 187 CpGs significantly associated with adult BMI in a previous study for the four analyses for the four models, separately for CpGs with positive and negative effect estimates in the original analysis.^1^

**Fig. S6A-D.** Density plots performed within the Generation R Study for those FDR-significant CpGs that are flagged in the main meta-analyses tables as potential polymorphic sites.

| **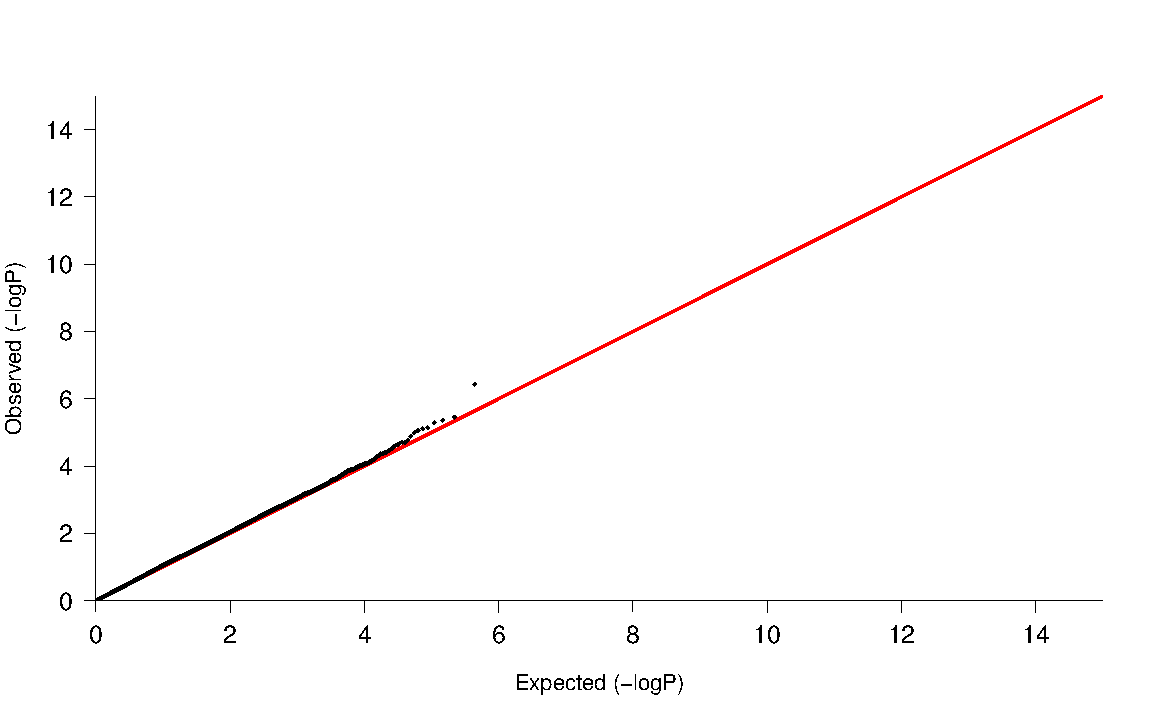Fig. S1A**  Lambda = 1.04  0.967866344616212 | **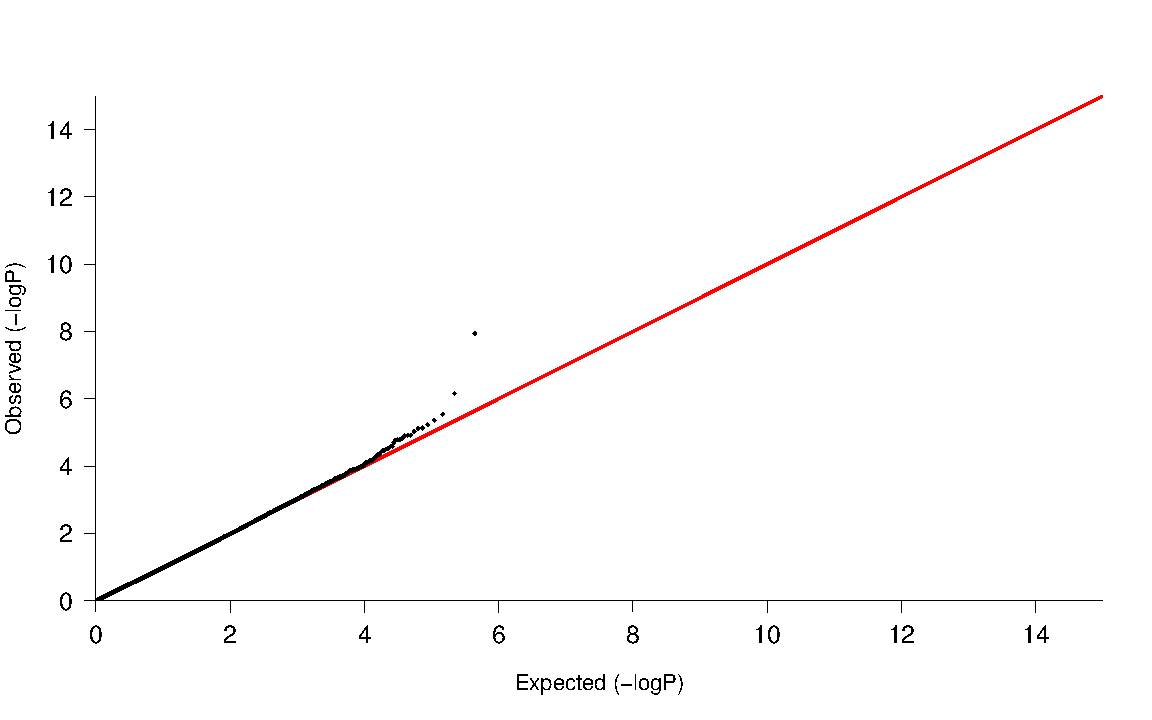Fig. S1B**  Lambda = 0.97  0.967866344616212 |
| --- | --- |
| 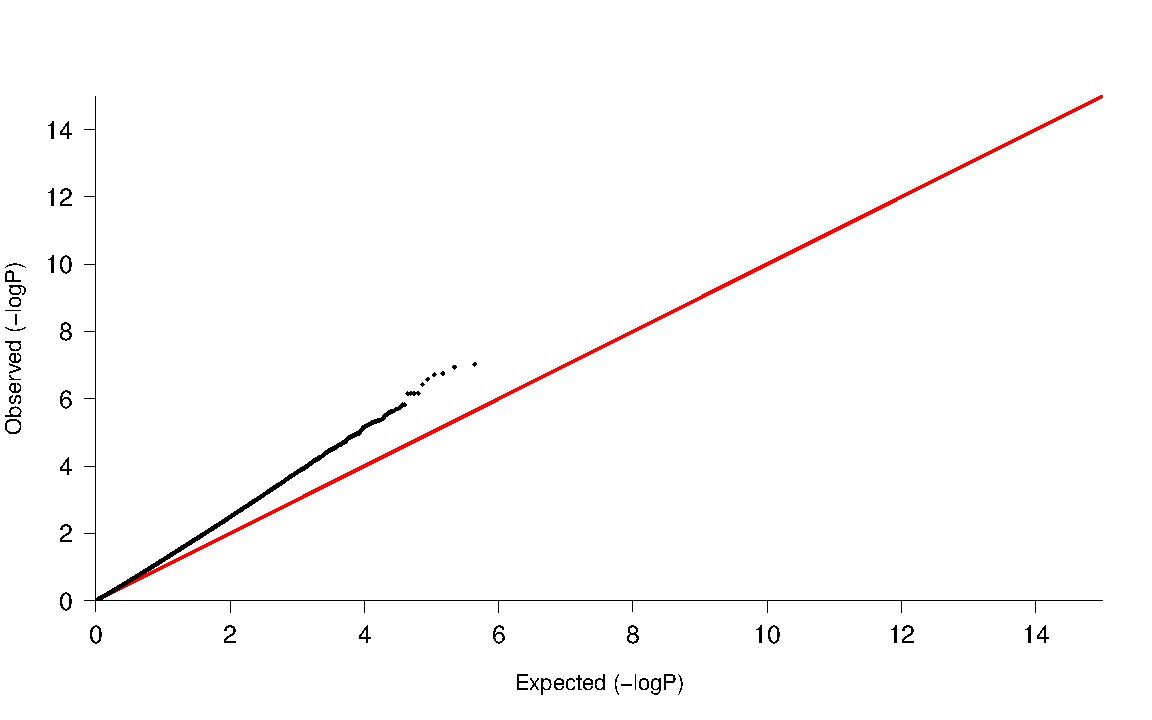  Lambda = 1.27  0.967866344616212  **Fig. S1C** | **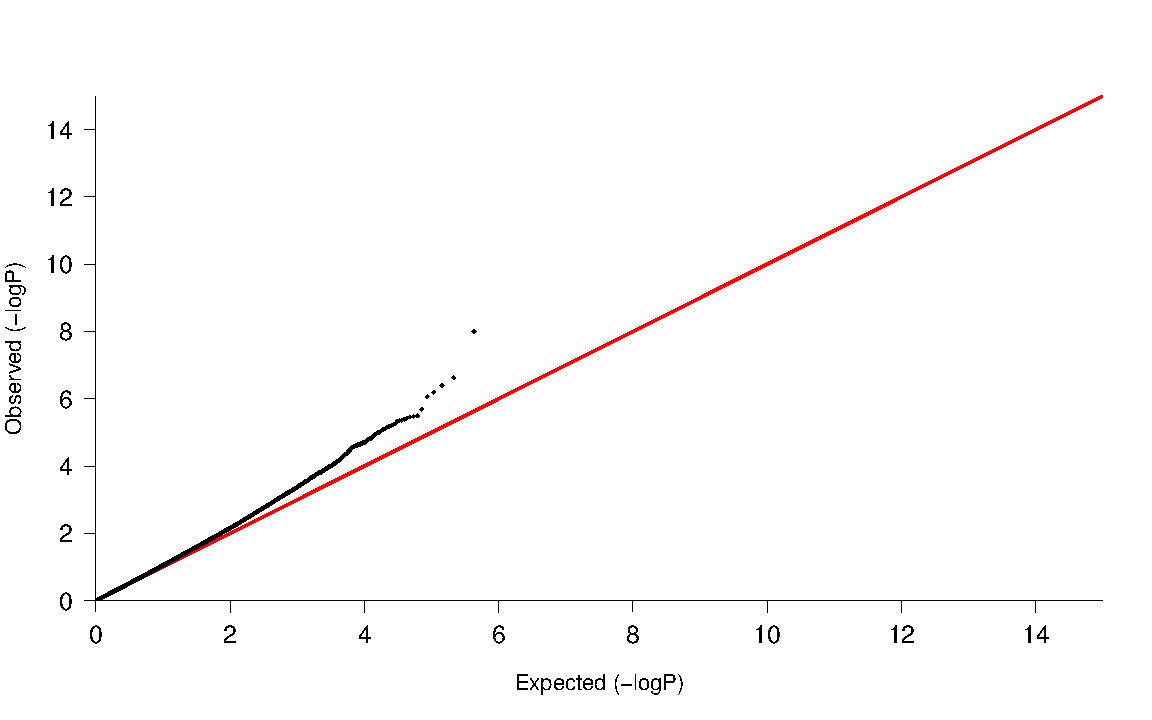Fig. S1D**  Lambda = 1.04  0.967866344616212 |

**Fig. S1A-D.** QQ plots and genomic inflation factors (lambdas) of all four analyses: the associations of DNA methylation in cord blood with early childhood BMI (Fig. S1A) and late childhood BMI (Fig. S1B), of DNA methylation in whole blood in childhood with childhood BMI (Fig. S1C) and of DNA methylation in whole blood in adolescence with adolescent BMI (Fig. 1D).

| 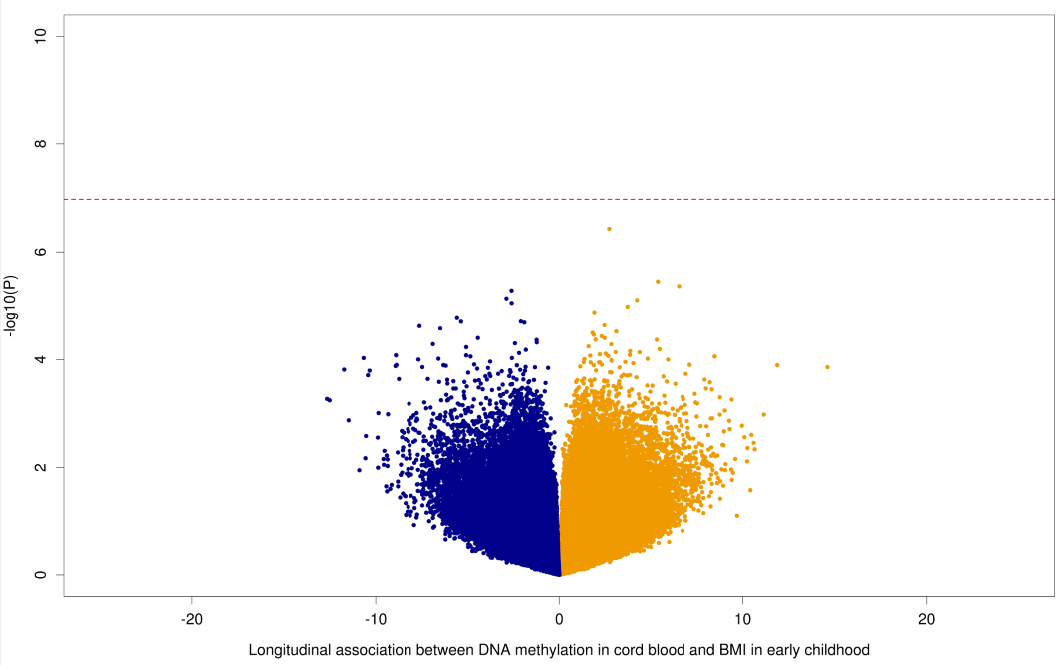  **Fig. S2A** | 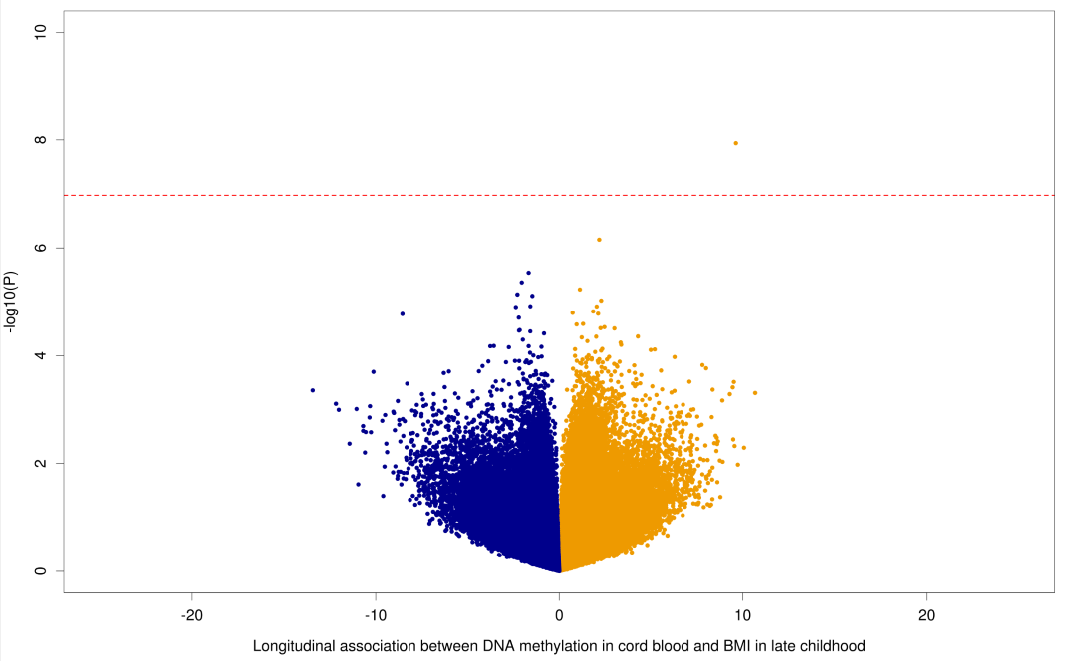  **Fig. S2B** |
| --- | --- |
| 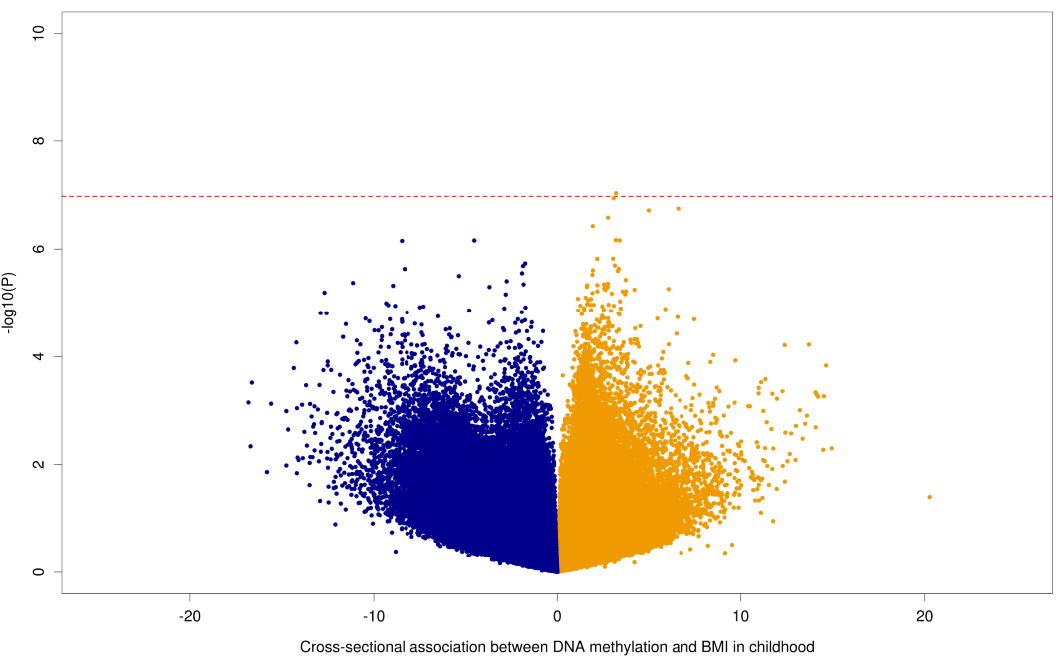  **Fig. S2C** | 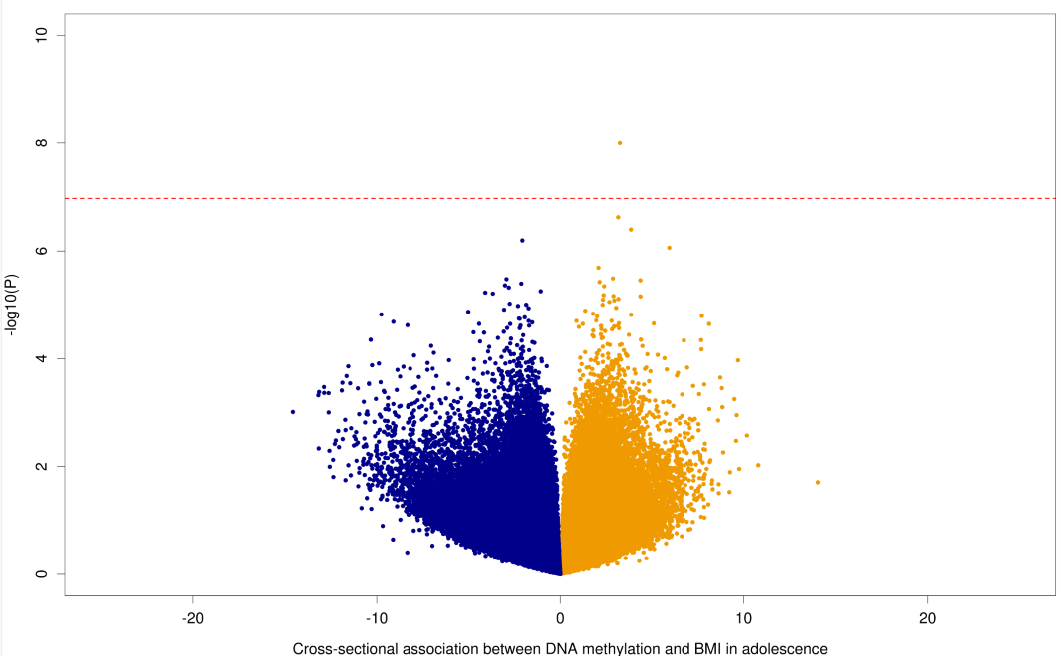**Fig. S2D** |

# Fig. S2A-D. Volcano plot for differential DNA methylation for all four analyses. The x-axes show the mean DNA methylation difference, while the y-axis shows the -log10 of the adjusted p-value for each CpG-site, representing the strength of the association. Above the dashed line indicates the CpGs that are statistically significant after Bonferroni correction (p< 1.06x10^-7^).

In analysis A, associations of DNA methylation in cordblood with early childhood BMI were positive for 222,659 CpGs (51.8%) and negative for 207,279 (48.2%).

In analysis B, associations of DNA methylation in cordblood with late childhood BMI were positive for 201,602 CpGs (46.9%) and negative for 228,327 CpGs (53.1%).

In analysis C, associations of DNA methylation in childhood with childhood BMI were positive for 217,326 CpGs (50.5%) and negative for 212,604 CpGs (49.4%).

In analysis D, associations of DNA methylation in adolescence with adolescent BMI were positive for 18,716 CpGs (51.0%) and negative for 210,229 CpGs (49.0%).


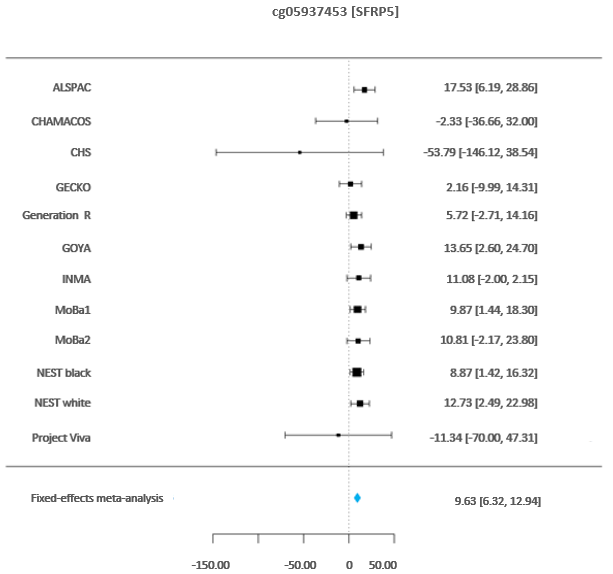


Change in late childhood BMI-standard deviation score per 100% increase in cord blood DNA methylation

**Fig. S3A.** Forest plot for the genome-wide significantly associated CpG (cg05937453) in the analysis of cord blood DNA methylation and late childhood BMI.


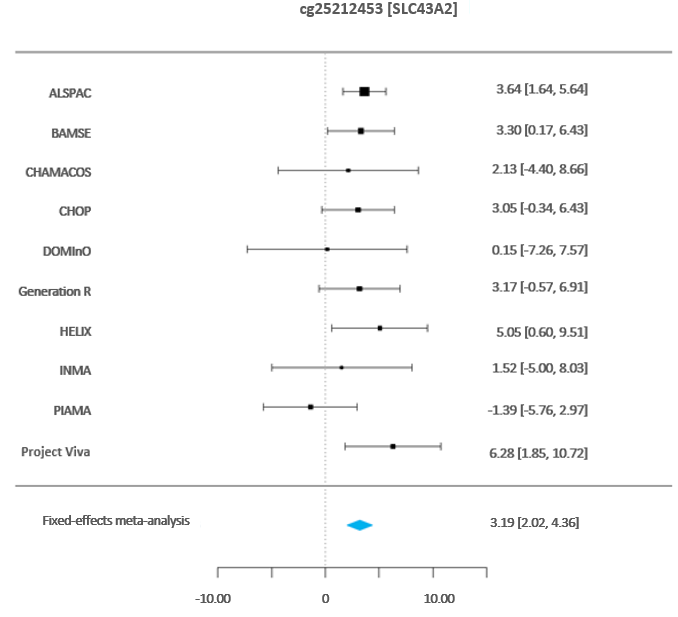


Change in childhood BMI-standard deviation score per 100% increase in childhood DNA methylation

**Fig. S3B.**  Forest plot for the genome-wide Bonferroni-significantly associated CpG (cg25212453) in the analysis of DNA methylation in whole blood in childhood and childhood BMI.


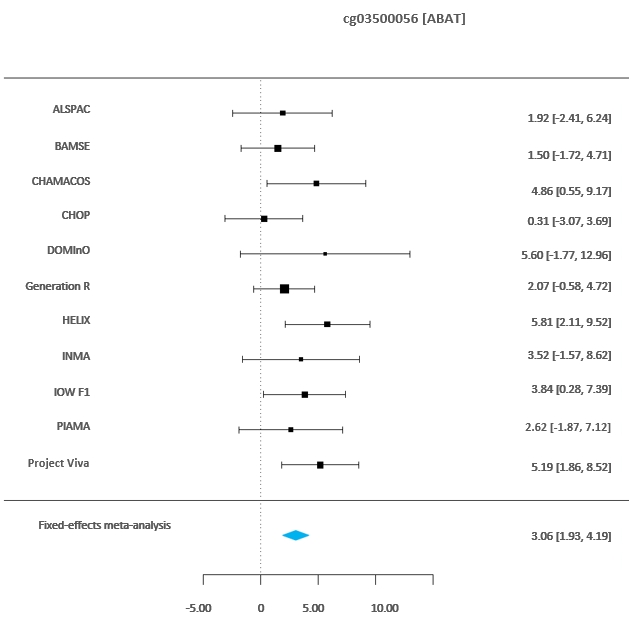


Change in childhood BMI-standard deviation score per 100% increase in childhood DNA methylation

**Fig. S3C.**  Forest plot for the genome-wide FDR-significantly associated CpG (cg03500056) in the analysis of DNA methylation in whole blood in childhood and childhood BMI.


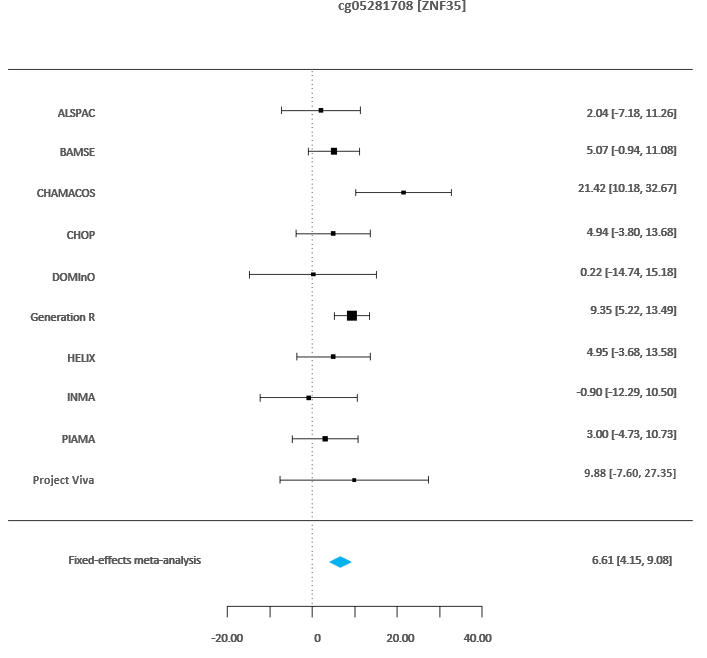


Change in childhood BMI-standard deviation score per 100% increase in childhood DNA methylation

**Fig. S3D.**  Forest plot for the genome-wide FDR-significantly associated CpG (cg05281708) in the analysis of DNA methylation in whole blood in childhood and childhood BMI.


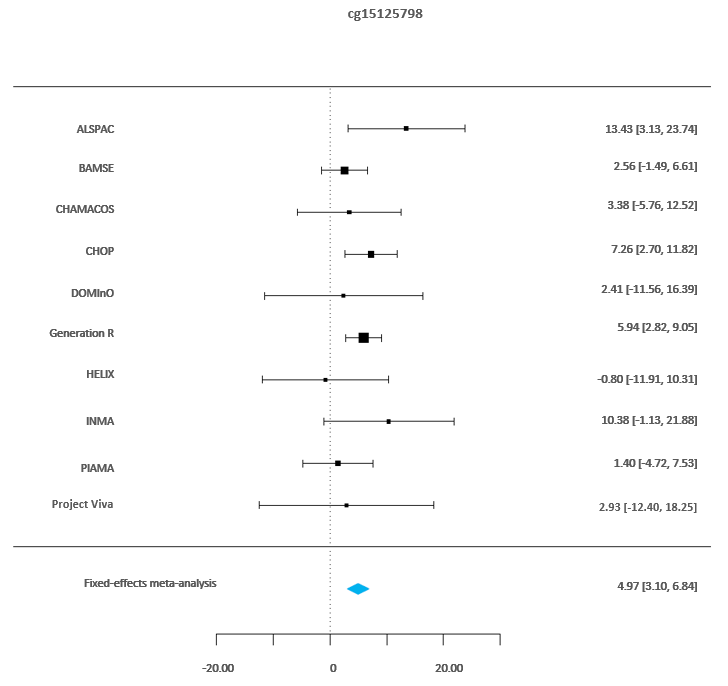


Change in childhood BMI-standard deviation score per 100% increase in childhood DNA methylation

**Fig. S3E.**  Forest plot for the genome-wide FDR-significantly associated CpG (cg15125798) in the analysis of DNA methylation in whole blood in childhood and childhood BMI.


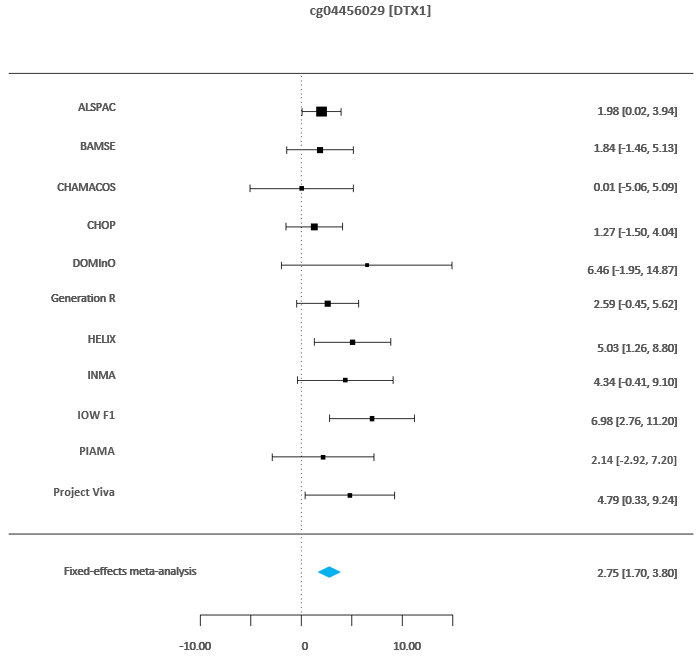


Change in childhood BMI-standard deviation score per 100% increase in childhood DNA methylation

**Fig. S3F.**  Forest plot for the genome-wide FDR-significantly associated CpG (cg04456029) in the analysis of DNA methylation in whole blood in childhood and childhood BMI.


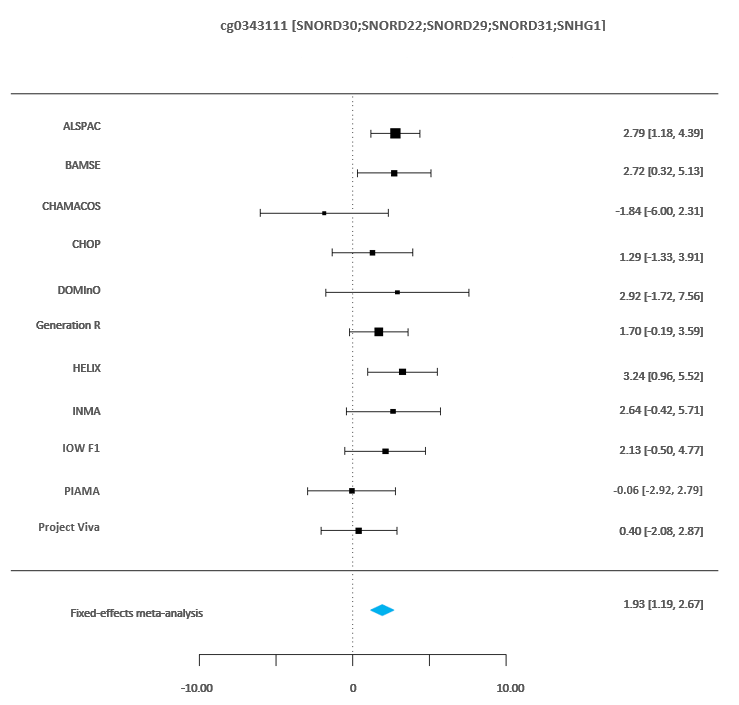


Change in childhood BMI-standard deviation score per 100% increase in childhood DNA methylation

**Fig. S3G.**  Forest plot for the genome-wide FDR-significantly associated CpG (cg0343111) in the analysis of DNA methylation in whole blood in childhood and childhood BMI.


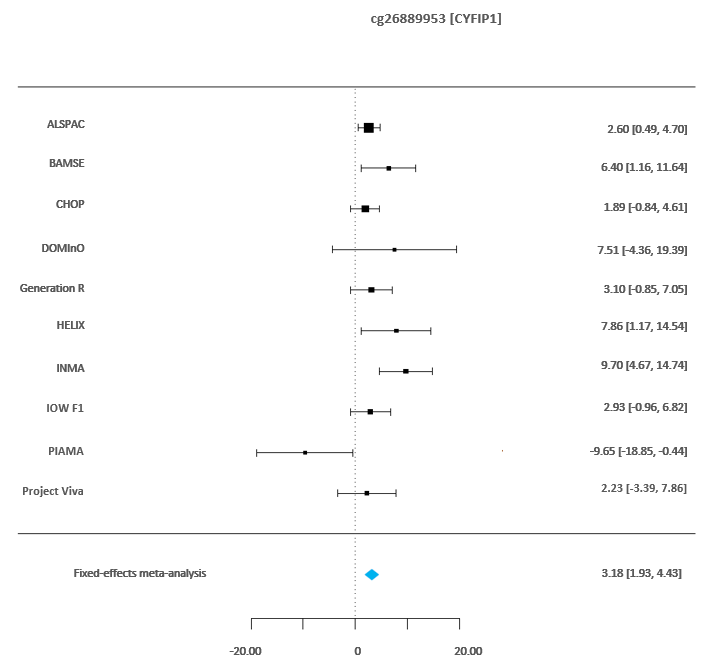


Change in childhood BMI-standard deviation score per 100% increase in childhood DNA methylation

**Fig. S3H.**  Forest plot for the genome-wide FDR-significantly associated CpG (cg26889953) in the analysis of DNA methylation in whole blood in childhood and childhood BMI.


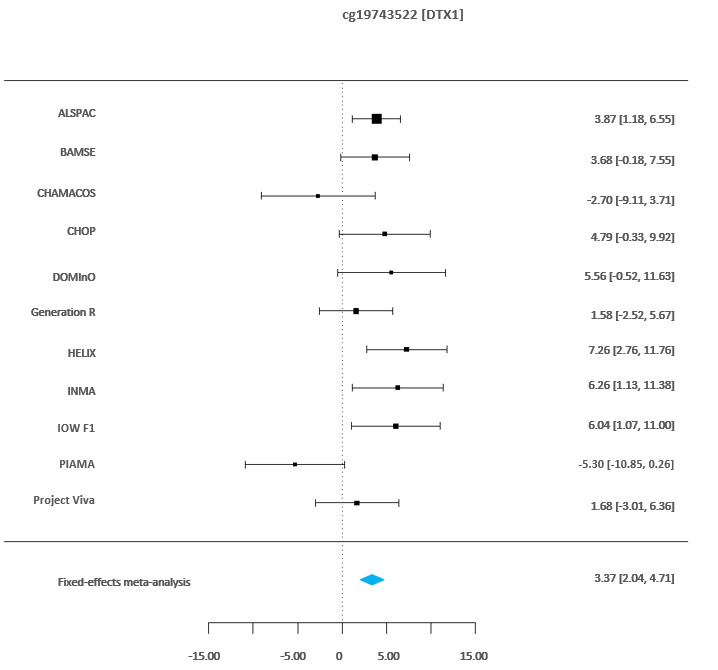


Change in childhood BMI-standard deviation score per 100% increase in childhood DNA methylation

**Fig. S3I.**  Forest plot for the genome-wide FDR-significantly associated CpG (cg19743522) in the analysis of DNA methylation in whole blood in childhood and childhood BMI.


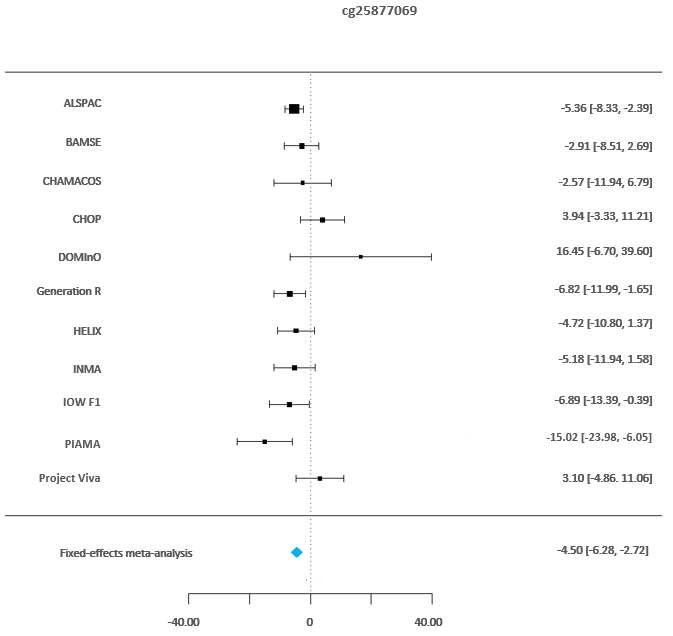


Change in childhood BMI-standard deviation score per 100% increase in childhood DNA methylation

**Fig. S3J.**  Forest plot for the genome-wide FDR-significantly associated CpG (cg25877069) in the analysis of DNA methylation in whole blood in childhood and childhood BMI.


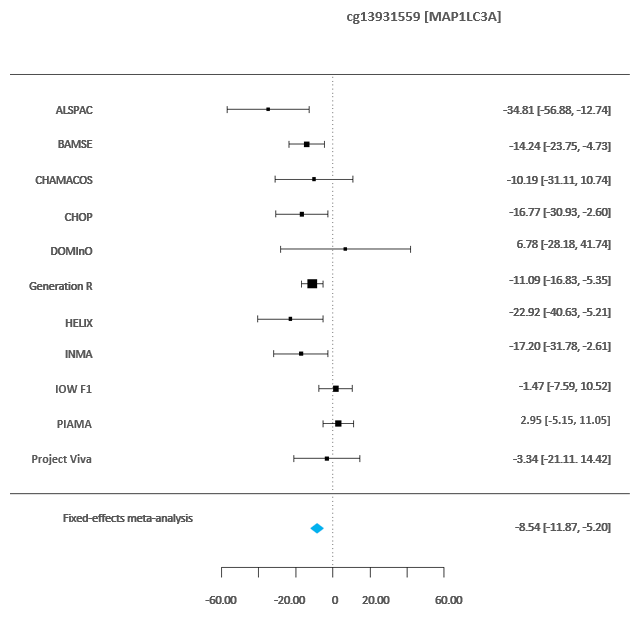


Change in childhood BMI-standard deviation score per 100% increase in childhood DNA methylation

**Fig. S3K.**  Forest plot for the genome-wide FDR-significantly associated CpG (cg13931559) in the analysis of DNA methylation in whole blood in childhood and childhood BMI.


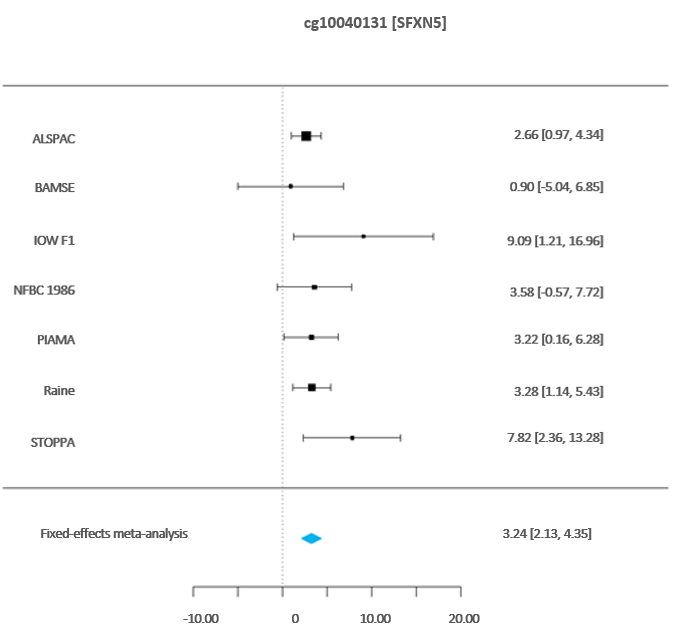


Change in adolescent BMI-standard deviation score per 100% increase in adolescent DNA methylation

**Fig. S3L.**  Forest plot for the genome-wide significantly associated CpG (cg10040131) in the analysis of DNA methylation in whole blood in adolescence and adolescent BMI.


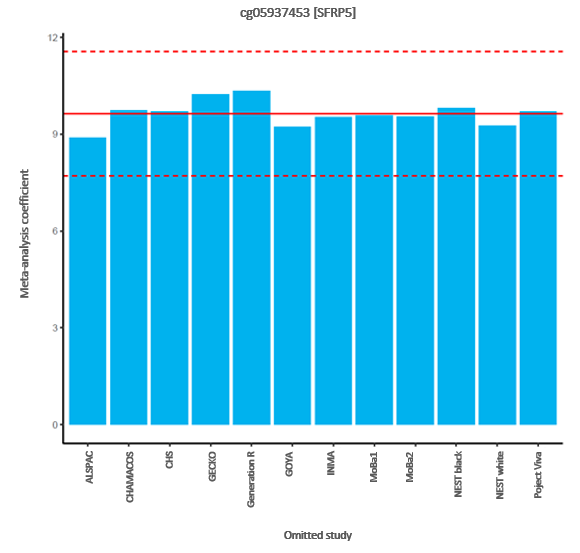


**Fig. S4A.** Leave-one-out plot for the genome-wide Bonferroni-significantly associated cg05937453, showing the association of methylation levels in cord blood with late childhood BMI, if the indicated study would be omitted from the meta-analysis. The red line represents the effect size of the full meta-analysis beta and the dotted red lines indicate the 20% range around that effect size.


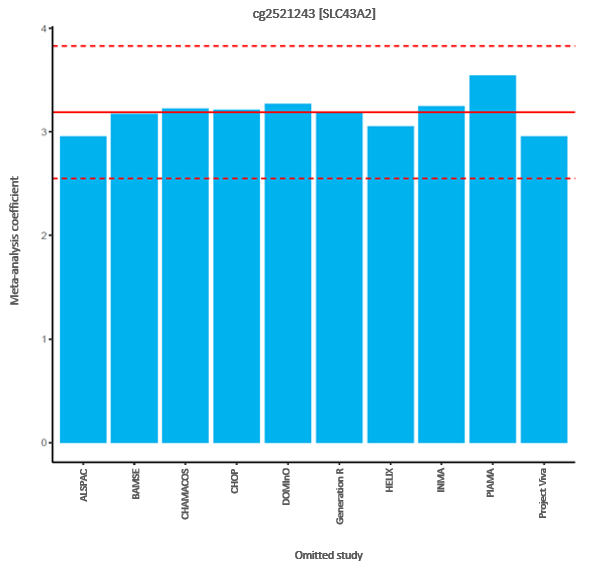


**Fig. S4B.** Leave-one-out plot for the genome-wide Bonferroni-significantly associated cg25212453, showing the association of methylation levels in whole blood in childhood with childhood BMI, if the indicated study would be omitted from the meta-analysis. The red line is the meta-analysis beta and the dotted red lines indicate the 20% range around the beta.


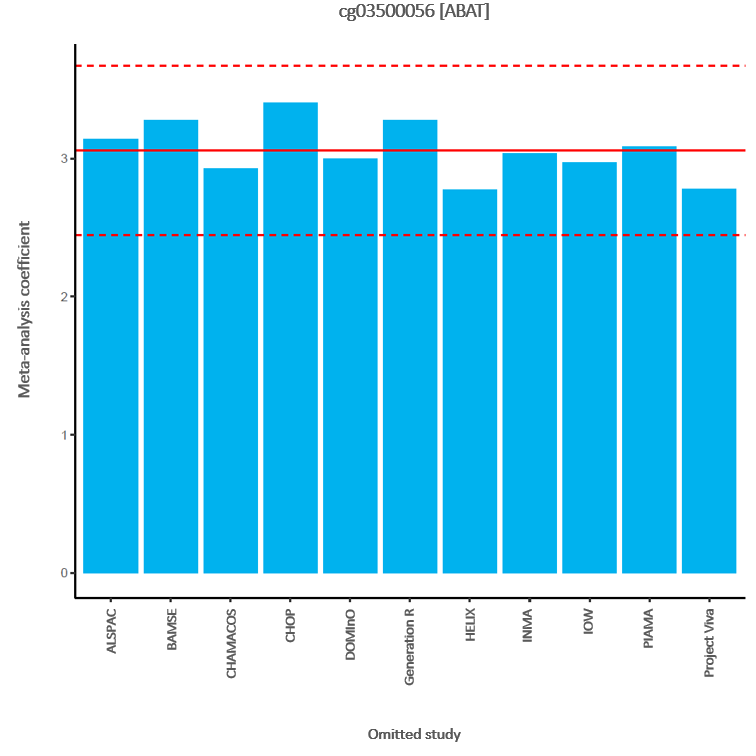


**Fig. S4C.** Leave-one-out plot for the genome-wide FDR-significantly associated cg03500056, showing the association of methylation levels in whole blood in childhood with childhood BMI, if the indicated study would be omitted from the meta-analysis. The red line is the meta-analysis beta and the dotted red lines indicate the 20% range around the beta.


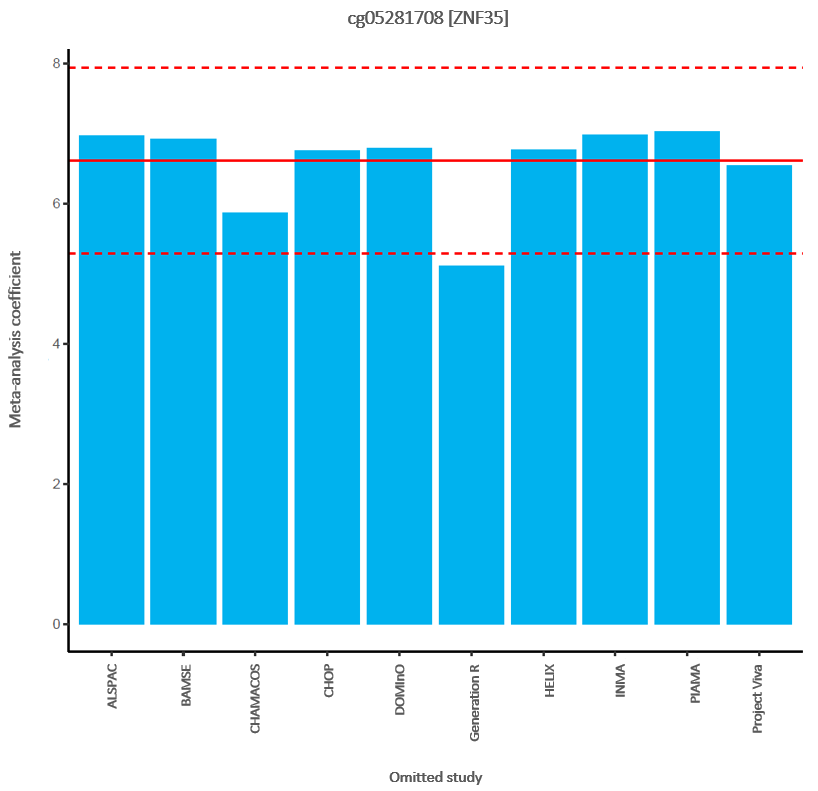


**Fig. S4D.** Leave-one-out plot for the genome-wide FDR-significantly associated cg05281708, showing the association of methylation levels in whole blood in childhood with childhood BMI, if the indicated study would be omitted from the meta-analysis. The red line is the meta-analysis beta and the dotted red lines indicate the 20% range around the beta.


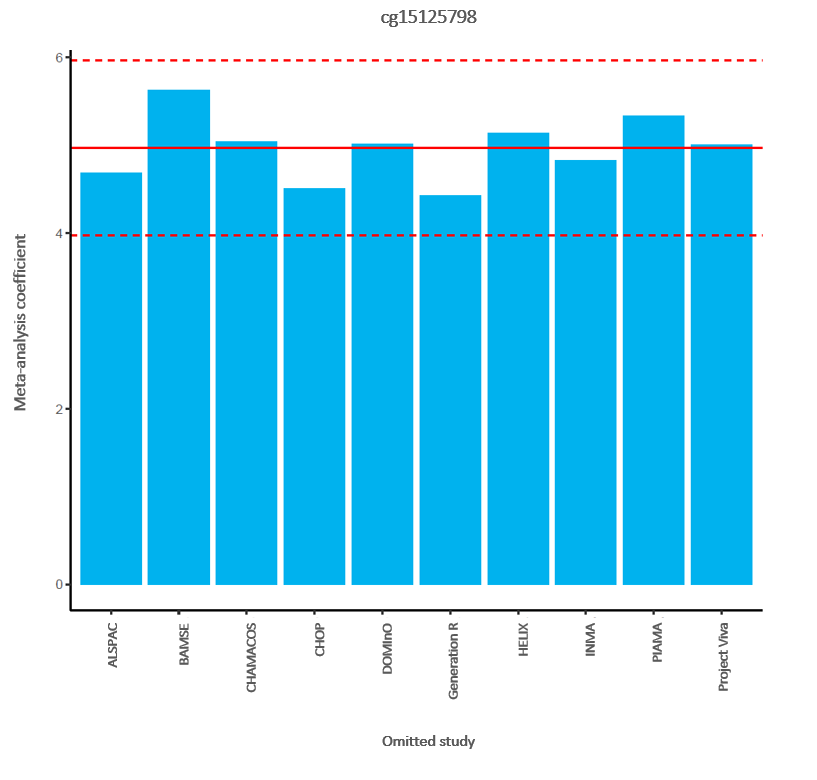


**Fig. S4E.** Leave-one-out plot for the genome-wide FDR-significantly associated cg15125798, showing the association of methylation levels in whole blood in childhood with childhood BMI, if the indicated study would be omitted from the meta-analysis. The red line is the meta-analysis beta and the dotted red lines indicate the 20% range around the beta.


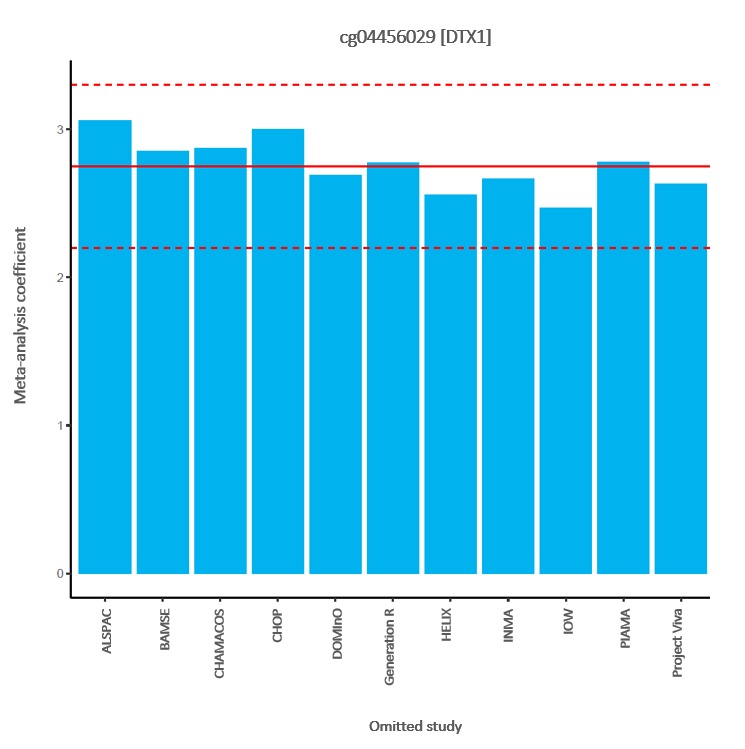


**Fig. S4F.** Leave-one-out plot for the genome-wide FDR-significantly associated cg04456029, showing the association of methylation levels in whole blood in childhood with childhood BMI, if the indicated study would be omitted from the meta-analysis. The red line is the meta-analysis beta and the dotted red lines indicate the 20% range around the beta.


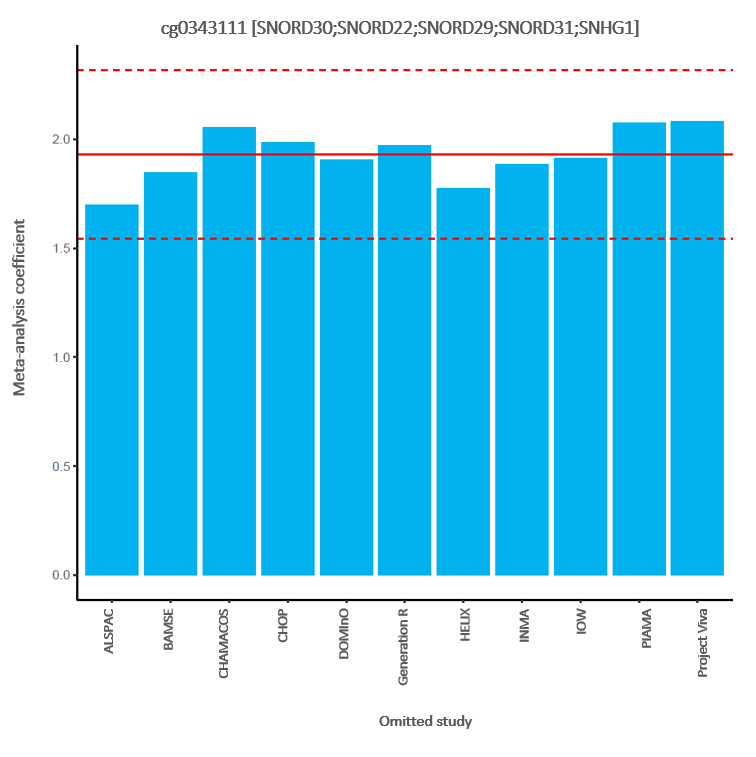


**Fig. S4G.** Leave-one-out plot for the genome-wide FDR-significantly associated cg0343111, showing the association of methylation levels in whole blood in childhood with childhood BMI, if the indicated study would be omitted from the meta-analysis. The red line is the meta-analysis beta and the dotted red lines indicate the 20% range around the beta.


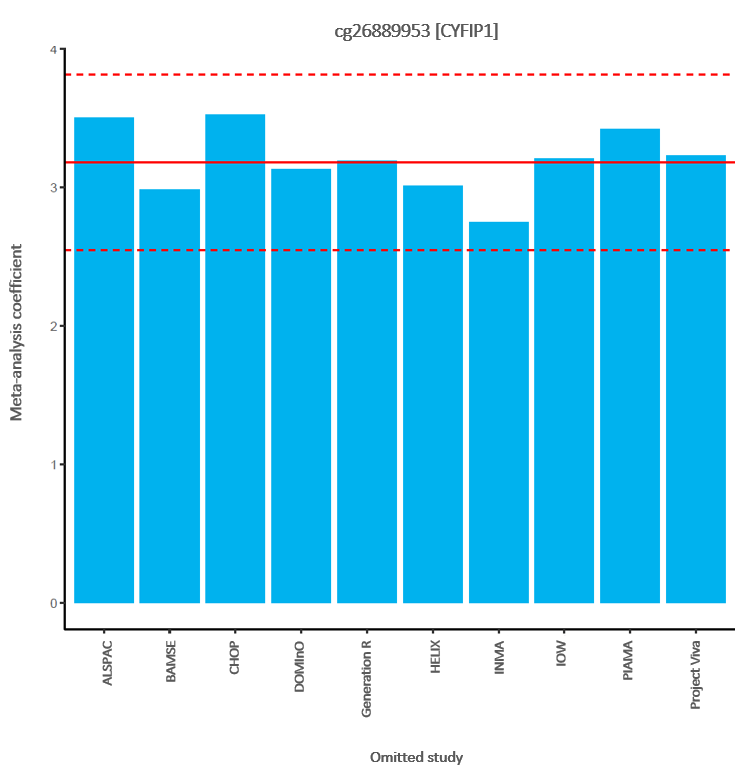


**Fig. S4H.** Leave-one-out plot for the genome-wide FDR-significantly associated cg26889953, showing the association of methylation levels in whole blood in childhood with childhood BMI, if the indicated study would be omitted from the meta-analysis. The red line is the meta-analysis beta and the dotted red lines indicate the 20% range around the beta.


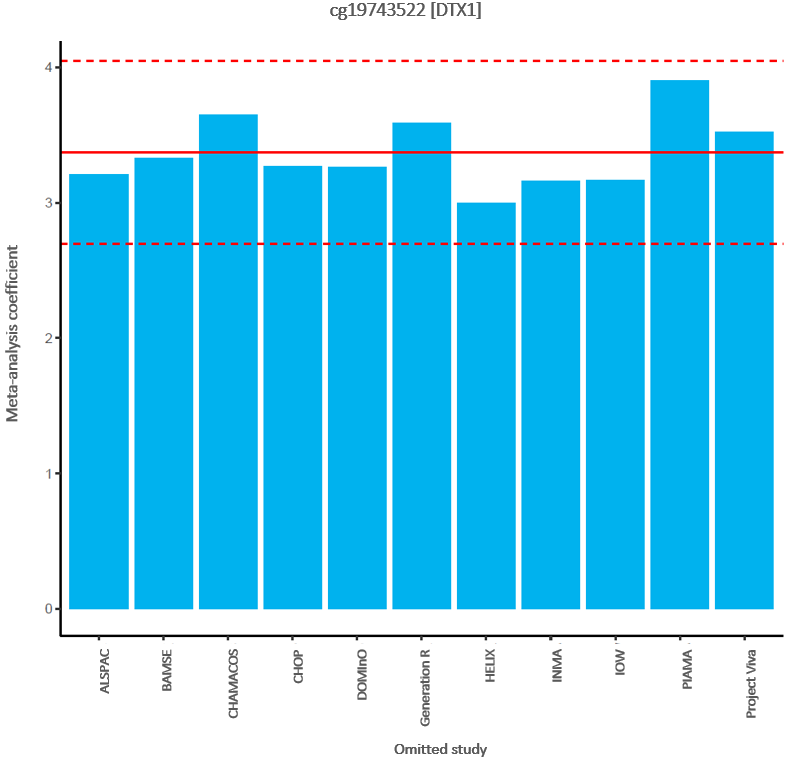


**Fig. S4I.** Leave-one-out plot for the genome-wide FDR-significantly associated cg19743522, showing the association of methylation levels in whole blood in childhood with childhood BMI, if the indicated study would be omitted from the meta-analysis. The red line is the meta-analysis beta and the dotted red lines indicate the 20% range around the beta.


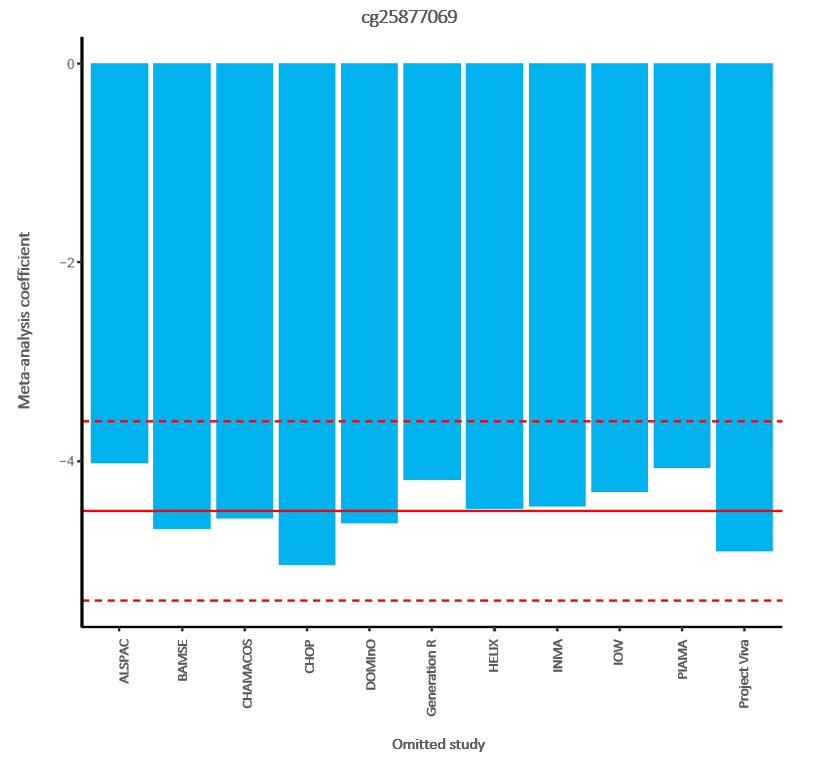


**Fig. S4J.** Leave-one-out plot for the genome-wide FDR-significantly associated cg25877069, showing the association of methylation levels in whole blood in childhood with childhood BMI, if the indicated study would be omitted from the meta-analysis. The red line is the meta-analysis beta and the dotted red lines indicate the 20% range around the beta.


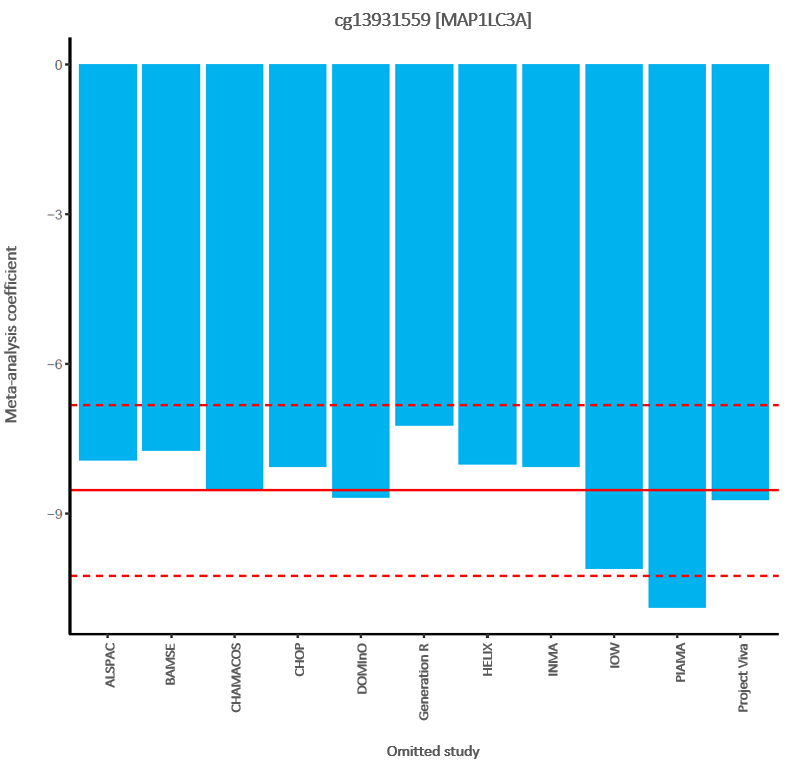


**Fig. S4K.** Leave-one-out plot for the genome-wide FDR-significantly associated cg13931559, showing the association of methylation levels in whole blood in childhood with childhood BMI, if the indicated study would be omitted from the meta-analysis. The red line is the meta-analysis beta and the dotted red lines indicate the 20% range around the beta.


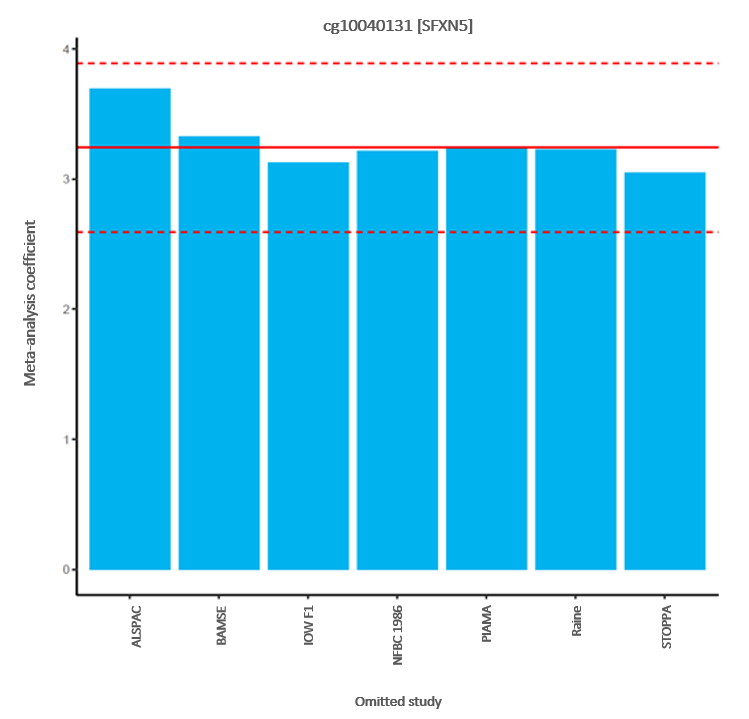


**Fig. S4L.** Leave-one-out plot for the genome-wide Bonferroni-significantly associated cg10040131, showing the association of methylation levels in whole blood in adolescence with adolescent BMI, if the indicated study would be omitted from the meta-analysis. The red line is the meta-analysis beta and the dotted red lines indicate the 20% range around the beta.


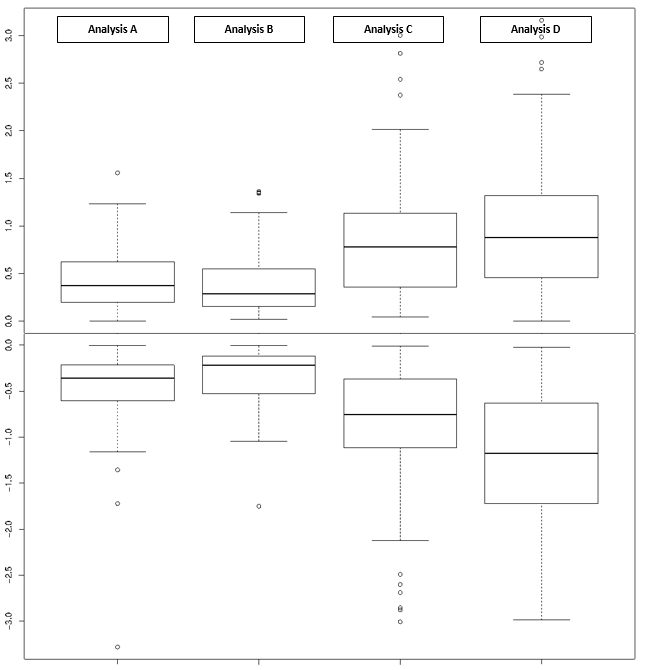


**Fig. S5.** Boxplots showing the distribution of effect sizes of the 187 CpGs significantly associated with adult BMI in a previous study for the four analyses: the associations of DNA methylation in cord blood with early childhood BMI (Analysis A) and late childhood BMI (Analysis B), of DNA methylation in whole blood in childhood with childhood BMI (Analysis C) and of DNA methylation in whole blood in adolescence with adolescent BMI (Analysis D). Results are shown separately for CpGs with positive and negative effest estimates in the original analysis.^1^

**
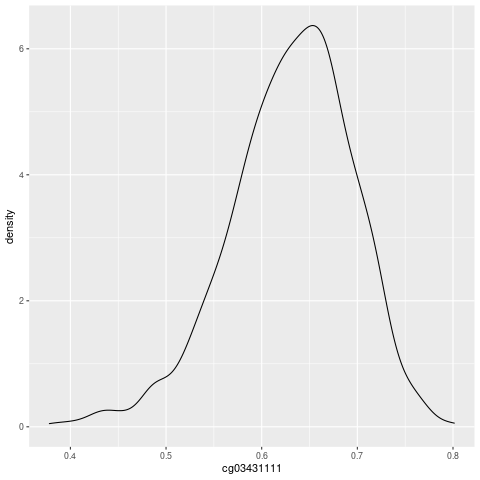
**

**Fig. S6A.** Density plot performed within the Generation R Study for the potentially polymorphic probe, the genome-wide FDR_significantly associated cg03431111 in analysis C (cross-sectional analysis of DNA methylation and BMI in childhood), showing no indication of a non-unimodal distribution.


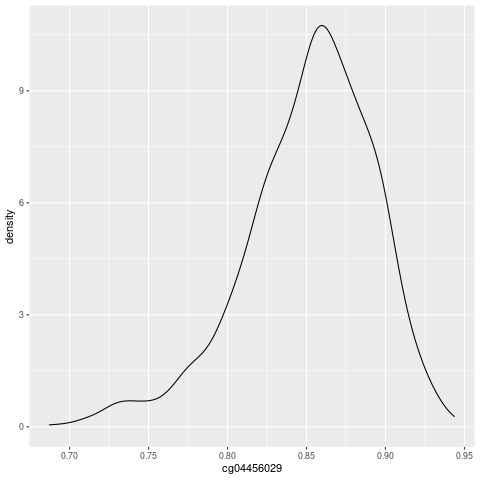


**Fig. S6B.** Density plot performed within the Generation R Study for the potentially polymorphic probe, the genome-wide FDR_significantly associated cg04456029 in analysis C (cross-sectional analysis of DNA methylation and BMI in childhood), showing no indication of a non-unimodal distribution.


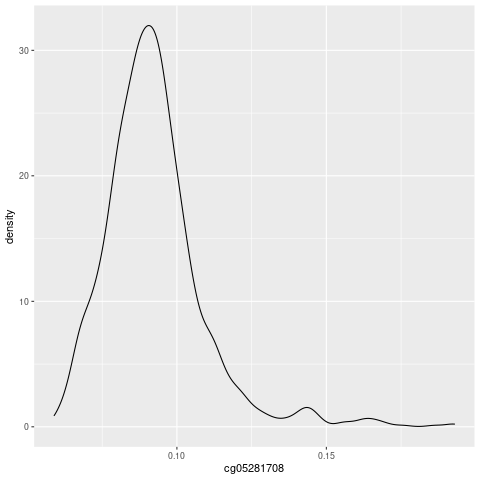


**Fig. S6C.** Density plot performed within the Generation R Study for the potentially polymorphic probe, the genome-wide FDR_significantly associated cg05281708 in analysis C (cross-sectional analysis of DNA methylation and BMI in childhood), showing no indication of a non-unimodal distribution.


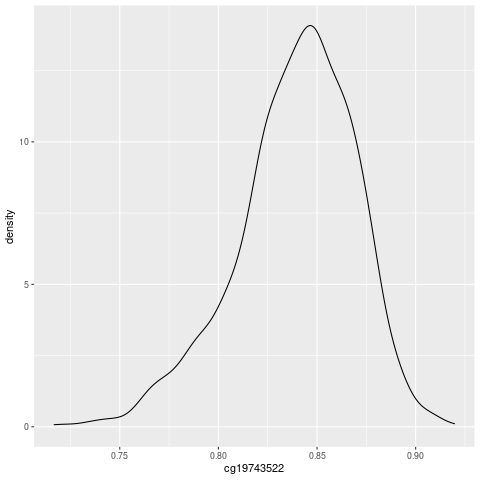


**Fig. S6D.** Density plot performed within the Generation R Study for the potentially polymorphic probe, the genome-wide FDR_significantly associated cg19743522 in analysis C (cross-sectional analysis of DNA methylation and BMI in childhood), showing no indication of a non-unimodal distribution.

**References**

1. Wahl S, Drong A, Lehne B, Loh M, Scott WR, Kunze S, et al. Epigenome-wide association study of body mass index, and the adverse outcomes of adiposity. Nature. 2017;541(7635):81-6.
